# Multicenter phase II trial of transanal total mesorectal excision for rectal cancer: preliminary results

**DOI:** 10.1007/s00464-023-10266-9

**Published:** 2023-09-12

**Authors:** Patricia Sylla, Dana Sands, Alison Ricardo, Antoinette Bonaccorso, Alexandros Polydorides, Mariana Berho, John Marks, Justin Maykel, Karim Alavi, Karen Zaghiyan, Mark Whiteford, Elisabeth Mclemore, Sami Chadi, Sherief F. Shawki, Scott Steele, Alessio Pigazzi, Matthew Albert, Teresa DeBeche-Adams, Erin Moshier, Steven D. Wexner

**Affiliations:** 1 Division of Colon and Rectal Surgery, Mount Sinai Hospital, New York, NY USA; 2 Department of Colon and Rectal Surgery, Cleveland Clinic Florida, Weston, FL USA; 3 Department of Pathology, Mount Sinai Hospital, New York, NY USA; 4 Executive Administration Florida, Cleveland Clinic Florida, Weston, FL USA; 5 Department of Colorectal Surgery, Lankenau Medical Center, Wynnewood, PA USA; 6 Division of Colon and Rectal Surgery, UMass Memorial Medical Center, Worcester, MA USA; 7 Division of Colorectal Surgery, Cedars-Sinai Medical Center, Los Angeles, CA USA; 8 Gastrointestinal and Minimally Invasive Surgical Division, The Oregon Clinic, Providence Cancer Center, Portland, OR USA; 9 Division of Colorectal Surgery, Department of Surgery, Kaiser Permanente Los Angeles Medical Center, Los Angeles, CA USA; 10 Division of Surgical Oncology, Department of Surgery, Princess Margaret Cancer Centre and University Health Network, Toronto, ON Canada; 11 Department of Colorectal Surgery, Mayo Clinic, Rochester, MN USA; 12 Department of Surgery, Cleveland Clinic, Cleveland, OH USA; 13 Division of Colorectal Surgery, Department of Surgery, New York-Presbyterian Weill Cornell Medical Center, New York, NY USA; 14 Department of Colon and Rectal Surgery, Advent Health Orlando, Orlando, FL USA; 15 Department of Population Health Sciences and Policy, Icahn School of Medicine at Mount Sinai Hospital, New York, NY USA; 16 Department of Colorectal Surgery, Ellen Leifer Shulman and Steven Shulman Digestive Disease Center, Cleveland Clinic Florida, Weston, FL USA; 17 Division of Colon and Rectal Surgery, Mount Sinai Hospital, New York, NY USA

**Keywords:** Rectal cancer, Transanal total mesorectal excision, TME grade, Circumferential radial margin, Conversion, Anastomotic complication, Stoma-free rate

## Abstract

**Background:**

Transanal TME (taTME) combines abdominal and transanal dissection to facilitate sphincter preservation in patients with low rectal tumors. Few phase II/III trials report long-term oncologic and functional results. We report early results from a North American prospective multicenter phase II trial of taTME (NCT03144765).

**Methods:**

100 patients with stage I–III rectal adenocarcinoma located ≤ 10 cm from the anal verge (AV) were enrolled across 11 centers. Primary and secondary endpoints were TME quality, pathologic outcomes, 30-day and 90-day outcomes, and stoma closure rate. Univariable regression analysis was performed to assess risk factors for incomplete TME and anastomotic complications.

**Results:**

Between September 2017 and April 2022, 70 males and 30 females with median age of 58 (IQR 49–62) years and BMI 27.8 (IQR 23.9–31.8) kg/m^2^ underwent 2-team taTME for tumors located a median 5.8 (IQR 4.5–7.0) cm from the AV. Neoadjuvant radiotherapy was completed in 69%. Intersphincteric resection was performed in 36% and all patients were diverted. Intraoperative complications occurred in 8% including 3 organ injuries, 2 abdominal and 1 transanal conversion. The 30-day and 90-day morbidity rates were 49% (Clavien–Dindo (CD) ≥ 3 in 28.6%) and 56% (CD ≥ 3 in 30.4% including 1 mortality), respectively. Anastomotic complications were reported in 18% including 10% diagnosed within 30 days. Higher anastomotic risk was noted among males (*p* = 0.05). At a median follow-up of 5 (IQR 3.1–7.4) months, 98% of stomas were closed. TME grade was complete or near complete in 90%, with positive margins in 2 cases (3%). Risk factors for incomplete TME were ASA ≥ 3 (*p* = 0.01), increased time between NRT and surgery (*p* = 0.03), and higher operative blood loss (*p* = 0.003).

**Conclusion:**

When performed at expert centers, 2-team taTME in patients with low rectal tumors is safe with low conversion rates and high stoma closure rate. Mid-term results will further evaluate oncologic and functional outcomes.

**Graphical abstract:**

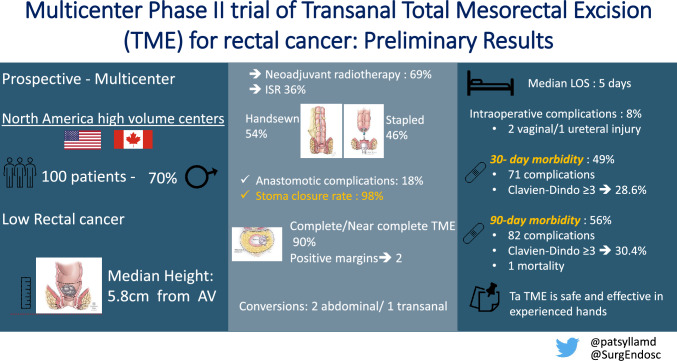

**Supplementary Information:**

The online version contains supplementary material available at 10.1007/s00464-023-10266-9.

In conjunction with major advancements in neoadjuvant treatment strategies, worldwide adoption of total mesorectal excision (TME) technique in the curative resection of locally advanced rectal cancer over the past 3 decades has reduced 5-year local recurrence (LR) rates to 3.3–5.7% in large contemporary series [1–5]. Laparoscopy was a major advance in innovation for the surgical treatment of rectal cancer, although acceptance and adoption have been slowed by long learning curves and lingering concerns regarding inferior rate of circumferential resection margins (CRM) relative to open TME. By providing minimally invasive access to the pelvis, laparoscopic and robotic-assisted TME have improved short-term postoperative recovery without significantly impacting oncologic or functional outcomes relative to open proctectomy. As reflected in recent comparative trials, persistently high abdominoperineal resection (APR), CRM positive and conversional rates reported in male and obese patients relate to tumor location ≤ 6 cm from the anal verge [1, 6, 7]. Transanal TME (taTME) evolved from NOTES (Natural Orifice Transluminal Endoscopic Surgery) and is most commonly performed in a hybrid fashion in combination with abdominal laparoscopic assistance. Since the report of the first case in 2010, rapid adoption of taTME worldwide reflects the perceived benefits of direct in-line access to the distal rectum, augmented exposure, and navigation provided by pneumodistention and image guidance through multiport transanal endoscopic platforms in facilitating these complex procedures [8–11]. Large retrospective institutional and multicenter cohort studies have reported procedural and short-term oncologic results commensurate with those from laparoscopic trials, with notably lower conversion rates and higher rates of sphincter preservations [12, 13], but also a non-negligible incidence of procedure-specific adverse events including urethral injury and CO2 embolism [14–17]. Several national cancer audits, cohort studies, and the first recently published randomized trial of laparoscopic vs taTME have reported 3-year local recurrence rates ranging from 1.9% to 6.2% [18–27]. However, results from the 2019 Norwegian audit of 157 consecutive taTME cases have led several centers to abandon taTME, and recommend against adoption of the technique outside of centers with strict training pathways [28]. This posture was based on the 7.6% observed overall local recurrence rate, with 11.6 vs 2.4% estimated 2-year LR rate in the taTME vs non-taTME groups, in a cohort from the Norwegian Colorectal Cancer Registry, including a 5% reported incidence of multifocal pelvic side wall recurrences [28]. While variability in case selection, training and surgical technique are known to contribute to unfavorable outcomes during the early adoption phase of any new surgical procedure, ongoing randomized control trials (RCTs) will further clarify the impact of TME technique on oncologic results [29, 30]. In the US, based on the trend among experienced surgeons to favor taTME for tumors located in the distal rectum, a prospective phase II trial was designed to evaluate the safety, oncologic and functional results of taTME in a cohort of 100 patients with stage I–III rectal cancer when performed by experienced taTME surgeons beyond their learning curve. In this manuscript, postoperative morbidity at 30 and 90 days including outcomes of anastomotic complications, stoma closure rate, and pathologic outcomes are reported.

## Methods

### Study design

This is a phase II prospective multicenter trial conducted across 11 experienced taTME centers in the US and Canada. The primary endpoint was the rate of complete and near-complete mesorectal excision achieved with taTME, and secondary endpoints included detailed pathology assessment, 30 and 90-day complications, functional outcomes post-stoma reversal, and 3-year oncologic outcomes. The protocol was approved at each participating site IRB, and written informed consent was obtained from all study participants prior to enrollment (Protocol, Online Appendix 1). The trial was registered on clinicaltrial.gov (NCT03144765). The manuscript conforms to the Strengthening the Reporting of Observational Studies in Epidemiology (STROBE) guidelines [31].

Surgeon eligibility requirements included performance of ≥ 20 sphincter-preserving low anterior resections (LAR) including ≥ 5 taTME cases in the preceding 12 months and ≥ 20 transanal endoscopic resections by transanal surgeons in the preceding 24 months. Participating surgeons had been performing taTME procedures for a minimum of 3 years, and were required to submit recent unedited transanal video recordings of 2 cases, including one in a male patient, along with pathology reports and photographs of gross TME specimens to confirm technical proficiency. Study sites were required to follow National Accreditation Program in Rectal Cancer (NAPRC) standards with respect to staging and treatment protocols, multidisciplinary tumor board (MDT) review and consensus treatment planning of all rectal cancer patients [32].

Patients > 18 years old, with clinically staged I–III biopsy-confirmed adenocarcinoma, located ≤ 10 cm from the anal verge, who were candidates for minimally invasive curative sphincter-preserving low anterior resection with tumor-specific (TSME) or TME, were screened for study enrollment. Tumor height and relationship to the anal sphincters was based on digital rectal exam (DRE), and/or rigid proctosigmoidoscopy, and confirmed on staging MRI and operative reports. Staging was completed with CT scans of the chest, abdomen and pelvis and rectal cancer protocol pelvic MRI. Neoadjuvant and adjuvant treatment protocols were based on MDT consensus recommendations and included short-course radiotherapy (SCRT), long-course chemoradiotherapy (CRT), total neoadjuvant therapy (TNT) or chemotherapy only. Chemotherapy regimens included Capecitabine, 5-FU, FOLFOX, or Capecitabine in combination with Oxaliplatin. Oncologic surveillance scheduled followed NCCN guidelines. Clinical assessment was performed at 2 and 4–6 weeks, 5–7 months and 11–13 months postoperative visits in order to collect adverse events. Patients completed baseline preoperative functional questionnaires at least once prior to surgery, and at two postoperative time points post-stoma reversal, when applicable.

**Fig. 1:**
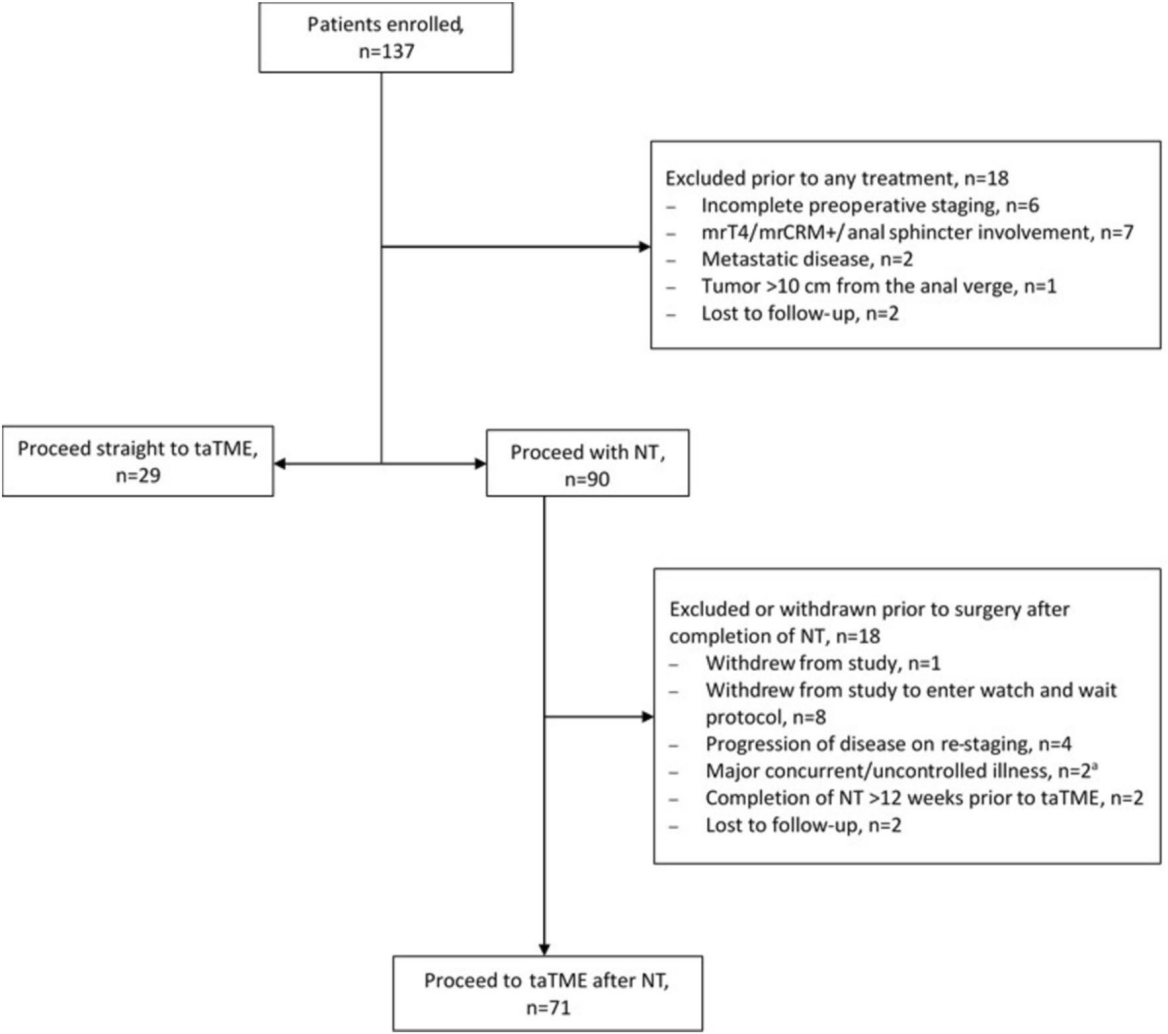
Patient enrollment flowchart. NT, neoadjuvant treatment; mrT4/mrCRM + , predicted T4 or positive circumferential margin on staging pelvic MRI; ^a^1 patient with neurological decline after NT, 1 patient with concurrent urothelial malignancy

Enrollment occurred either prior to neoadjuvant treatment or prior to taTME (Fig. 1). All enrolled patients were reviewed at MDT at least once prior to taTME procedures and the decision to re-stage post-neoadjuvant treatment was up to site protocol and preferences. Exclusion criteria included metastatic or synchronous disease, severe intercurrent illness, T4 tumors and tumors with positive predicted circumferential margin (CRM) and/or involvement of anal sphincters on staging or post-treatment pelvic MRI, fecal incontinence, prior colorectal cancer or rectal resection and inflammatory bowel disease. Patients were also excluded if taTME was delayed more than 12 weeks post-completion of neoadjuvant treatment, and if they were unable to compete the functional questionnaire in English.

### Study procedures and data collection

Preoperative bowel preparation and venous thrombosis prophylaxis were administered based on sites’ local protocols. Enhanced recovery protocols were used when possible with intraoperative anesthetic regimens including local, regional or intrathecal anesthesia. Surgeons were required to perform all taTME procedures with a 2-team approach using sequential or simultaneous abdominal and transanal dissection, and to video record all transanal procedures (Protocol, Online Appendix 1). Abdominal minimally invasive approach, distal vs proximal extent of abdominal vs transanal, and extent of anterior vs posterior TME dissection was left up to surgeons’ preferences. Typically, the anterior TME dissection was carried down to the anterior peritoneal reflection or mid-vagina/prostate, while posterior TME dissection is typically extended to the S1 or S2 level. For transanal procedures, the rectal lumen was occluded below the tumor with a single or double purse-string suture with air-tight closure prior to initiating taTME dissection. Alternatively, purse-string closure followed initial mucosectomy or partial intersphincteric resection, when performed. The choice of transanal endoscopic platform, splenic flexure mobilization, specimen extraction site, intraoperative bowel perfusion assessment by fluorescence angiography, anastomotic configuration, fecal diversion and pelvic drain placement were left to the surgeon’s preference and recorded. Gross specimens were photographed, inked and processed according to standard TME protocols [33–35].

Patient and tumor characteristics including demographics, comorbidities, tumor height, radiologic staging, neoadjuvant treatment received, and time interval between end of treatment and surgery were collected. Procedural details, intraoperative events, and length of hospital stay were recorded, in addition to postoperative complications reported during follow-up clinical visits, which were graded using Clavien–Dindo classification [36]. Anastomotic complications were reported based on clinical (by direct visualization or digital palpation, inspection of drain output), radiologic (CT scan, contrast enema) or endoscopic (sigmoidoscopy or colonoscopy) evidence of anastomotic leakage, ischemia, dehiscence or defect, peri-anastomotic cavity, sinus or fistula, as well as significant strictures (requiring more than single finger dilatation). Anastomotic leak (AL) refers to the early presentation of an anastomotic complication (identified within 30 days of surgery) and was reported based on clinical, radiologic, or endoscopic finding of an anastomotic defect, dehiscence, ischemia, and/or finding of pelvic abscess or phlegmon immediately adjacent to the anastomosis. Time to diagnosis, diagnostic modality, and interventions used to manage anastomotic complications were collected until ileostomy reversal or conversion to permanent stoma. Anastomotic complications were graded according to CD classification as well as the International Study Group of Rectal Cancer (ISGRC) classification [36, 37]. The protocol was amended in August 2018 to include collection of C-reactive protein level (CRP) on postoperative day (POD1-4), when applicable (Protocol, Online Appendix 1) [38].

Pathological assessment of specimens was collected from synoptic reports and included grade of completeness of the mesorectum, TNM stage, and assessment of circumferential radial margins, and proximal and distal margins [34]. Assignment of a grade of complete mesorectum required demonstration of a smooth surface of mesorectal fascia with the entire mesorectal envelope present and no defect deeper than 5 mm, while a near-complete mesorectum demonstrated an intact mesorectal envelope except for small irregularities and/or defects greater than 5 mm but not extending to the muscularis propria. In contrast, an incomplete mesorectum demonstrated deeper defects in the mesorectum with exposed muscularis propria and/or very irregular circumference. Positive proximal, distal, and circumferential resection margins were defined as viable tumor located ≤ 1 mm from the respective margin.

Urologic, sexual, and defecatory function was assessed at least once preoperatively and at 2 postoperative time points, 3–4 months and 9–12 months post-stoma reversal. Validated questionnaires were used, including COREFO (Colorectal Functional Outcome) [39], FIQL (Fecal Incontinence Quality of Life questionnaires) [40, 41], female sexual function Index (FSFI) [42], IIEF (International Index of Erectile Function questionnaires [43], and IPSS (International Prostate Symptom Score) [44]. Oncologic outcomes data will be collected up to 5 years following surgery including local and distant recurrence, disease-free (DFS) and overall survival (OS). Data entry was completed on REDCAp, a research electronic data capture platform, while photographs of TME specimens and unedited taTME videos were uploaded on an encrypted HIPAA compliant, password-protected Mount Sinai Hospital server.

### Surgical and pathological quality control

Site initiation included review of standard TME protocols by site pathologists and pathology staff through webinars and a standard of operations (SOP) manual [33–35]. Quality assurance consisted of continuous remote monitoring to ensure accurate and timely data entry and reporting of adverse events and compliance with study procedures. Study sites underwent one on-site monitoring visit after the first 1–5 procedures to ensure compliance with study protocols. Quality assurance for pathology procedures included monitoring of compliance with standard TME protocol for specimen handling, processing and evaluation, central review of all TME gross photographs by central blinded pathologists to establish concordance in TME grade, and monitoring of the incidence of incomplete TME and/or positive resection margins. Quality improvement measures included (1) retraining of pathology staff on specimen handling and processing, (2) reviewing and reconciliation of major discordances in TME grading between study site and blinded central pathologists, (3) a stopping rule for sites with 2 cases of incomplete TME and/or positive surgical margins to allow for assessment of the adequacy of dissection based on blinded video review by an external taTME expert, with implementation of a corrective action plan. Interim analysis by a study data safety monitoring board was completed after the first 50 patients were enrolled.

### Sample size and statistical analysis

The primary objective of this study was to determine whether taTME was non-inferior to standard LAR in terms of the proportion of subjects that achieve complete or near-complete mesorectal excision. With a sample size of 100, the one-sided binomial test will reject the null hypothesis that the success rate is ≤ 80% if the study procedure leads to efficacy of the total mesorectal excision for 87 or more subjects. This design achieves a power of 87% using one-sided binomial test for non-inferiority with 5% type 1 error assuming the true success rate is 90%. Patient demographic, disease-related, treatment-related, operative characteristics as well as pathologic outcomes and 90-day surgical complications were summarized for continuous variables as median and first and third quartiles, Q1–Q3, and for categorical variables as counts and percentages. Distributions of categorical variables were compared among group using the *χ*2 or Fisher’s exact test when appropriate. Univariable log-binomial regression was used to investigate associations between some of the aforementioned characteristics, identified through an extensive literature search as potential risk factors, and incomplete pathologic TME grade, reporting relative risks (RRs), corresponding 95% confidence intervals (CIs), and p-values. RRs were selected instead of odds ratios via logistic regression because the latter tends to overestimate the strength of the association when the incidence of the outcome is 10% or more, as it is in this study. Times from surgery to stoma closure were analyzed using the Kaplan–Meier (KM) method. Comparison of stoma closure KM distributions was made between groups with the log-rank test. Eighty-two patients were censored for anastomotic leak at the time of their ileostomy reversal or permanent colostomy creation. Two patients were censored for stoma closure at their permanent colostomy creation. Duration of follow-up for postoperative complications was calculated as the maximum of 90 days and days to ileostomy reversal or permanent colostomy creation, except for in one patient whose follow-up was only 70 days due to death. Univariable Cox proportional hazards regression was used to investigate associations between characteristics identified through literature search as potential risk factors for anastomotic leak and/or stoma closure, reporting hazard ratios (HRs), corresponding 95% confidence intervals (CIs), and *p* values. All statistical analyses were performed using SAS statistical software (version 9.4; SAS Institute). Hypothesis testing was conducted at the 5% level of significance. However, *p* values less than 10% were considered borderline significant in exploratory regression analyses.

## Results

### Accrual and Demographics

**Fig. 2:**
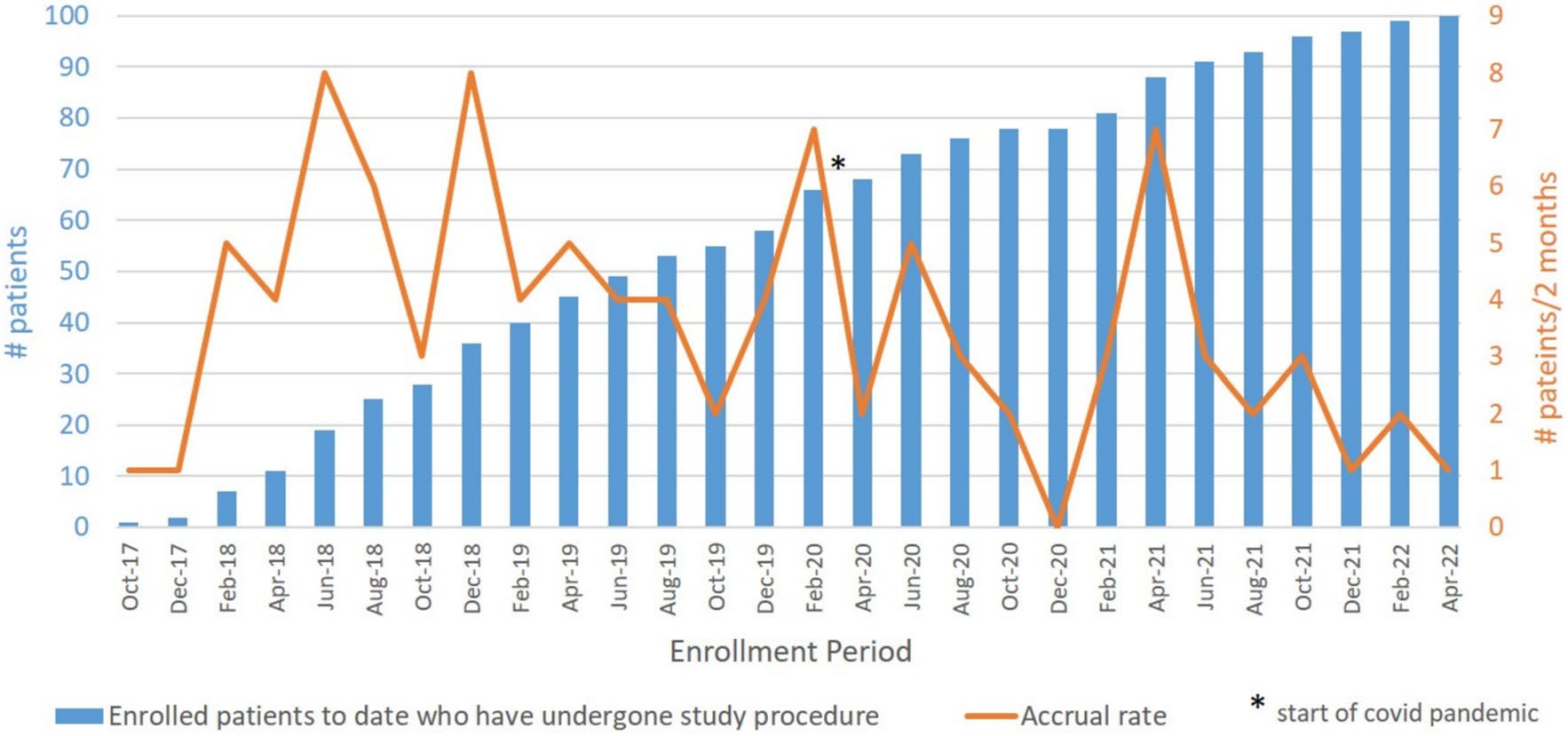
Trial Accrual, bar graph with cumulative number of patients accrued on the taTME trial at 2-month intervals and line graph demonstrating the rate of accrual per 2 months

Between September 2017 and April 2022, 137 patients were enrolled across 11 sites in the United States and Canada. There were 37 screen failures due to exclusion criteria, withdrawal of consent or loss to follow-up. In 8 cases where clinical complete response was achieved post-neoadjuvant therapy, patients withdrew from the study to enter watch and wait protocols (Fig. 1). In total, 100 patients underwent taTME procedures with total accrual ranging from 2 to 17 subjects per study site (Fig. 2). Below target patient accrual at 5 study sites was due to site PI relocation, screen failures, and COVID-related interruptions. Between March 2020 and February 2021, COVID-related trial disruptions resulted in delays in accrual, in-person follow-up intervals, surveillance, and ileostomy reversal (Fig. 2). Among 100 taTME patients, 70 were male vs 30 female, 22% self-identified as other than Caucasian, median age was 58 (IQR 49–62), and median BMI was 27.8 (IQR 23.9–31.8) with 33% of patients with BMI ≥ 30 (Table 1).

**Table 1:** Demographic and clinical characteristics

	*n* = 100 patients
Male	70 (70.0%)
Age at index surgery, median [IQR]	58 [49, 62]
Race	
Caucasian	78 (78.0%)
Asian	8 (8.0%)
Black	5 (5.0%)
Hispanic	5 (5.0%)
Other^a^	4 (4.0%)
ASA score	
1/2	60 (60.0%)
3	40 (40.0%)
ECOG score	
0	90 (90.0%)
1	10 (10.0%)
BMI (kg/m2), median [IQR]	27.8 [23.9, 31.8]
< 30	67 (67.0%)
≥ 30	33 (33.0%)
Smokers	7 (7.0%)
Comorbidities	37 (37.0%)
Hypertension	33 (33.0%)
Diabetes	11 (11.0%)
Liver transplant	1 (1.0%)
Previous unrelated abdominal surgery	10 (10%)
Clinical AJCC stage	
I	35 (35.0%)
II	22 (22.0%)
III	43 (43.0%)
Tumor distance from anal verge (cm), median [IQR]	5.8 [4.5, 7.0]
≤ 6 cm	67 (67.0%)
> 6 cm	33 (33.0%)
Clinical tumor stage	
cT1^b^	5 (5.0%)
cT1^b^/2	9 (9.0%)
cT2	28 (28.0%)
cT2/3a	1 (1.0%)
cT3	57 (57.0%)
Clinical node stage	
cN0	57 (57.0%)
cN1	31 (31.0%)
cN2	12 (12.0%)
CEA at diagnosis (ng/mL), median [IQR]	1.9 [1.1, 4.1]
Neoadjuvant treatment	71 (71.0%)
Long course CRT	51 (51.0%)
Total neoadjuvant therapy	17 (17.0%)
Chemotherapy alone	2 (2.0%)
Short course RT	1 (1.0%)
Restaging MRI post CRT	57 (57.0%)
Time from end of RT to index surgery, days, median [IQR]	76 [68–82]

^a^Includes Guyanese, Native American, Middle Eastern, and Pacific Islander

^b^Completion TME was indicated in 8 patients following local excision due to the presence of high-risk histopathologic features

*IQR* interquartile range; *ASA* American Society of Anesthesiologists; *BMI* body mass index; *AJCC* American Joint Committee on Cancer; *CEA* carcinoembryonic antigen; *CRT* chemoradiation therapy, *RT* radiation therapy; *MRI* magnetic resonance imaging

### Tumor characteristics

Median tumor height was 5.8 cm from the anal verge (IQR 4.5–7.0). Tumors were preoperatively staged as clinical stage I (35%), stage II (22%), or stage III (43%). Among stage I tumors, 8 cT1N0 or cT1/2N0 patients underwent completion taTME after local excision and uncovered high-risk histopathological features (poor differentiation, lymphovascular invasion, high tumor budding, and positive margin) warranting radical resection based on MDT consensus recommendations (Table 1). Neoadjuvant treatment was completed in 71% of patients, including 64 of the 65 stage II/III tumors and 7 stage I (T2N0) tumors. A significantly higher proportion of tumors located ≤ 6 cm from the AV were treated with neoadjuvant therapy than tumors > 6 cm (76.4% vs 25.4%, p = 0.011). Neoadjuvant regimens included long-course CRT in 51/71 (71.8%), TNT in 17/71 (23.9%), chemotherapy only in 2/71 (2.8%), and SCRT in 1/71 (1.4%). TNT strategy was employed by 6 of 11 sites during the study period, with 5 patients between 2017 and 2019 compared with 12 patients between 2021 and 2022 receiving TNT. Among 11 stage III, 5 stage II, and 1 stage I treated with TNT, 64.7% vs 35.3% received induction vs consolidation TNT. Overall, 80.3% (57/71) of tumors were re-staged post-neoadjuvant treatment. Median time between the end of neoadjuvant radiation and taTME was 76 (IQR 68–82) days (Table 1).

### Surgical procedures

All procedures were completed using a 2-team approach with abdominal dissection performed by multiport (94%), robotic (4%), single port (1%), or hand-assisted laparoscopy (1%) with splenic flexure mobilization performed in 94 cases. Concurrent surgical procedures were performed in 8 patients (Table 2). Transanal platforms used included rigid (26%) or disposable endoscopic platforms (74%). In 38 cases where tumors were located a median of 4 cm from the AV, transanal mucosectomy (2) or partial intersphincteric resection (ISR, 36) was performed in combination with taTME. There were 11 intraoperative complications reported among 8 patients including 2 abdominal conversions to open surgery (ischemic colonic conduit, bleeding), 1 transanal conversion to abdominal robotic dissection (failure to progress), 1 suspected case of CO_2_ embolus, 2 vaginal injuries during transanal dissection, and 1 colotomy, 1 enterotomy, and 1 ureteral transection during abdominal dissection, all identified and repaired. Perfusion assessment of the colonic conduit and/or colorectal anastomosis was performed by fluorescence angiography using indocyanine green (ICG) in 55% (Table 2). Inadequate perfusion was observed in 9 cases and prompted intervention in 8 cases (change in proximal resection margin with additional mobilization, suture removal). TME specimens were extracted transanally in 52%, with handsewn coloanal anastomosis performed in 54%. Anastomotic reconstruction was completed end-end (89%), side-to-end (6%), or with coloanal J pouch (5%). All patients were diverted with a loop ileostomy and pelvic drains were placed in 80%. Median total operative time was 311.5 (IQR 265.5–380.5) minutes and median transanal dissection time was 101 (IQR 74.5–174) minutes. Median estimated blood loss was 100 (IQR 50–250) ml and blood transfusion was not required.

**Table 2:** Intraoperative details

	*n* = 100 patients
Operative time (minutes), median [IQR]	
Total	311.5 [265.5, 380.5]
Transanal	101 [74.5, 174]
EBL (mL), median [IQR]	100 [50, 250]
Intraoperative analgesia^a^	
Local anesthesia	43 (43.0%)
TAP Block	42 (42.0%)
Intrathecal^b^	9 (9.0%)
Concurrent procedure	8 (8.0%)
Salpingo-oophorectomy	5 (5.0%)
Umbilical hernia repair	2 (2.0%)
Cholecystectomy	1 (1.0%)
Intraoperative complications	8 (8.0%)
Conversion	3 (3.0%)
Vaginotomy	2 (2.0%)
Suspected CO_2_ embolus	1 (1.0%)
Bleeding	1 (1.0%)
Colotomy	1 (1.0%)
Ischemic conduit	1 (1.0%)
Ureteral injury	1 (1.0%)
Enterotomy	1 (1.0%)
Total number of complications	11
Approach to abdominal procedure	
Multiport laparoscopy	94 (94.0%)
Robotic-assisted laparoscopy	4 (4.0%)
Single incision laparoscopy	1 (1.0%)
Hand-assisted laparoscopy	1 (1.0%)
Splenic flexure mobilization	94 (94.0%)
Abdominal converted to open	2 (2.0%)
Transanal platform used	
GelPOINT® path	74 (74.0%)
TEO®	15 (15.0%)
TEM	11 (11.0%)
Partial ISR or mucosectomy	38 (38%)
Partial ISR	36 (36.0%)
Mucosectomy	2 (2.0%)
Transanal insufflator	
Airseal®	92 (92.0%)
Unspecified high-pressure insufflator	5 (5.0%)
Unspecified standard insufflator	3 (3.0%)
Transanal conversion to abdominal	1 (1.0%)
Specimen extraction site	
Transanal	52 (52.0%)
Pfannenstiel incision	26 (26.0%)
Periumbilical incision	13 (13.0%)
Lower midline incision	5 (5.0%)
Ileostomy site	3 (3.0%)
Lower left quadrant	1 (1.0%)
FA perfusion assessment with ICG^c^	55 (55%)
FA perfusion assessment outcome, n (%) of those with FA	
Adequate	46 (83.6%)
Inadequate	9 (16.4%)
Action taken, n (%) of cases of inadequate perfusion	
Yes	8 (88.9%)
No	1 (11.1%)
Anastomotic technique	
Handsewn	54 (54.0%)
Stapled	46 (46.0%)
Stapler size, *n *(%) of those with stapled anastomosis	
28	15 (32.6%)
29	14 (30.4%)
31	11 (23.9%)
33	6 (13.0%)
Anastomotic reconstruction	
End-to-end	89 (89.0%)
Side-to-end	6 (6.0%)
Coloanal J pouch	5 (5.0%)
Diverting loop ileostomy performed	100 (100.0%)
Pelvic drain placed	80 (80.0%)

^a^As single agent or in combination;

^b^Single shot of intrathecal morphine given at start of case;

^c^Systems used for FA include PINPOINT (Novadaq Technologies ULC, Burnaby, BC, Canada); 1588 AIM System, 1688 AIM system, SPY-PHI (Stryker Corp, Kalamazoo, MI); NIR/ICG (Karl Storz Endoscopy America Inc, Auburn, MA); Firefly™ (Intuitive Surgical, Sunnyvale, CA)

*EBL* estimate blood loss; *TAP* transabdominal plane; GelPOINT Path (Applied Medical, Rancho Santo Margarita, CA); TEO®, transanal endoscopic operations (Karl Storz Endoscopy, Tuttlingen, Germany); *TEM* (Richard Wolf, Knittlingen, Germany), transanal endoscopic microsurgery; *ISR* intersphincteric resection; Airseal® (CONMED Corporation, Utica, NY, USA); *FA* fluorescence angiography; *ICG* Indocyanine Green; *NIR* near-infrared fluorescence

### 30- and 90-day postoperative outcomes

**Fig. 3:**
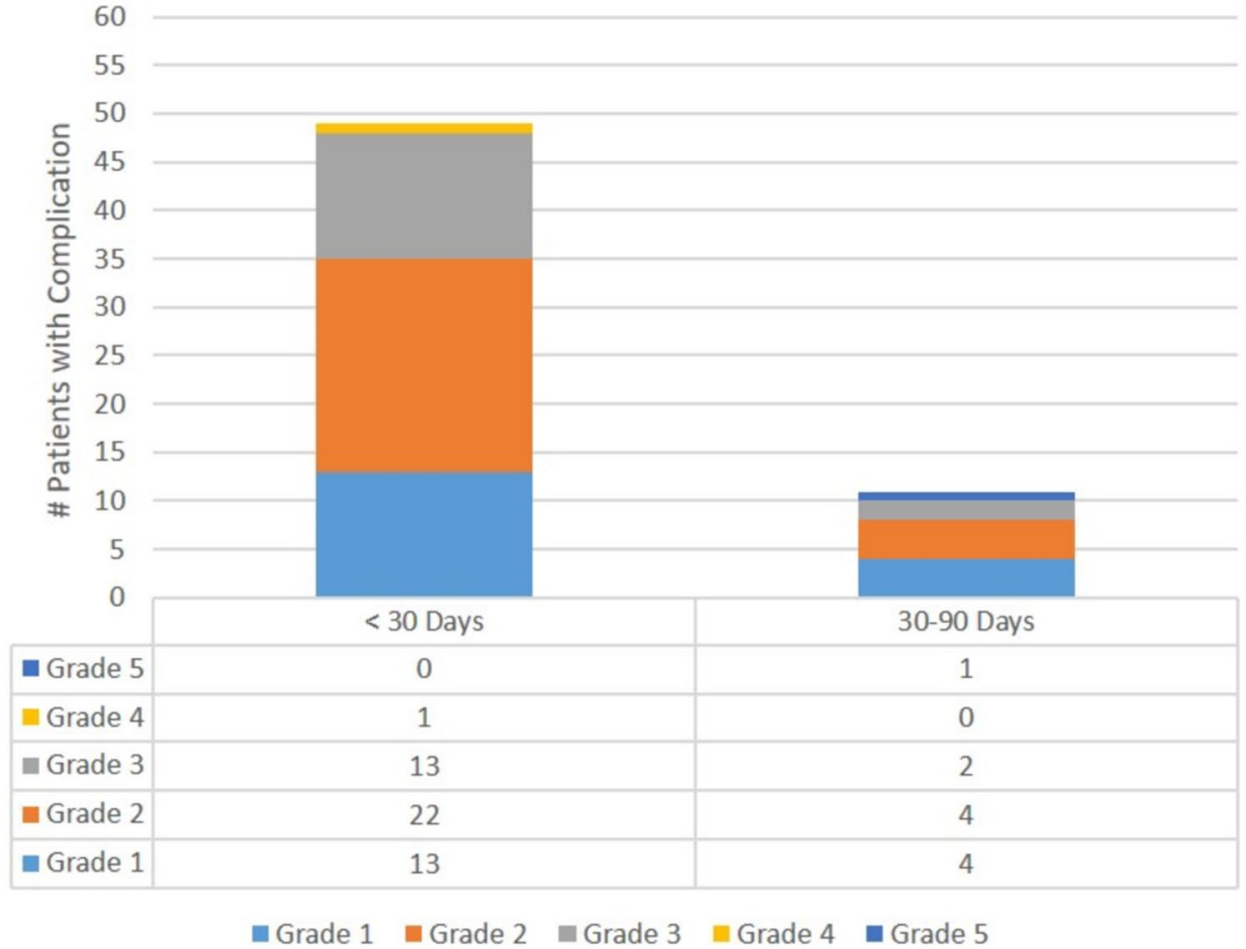
Postoperative complications 30- and 90-day complications with highest CD grade per patient

Median length of hospital stay was 5 (IQR 4–8) days. In total, 71 complications were reported in 49 patients within 30 days of surgery (CD3/4 in 28.6%) with a 17% readmission rate (Table 3, Fig. 3). Postoperative ileus with dehydration with or without high stoma output accounted for 35.2% (25/71) of 30-day complications and 47.1% (8/17) of readmissions. Ileus and high stoma output were associated with transient acute kidney injury in 4 (16%) patients. Urinary retention was reported in 19% with a 21.4 vs 13.3% incidence in male vs female patients (*p* = 0.344). Urinary retention was managed with medication in 68% of patients and resulted in prolonged bladder catheterization beyond discharge in 21% of patients. Other morbidities included anastomotic (10%), infectious (6%), and thrombotic (3%) complications, small bowel obstruction (SBO, 4%), pancreatic fistula (1%), bleeding duodenal ulcer (1%), myocardial infarction (1%), and intractable rectal pain (1%). Reoperation was required in 11/49 patients (22.4%), for anastomotic complications (*n* = 8), SBO (*n* = 2) and refractory rectal pain (*n* = 1). Between postoperative day 30 and 90, 11 new complications were recorded in 11 patients (CD1/2 in 72.7%, CD3/5 in 27.3%) including 1 death from suicide at POD70, 5 new anastomotic complications, 1 ileus with dehydration, 1 SBO, 1 DVT, and 1 stomal and 2 mucosal prolapse. Reoperation was required in 2/11 (18.2%) patients for 1 anastomotic complication and 1 stoma prolapse (Fig. 3).

**Table 3:** Postoperative outcomes

	*n* = 100 patients
Length of stay (days), median [IQR]	5 [4, 8]
Length of follow-up^a^ (months), median [min, Q1, Q3, max]	5 [2.3, 3, 7, 23]
Postoperative complications (< 30 Days)^b^	49 (49.0%)
Ileus ± stoma-related dehydration^c^	25 (25.0%)
With prolonged hospitalization^d^	10 (10.0%)
Urinary retention^e^	19 (19.0%)
Anastomotic leaks/complications	10 (10.0%)
SBO	4 (4.0%)
UTI	3 (3.0%)
SSI	2 (2.0%)
DVT/PMVT	2 (2.0%)
Pulmonary embolism	1 (1.0%)
Pancreatic leak	1 (1.0%)
C. diff infection	1 (1.0%)
Myocardial infarction	1 (1.0%)
Bleeding duodenal ulcer	1 (1.0%)
Rectal pain	1 (1.0%)
Total number of 30-day complications	71
CD classification < 30 days^f^	
CD1/2	35 (35.0%)
CD3/4	14 (14.0%)
30-day readmission	17 (17.0%)
30-day reoperation	11 (11.0%)
Postoperative complications (30–90 days)	11 (11.0%)
Anastomotic complications	5 (5.0%)
Ileus ± stoma-related dehydration^c^	1 (1.0%)
SBO	1 (1.0%)
DVT	1 (1.0%)
Ileostomy prolapse	1 (1.0%)
Mucosal prolapse	1 (1.0%)
Death by suicide	1 (1.0%)
Total number of 30–90-day complications	11
CD classification 30–90 days	
CD1/2	8 (8.0%)
CD3/5	3 (3.0%)
Total patients with postoperative complications < 90 days^g^	56 (56.0%)
Total number of < 90-day complications	82
CD classification < 90 days	
CD1/2	39 (39.0%)
CD ≥ 3	17 (17.0%)
POD3 CRP ≥ 145, among patients with CRP drawn POD3	15/67 (22.4%)

^a^Duration of follow-up used for this analysis was the longer of either the 90-day postoperative complication monitoring period or days from surgery to ileostomy reversal/permanent colostomy creation; exception for 1 patient, whose follow-up was only 70 days due to death

^b^Some patients with multiple complications

^c^With dehydration ± acute kidney injury ± high ileostomy output

^d^Defined as LOS ≥ 8 days

^e^Foley reinsertion and/or medical treatment

^f^Highest grade in those with multiple complications

^g^Accounts for those with complications occurring at both < 30 days and 30–90 days

*CD* Clavien-Dindo; *SBO* small bowel obstruction; *DVT* deep vein thrombosis; *PMVT* portal-mesenteric vein thrombosis; *SSI* surgical site infection; *SBO* small bowel obstruction; *CRP* c-reactive protein; *LOS* length of stay

### Anastomotic complications and stoma reversal

**Fig. 4:**
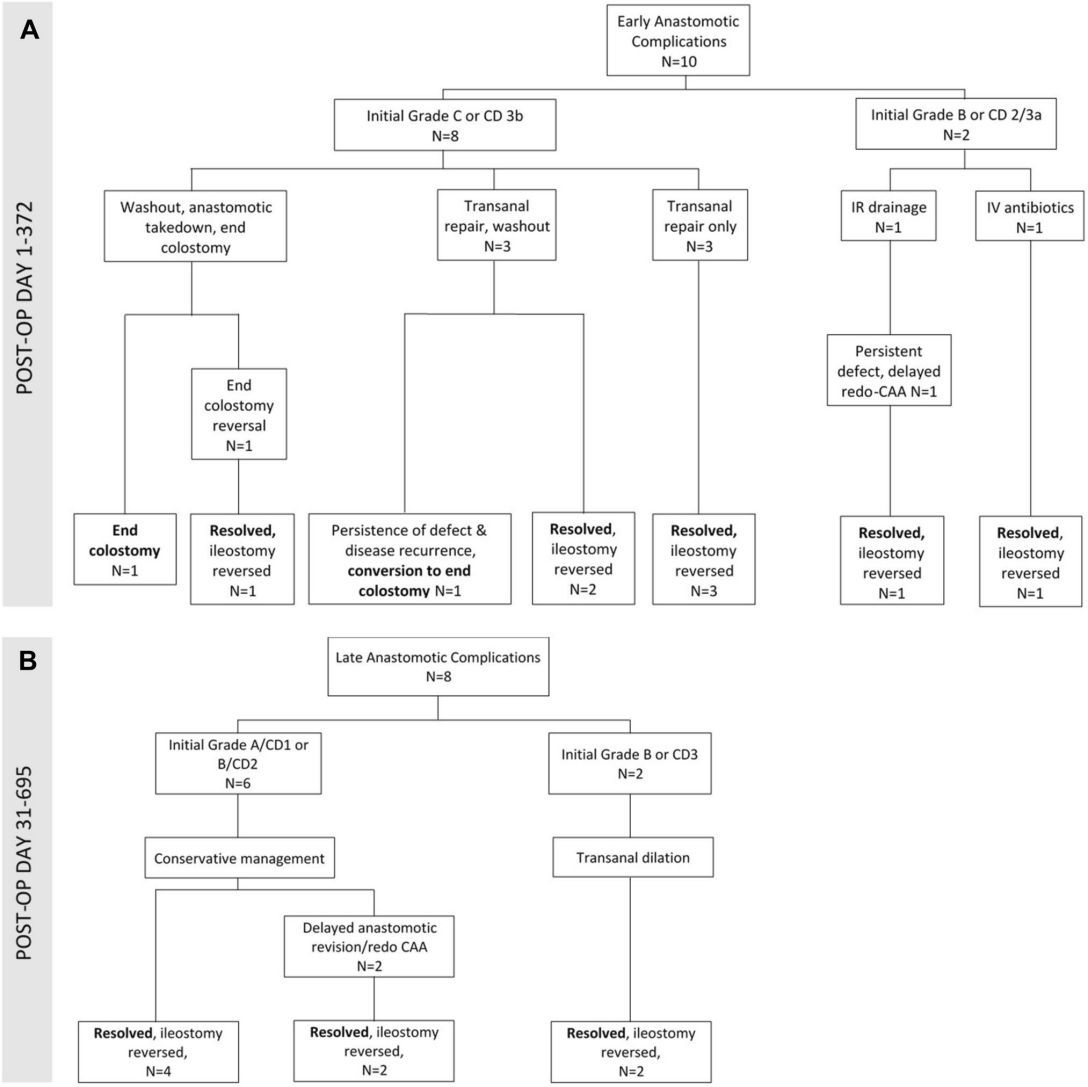
A. Management and outcomes of early anastomotic complications (< 30 days); **B.** Management and outcomes of late anastomotic complications (> 30 days). See Table 5 for details regarding each case. *POD* postoperative day; *CD* Clavien-Dindo; *IR* interventional radiology; *IV* intravenous; *CAA* coloanal anastomosis

At a median follow-up of 5 (IQR 3–7, range 2.3–23) months, 18 anastomotic complications were reported among 17 males and 1 female (*p* = 0.012), and included 10 early (diagnosed POD0-POD29), 5 late (POD30-POD90), and 3 very late anastomotic complications (> 90 days). Among 10 early anastomotic complications, 8 underwent early re-intervention and 2 were managed with percutaneous drainage and/or antibiotics (Tables 4 and 5, Fig. 4a). Among the 8 that underwent early re-intervention, 5 presented with pelvic sepsis requiring urgent pelvic washout with either transanal repair of anastomotic dehiscence (*n* = 3), or anastomotic takedown with end colostomy (*n* = 2), while 3 relatively asymptomatic patients underwent early transanal closure of small anastomotic defects. Overall, resolution of 6 anastomotic complications was achieved with 0–1 reinterventions within 30 days followed by ileostomy reversal between POD86 and POD372 (Table 5). Delayed anastomotic revision with redo-coloanal anastomosis was required in 1 patient, and Hartman’s reversal was performed in another patient who underwent early anastomotic takedown with end colostomy. Both patients underwent delayed ileostomy closure between POD271 and POD304. Two patients were permanently diverted including one who underwent anastomotic takedown due to conduit ischemia, and another who was converted to a permanent colostomy due to due to a refractory anastomotic defect later complicated by peritoneal carcinomatosis (Fig. 4a and b, Table 5).

**Table 4:** Anastomotic complications and stoma outcomes

	*n* = 100 patients
Early, < 30 days	10 (10.0%)
Late, 30–90 days	5 (5.0%)
Late, > 90 days	3 (3.0%)
CD grade^a^	n = 18
1	3 (16.7%)
2	2 (11.1%)
3	13 (72.2%)
3a	1 (5.6%)
3b	12 (66.7%)
ISGRC grade^a^	n = 18
A- no specific treatment	3 (16.7%)
B- treatment other than laparotomy	7 (38.9%)
C- laparotomy	8 (44.4%)

	All patients *n* = 100	No anastomotic complications *n* = 82	Anastomotic complications *n* = 18	*p* value
Stoma closure	98 (98.0%)	82 (100%)	16^b^ (88.9%)	
Time to closure (days), median [IQR]	154 [94–224.0]	140 [91.0–213.0]	226 [140–304]	0.0015^c^
Closure delayed > 150 days	49 (49.0%)	38 (46.3%)	11 (68.8%)	
Reason for delay^d^, in n (%) with delay				
Adjuvant treatment	42 (85.7%)	33 (86.8%)	9 (81.8%)	
Postoperative complication	16 (32.7%)	5 (15.0%)	11 (100%)	
Anastomotic complication	11 (22.4%)	0 (0.0%)	11 (100%)	
Other^e^	5 (10.2%)	5 (13.2%)	0 (0.0%)	
Unspecified	2 (4.1%)	4 (10.0%)	0 (0.0%)	

^a^Accounts for intervention required with highest CD or ISGRC grade required for the anastomotic complication

^b^one patient converted to permanent end colostomy due to persistent anastomotic complication *and* development of disease recurrence

^c^Log-rank p-value

^d^For some patients (*n* = 6), there are multiple reasons for stoma closure delays so does not sum to 100%

^e^Includes patient preference, COVID, frailty, and diagnostic work-up of lung lesion

*CD* Clavien-Dindo

**Table 5:** Details of anastomotic complications: diagnosis, management, and outcomes

Patient	0–30 days post-taTME	30–90 days post-taTME	> 90 days post-taTME	Stoma reversal
Early anastomotic complications (< 30 days)				
1	POD4^a^: reop for rising CRP, transanal suture closure of small anastomotic defect	POD74: resolved^b^		POD86
2	POD8: abdominal and pelvic abscess, reop with transanal repair of defect and abdominal drainage	Ongoing anastomotic cavity, no intervention	POD113: resolved	POD140
3	POD11: asymptomatic defect on rectal contrast imaging, transanal repair		POD105: resolved	POD130
4	POD4: fecal content in drain, AL, and pelvic abscess on CT with rectal contrast, reop with transanal repair of anastomotic dehiscence and abdominal washout POD11: no clinical improvement, persistent leak on CT with rectal contrast, 2^nd^ reop with additional transanal drainage	Ongoing pelvic abscess, no intervention	POD272: resolved	POD372
5	POD6: symptomatic pelvic abscess treated with IV antibiotics		POD169: resolved	POD201
6	POD19: reop for anal pain, transanal suture repair of small posterior anastomotic dehiscence	POD45: pelvic abscess treated with oral antibiotics	POD91: EUA, resolved	POD107
7	POD3: fever, CT with pelvic collection, conservative management POD25: readmission with pelvic abscess, IV antibiotics and IR drainage		POD109: endoscopy and enema study with anastomotic dehiscence and stricture, conservative management POD152: ongoing stricture POD194: anastomotic revision with coloanal pull-through POD242: resolved	POD271
8	POD14: readmission with pelvic abscess, IV antibiotics POD17: Persistent pain, CT enema with AL, reop with pelvic washout and drainage POD19: no improvement, reop with anastomotic takedown, end colostomy, and mucous fistula	POD64: ongoing pelvic abscess, IR drainage	POD242: Hartman’s reversal	POD304
9	POD4: fecal drainage from drain with sepsis, reop with transanal repair of anastomotic dehiscence, pelvic washout of abscess	Ongoing dehiscence, presacral cavity	POD259, POD513: reop with EUA/debridement for ongoing dehiscence and large presacral cavity POD322: development of PC POD538: conversion of ileostomy to end colostomy at time of HIPEC and tumor debulking	Never reversed
10	POD13: Outpatient EUA concerning for ischemic conduit, readmission, reop with pelvic washout of abscess, anastomotic takedown, resection of ischemic conduit, end colostomy, and mucous fistula			Never reversed
Delayed anastomotic complications (> 30 days)				
11		POD32: asymptomatic stricture and dehiscence on endoscopy, required 6 sequential endoscopic dilations	POD151: endoscopy, resolved POD241: enema study, resolved	POD249
12		POD56: small asymptomatic anastomotic defect on endoscopy, no intervention	POD117: ongoing POD177: resolved	POD177
13		POD37: pelvic abscess, treated with oral antibiotics POD51: CT scan, resolved POD85: endoscopy, resolved		POD189
14		POD49: asymptomatic anastomotic dehiscence, endoscopy with conduit retraction, no intervention	POD173: ongoing anastomotic stricture POD209: endoscopy dilation POD265: anastomotic revision by transanal circumferential stricture resection and sleeve mobilization POD338: endoscopy, resolved	POD427
15		POD58: small asymptomatic anastomotic defect on endoscopy, no intervention POD85: resolved		POD103
16			POD172: asymptomatic anastomotic stricture, operative dilation, resolved	POD226
17			POD95: asymptomatic anastomotic sinus on endoscopy, no intervention POD172: resolved	POD250
18			POD349: asymptomatic stricture and dehiscence POD539: ongoing POD553: revision of anastomosis with coloanal pull-through	POD695

^a^POD refers to time (in days) from index surgery (taTME)

^b^Resolution of anastomotic complication as determined by gastrograffin enema, sigmoidoscopy, anoscopy, or CT with rectal contrast

*POD* postoperative day; *AL* anastomotic leak; *CT* computed tomography; *IV* intravenous; *IR* interventional radiology; *EUA* exam under anesthesia; *PC* peritoneal carcinomatosis; *HIPEC* heated intraperitoneal chemotherapy

Among late anastomotic complications diagnosed between POD30 and POD349, 3 were described as small defects or sinuses on endoscopy and resolved without any intervention, one was a pelvic abscess identified on POD37 and resolved with oral antibiotics, while one was an anastomotic stricture that required a single operative dilatation. In all 5 cases, stoma reversal was achieved between POD103 and POD250. In 3 patients with asymptomatic late anastomotic dehiscence and stricture identified on radiology and/or endoscopy, one resolved with serial dilatations with stoma closure on POD249, and 2 eventually required anastomotic revision with transanal sleeve advancement or coloanal pull-through, with delayed ileostomy closure between POD427 and POD695 (Tables 4 and 5, Fig. 4b).

Overall, 72.2% (13/18) of all anastomotic complications identified over the course of this trial required ≥ 1 surgical re-intervention including 90% of early complications, diagnosed within 30 days, and 50% of late complications, diagnosed after 30 days. Stoma closure was achieved in 80% of patients with early complications vs 100% of patients with late complications. Among interventions performed for anastomotic failure, anastomotic takedown was performed in 6 patients followed by immediate (4) or delayed reconstruction (1). Anastomotic failure resulted in permanent colostomy in 2 patients.

Anastomotic complications were reported among 21.8% (12/55) of patients who underwent intraoperative FA perfusion assessment vs 13.3% (6/45) among those who did not, and among 17.4% (8/46) vs 44% (4/9) of patients with adequate vs inadequate perfusion (Table 6). By univariable cox proportional hazards regression analysis, male sex (HR 7.52 [0.99, 56.75]; *p* = 0.0502) and inadequate bowel perfusion by FA (HR 3.3 [0.96, 11.32]; *p* = 0.0572) were associated with higher risk of anastomotic complications, but neither variable reached statistical significance (Table 6).

**Table 6:** Univariate cox regression analysis of risk factors for anastomotic leak

	No anastomotic complication *n* = 82	Anastomotic complication *n* = 18	Hazard ratio [95% CI]	*p* value
Age				
< 65	63 (76.8%)	17 (94.4%)	Reference	0.1217
≥ 65	19 (23.2%)	1 (5.6%)	0.20 [0.02, 1.52]	
Sex				
Female	29 (35.4%)	1 (5.6%)	Reference	0.0502
Male	53 (64.6%)	17 (94.4%)	7.52 [0.99, 56.75]	
BMI				
< 30	56 (68.3%)	11 (61.1%)	Reference	0.7926
≥ 30	26 (31.7%)	7 (38.9%)	1.13 [0.43, 2.96]	
ASA				
< 3	50 (61.0%)	10 (55.6%)	Reference	0.5137
≥ 3	32 (39.0%)	8 (44.4%)	1.37 [0.53, 3.56]	
Diabetes				
No	73 (89.0%)	16 (88.9%)	Reference	0.8870
Yes	9 (11.0%)	2 (11.1%)	0.89 [0.20, 3.98]	
Smoking				
No	75 (91.5%)	18 (100.0%)	Reference	Not-Est
Yes	7 (8.5%)	0 (0.0%)	Not-Est	
Clinical AJCC stage				
I	30 (36.6%)	5 (27.8%)	Reference	0.4438
II	16 (19.5%)	6 (33.3%)	1.62 [0.47, 5.61]	
III	36 (43.9%)	7 (38.9%)	1.13 [0.35, 3.57]	0.8335
Distance from anal verge (cm)				
≤ 6 cm	56 (68.3%)	11 (61.1%)	Reference	0.3828
> 6 cm	26 (31.7%)	7 (38.9%)	1.54 [0.58, 4.07]	
Neoadjuvant treatment				
No neoadjuvant treatment	24 (29.3%)	5 (27.8%)	Reference	Non-Est
Chemotherapy alone	2 (2.4%)	0 (0.0%)	Non-Est	0.7145
Long course CRT	42 (51.2%)	9 (50.0%)	0.81 [0.26, 2.50]	Non-Est
Short course RT	1 (1.2%)	0 (0.0%)	Non-Est	0.4715
Total neoadjuvant therapy	13 (15.9%)	4 (22.2%)	1.62 [0.43, 6.05]	
Days from end of neoadjuvant RT to surgery, per week increase	75.5 [68, 82]	70 [61, 79]	1.00 [0.90, 1.11]	0.9850
EBL, per 100 mL increase	100.0 [0.0, 660.0]	105.0 [50.0,700.0]	1.45 [0.81, 2.61]	0.2063
Partial ISR or mucosectomy				
No	50 (61.0%)	12 (66.7%)	Reference	0.4548
Yes	32 (39.0%)	6 (33.3%)	0.69 [0.25, 1.85]	
FA perfusion assessment				
No	39 (47.6%)	6 (33.3%)	Reference	0.2126
Yes	43 (52.4%)	12 (66.7%)	1.87 [0.70, 5.01]	
FA perfusion assessment outcome				
Adequate	38 (46.3%)	8 (44.4%)	Reference	0.0572
Inadequate	5 (11.6%)	4 (33.3%)	3.30 [0.96, 11.32]	
Type of anastomosis				0.4928
Stapled	37 (45.1%)	9 (50.0%)	Reference	
Handsewn	45 (54.9%)	9 (50.0%)	0.71 [0.27,1.85]	
Size of stapler (among stapled anastomosis)				
28	13 (35.1%)	2 (22.2%)	Reference	
29	9 (24.3%)	5 (55.6%)	3.04 [0.59, 15.72]	0.1834
31	9 (24.3%)	2 (22.2%)	1.20 [0.16, 8.63]	0.8563
33	6 (16.2%)	0 (0.0%)	Non-Est	Non-Est
Splenic flexure takedown				
No	5 (6.1%)	1 (5.6%)	Reference	0.9406
Yes	77 (93.9%)	17 (94.4%)	1.08 [0.14, 8.14]	
Final TME grade				
Complete	58 (70.7%)	11 (61.1%)	Reference	
Incomplete	8 (9.8%)	2 (11.1%)	1.40 [0.31, 6.38]	0.6565
Near complete	16 (19.5%)	5 (27.8%)	1.26 [0.42, 3.74]	0.6696
CRP > = 145 on POD3				
No	45 (80.4%)	7 (63.6%)	Reference	0.3022
Yes	11 (19.6%)	4 (36.4%)	1.91 [0.56, 6.57]	

*Non-Est* not estimable due to 0 patients with incomplete TME with level of risk factor; *ISR* intersphincteric resection; *FA* fluorescence angiography

**Fig. 5:**
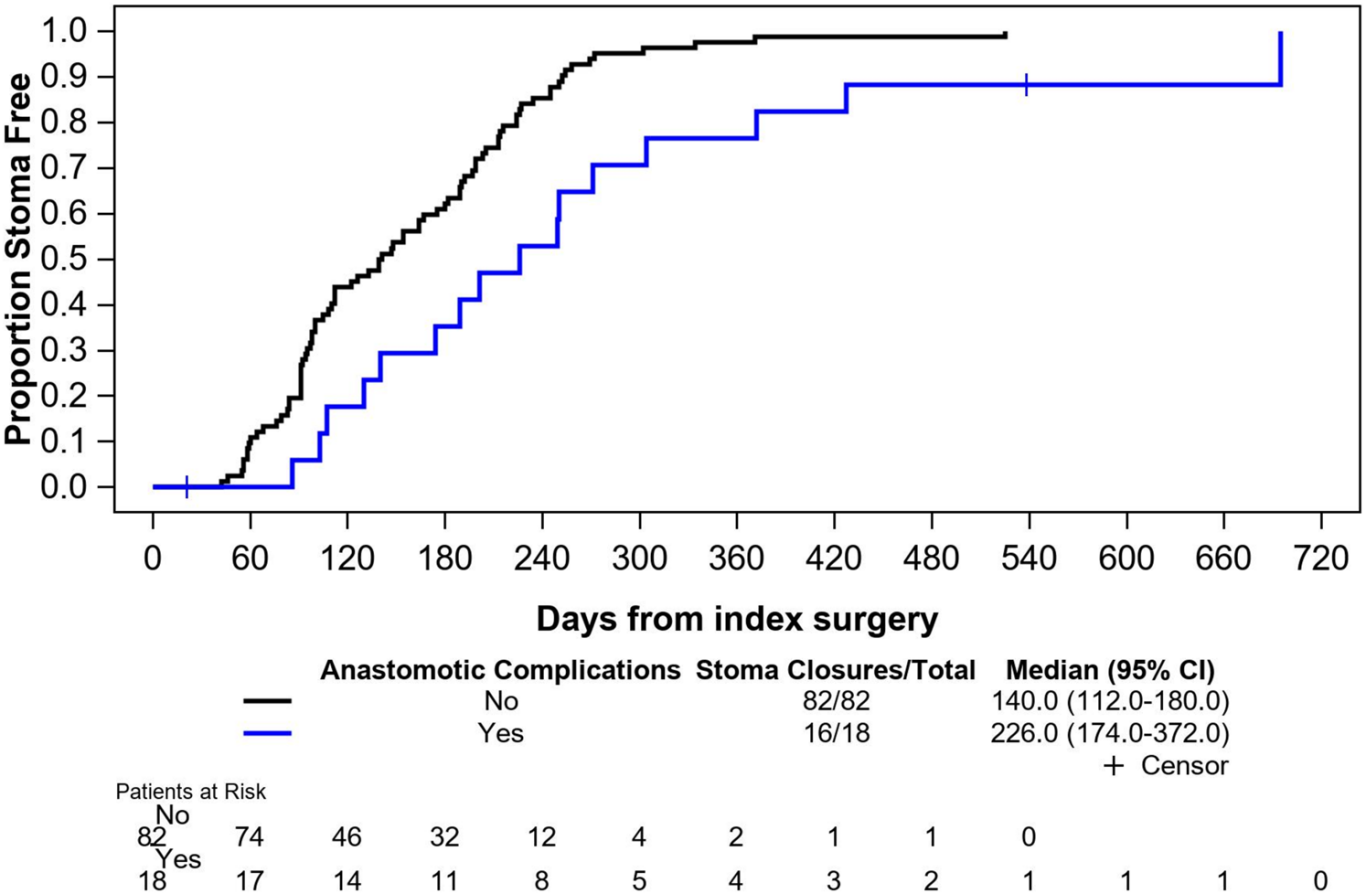
Kaplan–Meier curve for time from taTME to stoma closure in patients with and without anastomotic complications (days)

The overall stoma closure rate was 93% at 12 months and 98% at 23 months follow-up and median time to reversal was 154 (IQR 94–224) days (Table 4, Fig. 5). Stoma reversal rate was 89 vs 100% in patients with and without anastomotic complications, and median time to closure of 226 (IQR 140–304) days vs 140 (IQR 91–213) days, respectively (*p* = 0.0015). Factors contributing to delays in stoma reversal beyond 90 days were related to adjuvant treatment, anastomotic complications, COVID-related delays in elective surgery, patient preference, or other unclear reasons (Table 4).

### Oncologic outcomes

Final pathologic staging demonstrated no residual disease (pT0N0) in 8 of 10 cases where completion taTME had been performed after polypectomy or local excision of a cT2 or cT1 tumor with high-risk histopathologic features. Complete pathologic response (pCR) was demonstrated in 25/71 (35.2%) of patients who received neoadjuvant treatment. The pCR rate was 41% (7/17) post TNT, 33% (17/51) post CRT, and 50% (1/2) post chemotherapy only (Table 7). The median number of harvested lymph nodes was 19 (IQR 14.5–26). Following reconciliation between site pathologists and central blinded reviewers, final TME grading was complete in 69%, near complete in 21%, and incomplete in 10% of specimens. There was no specimen perforation, but 1 case had positive CRM and 1 case had positive distal resection margin (DRM). After adjusting for the number of TME specimens with residual tumor, the positive margin rate was 3% (2/67). Adjuvant chemotherapy was completed in 46 patients, as recommended by MDT based on preoperative clinical stage or final pathology stage.

**Table 7:** Pathology outcomes

	*n* = 100 patients
TME grade	
Complete	69 (69.0%)
Near complete	21 (21.0%)
Incomplete	10 (10.0%)
Pathology tumor stage	
pT0^a^/ypT0^b^	8 (8.0%)/25 (25.0%)
pTis/ypTis	0 (0.0%)/1 (1.0%)
pT1/ypT1	10 (10.0%)/11 (11.0%)
pT2/ypT2	8 (8.0%)/21 (21.0%)
pT3/ypT3	3 (3.0%)/13 (13.0%)
Pathology node stage	
pN0/ypN0	26 (26.0%)/56 (56.0%)
pN1/ypN1	2 (2.0%)/14 (14.0%)
pN2/ypN2	1 (1.0%)/1.0 (1.0%)
Tumor regression grade, among 71 treated tumors	
Grade 0	26 (36.6%)
Grade 1	22 (31.0%)
Grade 2	20 (28.2%)
Grade 3	3 (4.2%)
Lymphovascular invasion, among 67 residual tumors	
Absent	51 (76.1%)
Present	14 (20.9%)
Suspicious	2 (3.0%)
Perineural invasion, among 67 residual tumors	
Absent	53 (79.1%)
Present	14 (20.9%)
Positive margin, among 67 residual tumors	
Any	2 (3.0%)
DRM	1 (1.5%)
CRM	1 (1.5%)
Total number of nodes examined, median [IQR]	19 [14.5, 26]
Adjuvant treatment	46 (46.0%)

^a^No residual disease in patients who underwent previous local excision via TAMIS (*n* = 5) or polypectomy (*n* = 3)

^b^Complete pathological response to neoadjuvant therapy

*TME* total mesorectal excision; y, staging determined after neoadjuvant therapy; *DRM* distal resection margin; *CRM* circumferential resection margin

By univariable log-binomial regression analysis, ASA ≥ 3 (RR 13.50 [1.77, 102.47]; *p* = 0.0118), blood loss per 100 ml increase (RR 1.46 [1.14–1.87]; *p* = 0.0027), and longer time interval between end of neoadjuvant RT (NRT) to surgery (RR 1.07 [1.01, 1.14]; *p* = 0.0297, Table 8) were associated with a significant increase in the risk of incomplete TME (Table 8).

**Table 8:** Univariate regression analysis of risk factors for incomplete TME

	Incomplete TME *n* = 10	Complete/near-complete TME *n* = 90	Relative risk [95% CI]	*p* value
Age				
< 65	7 (70.0%)	73 (81.1%)	Reference	0.4020
≥ 65	3 (30.0%)	17 (18.9%)	1.71 [0.48, 6.04]	
Sex				
Female	3 (30.0%)	27 (30.0%)	Reference	1.0000
Male	7 (70.0%)	63 (70.0%)	1.00 [0.27, 3.60]	
BMI				
< 30	5 (50.0%)	62 (68.9%)	Reference	0.2344
≥ 30	5 (50.0%)	28 (31.1%)	2.03 [0.63, 6.52]	
ASA				
< 3	1 (10.0%)	59 (65.6%)	Reference	0.0118*
≥ 3	9 (90.0%)	31 (34.4%)	13.50 [1.77, 102.47]	
Diabetic				
No	9 (90.0%)	80 (88.9%)	Reference	
Yes	1 (10.0%)	10 (11.1%)	0.89 [0.12, 6.43]	0.9156
Smoking				
No	10 (100.0%)	83 (92.2%)	Reference	
Yes	0 (0.0%)	7 (7.8%)	0.53 [0.02, 12.10]	0.6908
Clinical AJCC stage				
I	2 (20.0%)	33 (36.7%)	Reference	
II	2 (20.0%)	20 (22.2%)	1.59 [0.24,10.48]	0.6294
III	6 (60.0%)	37 (41.1%)	2.44 [0.52, 11.35]	0.2549
Distance from anal verge (cm)				
≤ 6 cm	6 (60%)	61 (67.8%)	Reference	
> 6 cm	4 (40%)	29 (32.2%)	1.35 [0.41, 4.47]	0.6194
Neoadjuvant treatment				
No neoadjuvant treatment	2 (20.0%)	27 (30.0%)	Reference	
Neoadjuvant treatment	8 (80.0%)	63 (70.0%)	1.63 [0.37, 7.24]	0.5179
Neoadjuvant treatment				
No neoadjuvant treatment	2 (20.0%)	27 (30.0%)	Reference	
Long course CRT	3 (30%)	48 (53.3%)	0.85 [0.15, 4.81]	0.8570
Chemotherapy alone	0 (0%)	2 (2.2%)	Non-Est	Non-Est
Short course RT	0 (0%)	1 (1.1%)	Non-Est	Non-Est
Total neoadjuvant therapy	5 (50%)	12 (13.3%)	4.26 [0.93, 19.63]	0.0626
Days from end of neoadjuvant RT to surgery, per week increase	85.5 [77, 147]	74 [66, 80]	1.07 [1.01, 1.14]	0.0297*
EBL, per 100 mL increase	200.0 [40.0, 700.0]	100 [0.0, 660.0]	1.46 [1.14, 1.87]	0.0027*
Type of anastomosis				
Stapled	5 (50.0%)	41 (45.6%)	Reference	
Handsewn	5 (50.0%)	49 (54.4%)	0.85 [0.26, 2.76]	0.7892
Pathology T stage				
(y)pT0	4 (40.0%)	29 (32.2%)	Reference	
(y)pT1/Tis	4 (40.0%)	18 (20.0%)	1.50 [0.42, 5.38]	0.5336
(y)pT2	1 (10.0%)	28 (31.1%)	0.28 [0.03, 2.40]	0.2482
(y)pT3	1 (10.0%)	15 (16.7%)	0.52 [0.06, 4.25]	0.5381
Pathology N stage				
(y)pN0	9 (90.0%)	73 (81.1%)	Reference	
(y)pN1	1 (10.0%)	15 (16.7%)	0.57 [0.08, 4.19]	0.5802
(y)pN2	0 (0%)	2 (2.2%)	Non-Est	
Positive margins in patients with residual tumor				
No	6 (60.0%)	59 (65.6%)	Reference	
Yes	0 (0.0%)	2 (2.2%)	1.83 [0.04, 82.42]	0.7554
Partial ISR or mucosectomy				
No	6 (60.0%)	56 (62.2%)	Reference	
Yes	4 (40.0%)	34 (37.8%)	1.08 [0.32, 3.60]	0.8907

*CI* confidence interval; *Non-Est* not estimable due to 0 patients with incomplete TME with level of risk factor; *ISR* intersphincteric resection

*p < 0.05

## Discussion

This phase II multicenter North American trial is the most recent large prospective trial to confirm the procedural safety, favorable pathologic outcomes, and low rates of conversion achieved with taTME when performed by experienced surgeons using a 2-team hybrid approach. The 2% abdominal open conversion rate is consistent with the 0–5.1% conversion rates reported in large taTME series of patients, and was achieved despite a predominantly male cohort with the highest proportion of patients with BMI ≥ 30 reported to date (33%) and in whom 67% of tumors were located ≤ 6 cm from the AV [14, 19, 22, 45–48]. The current trial results validate prior findings that combining taTME with a laparoscopic or robotic abdominal approach enables completion of minimally invasive sphincter-preserving TME, particularly in obese patients with low rectal tumors. Intraoperative complications related to transanal dissection are rare and included 2 vaginal injuries but no rectal perforation or urethral injury, which underscores the experience of participating surgeons [15].

The primary endpoint of the trial was mesorectal TME grade, an important pathologic endpoint and strong predictor of local recurrence and disease-free survival that also serves as an accurate marker of surgical quality [34, 49–51]. Complete and near-complete TME grade was achieved in 90% with a 10% rate of incomplete TME and 3% rate of positive margins. These results are in line with the 1.5–11% rates of incomplete TME reported in large retrospective taTME series [22, 46, 47, 52], and the 8% and 9.3% rates reported in the laparoscopic arm of the ACOSOG RCT and the robotic arm of the ROLAAR RCT, respectively [53, 54]. But this rate is significantly higher than the recently reported 0% rate of incomplete TME in the taTME arms of the 2 laparoscopic vs taTME RCT in China and Spain [55, 56]. Relative to the RCT by Liu et al. in which tumors were similarly located (5.0 vs 5.8 cm from the AV) in patients with higher BMI (22.9 vs 27.8), our higher rate of incomplete TME may reflect a more challenging dissection in a previously radiated field, given that 69% of our taTME cohort was treated with NRT vs only 14% of the Chinese cohort. Among 10 incomplete TME specimens in our trial, 8 occurred in patients treated with neoadjuvant long-course CRT or TNT, and for every 1 week increase in time from end of NRT to surgery, the risk of incomplete TME increased by 7%.

There is a paucity of data regarding the impact of delayed time to TME following completion of NRT on TME grade, and this has not been described in prior taTME series or trials. The 2015 TIMING phase II trial assigned patients with locally advanced tumors into 4 groups treated with CRT: CRT followed by 2, 4, or 6 cycles of mFOLFOX6, effectively increasing the interval to TME by 6 up to 20 weeks [57]. While surgeons reported an increase in pelvic fibrosis dissection with longer delays to surgery, they reported no differences in technical difficulty, surgical complications, R1 resection rate, or sphincter preservation. However, the authors did not analyze the impact of longer delays on TME grade. The UNICANCER-PRODIGE23 RCT comparing standard CRT to FOLFIRINOX followed by CRT for patients with locally advanced cancer, reported no difference in mesorectal grade between cohorts (5 vs 8% rate of incomplete TME). However, given that TNT was administered as induction treatment, there was no difference in the time interval between the end of NRT and surgery, which was relatively short in both groups (54.5 and 55 days, respectively) [58]. Given the increasing adoption of TNT worldwide in an effort to increase the rate of non-operative management, the impact of this delay on perioperative morbidity as well as TME quality will need to be further examined.

In the Spanish laparoscopic vs taTME RCT where a 0% rate of incomplete TME was reported in the taTME arm, a similar proportion of patients were treated with NRT (69.1% vs 69%) but the NRT-to-TME interval was not specified. The difference in rate of incomplete TME may reflect a higher level of technical difficulty during transanal dissection based on differences in median tumor height (8.0 cm vs 5.8 cm), and in the proportion of cases where ISR was performed (0% vs 36%) in the Spanish vs North American taTME cohorts, respectively [56]. In this phase II trial, ASA ≥ 3 and increased blood loss were identified as significant risk factors for incomplete TME, with increased blood loss generally expected during more difficult pelvic dissections. Lastly, higher rates of incomplete TME grading in this trial likely reflect more stringent quality control measures used for pathological specimen assessment. Concordance in TME grading between site pathologists and blinded central reviewers was evaluated, and major discrepancies in TME grading were resolved with final grade assigned based on consensus agreement. Neither of the recent taTME RCTs specifically incorporated quality control measures to validate TME grading. Importantly, Liu et al. in reporting a 0% rate of incomplete TME acknowledged that “an overestimation bias for successful resection still remains a certain possibility” [55].

Regarding other pathologic endpoints, our 1.5% positive CRM and 1.5% positive DRM rates compare favorably with the 1.4–12.7% CRM and 0–2.5% DRM-positive rates published in large taTME series [19, 22, 23, 59] and with the 0.9%/0%, and 0.4%/12.7% DRM-positive rates in the Liu et al. and Serra et al. taTME vs lap TME RCTs, respectively [55, 56]. TaTME was combined with ISR in 36% of patients whose tumors were located at a median of 4 cm from the AV in our North American trial, compared with 14.9% and 0% of the Chinese and Spanish taTME cohorts where median tumor height was 5 and 8 cm, from the AV, respectively [55, 56]. These results highlight the role that ISR will continue to play in facilitating sphincter preservation as well as R0 resection for low rectal tumors, without adverse impact on oncologic outcomes [59–64].

This prospective trial is unique in that it provided extended capture of early and late postoperative complications following taTME, including outcomes of all anastomoses until primary stoma closure or permanent stoma creation. Overall, 71 complications among 49 patients and 82 complications among 56 patients were reported at 30 and 90 days, respectively, which is relatively high relative to other taTME publications; but the majority were minor, with severe (CD ≥ 3) complications only reported in 14 and 17 patients at 30 and 90 days, respectively. Ileus and stoma-related dehydration accounted for 35% of 30-day complications and 47% of readmissions, and were a direct consequence of loop ileostomy creation. Although published rates of diversion following sphincter-preserving taTME vary from 55.7 to 100% [14, 21, 22, 55, 65], our trial reflects standard practice in North American centers where high-risk colorectal and all coloanal anastomoses are routinely diverted [22, 53]. Fecal diversion is particularly prevalent when ISR and handsewn anastomoses are performed, and in patients receiving extended courses of radiotherapy [13, 14, 66]. Urinary retention contributed to 27% of 30-day complications and occurred at a higher rate than the reported 2–13% incidence in other taTME series [9, 21, 67], which may be related to the timing of catheter removal. The impact of taTME procedures on long-term urinary and sexual functional will be reported in a later publication.

Anastomotic complications accounted for 10% of the trial 30-day morbidity. Given the lack of agreement with standardized definitions and diagnostic criteria for anastomotic leaks, we opted to report all anastomotic complications identified and treated throughout the trial clinical follow-up period. This broader framework for reporting early and late anastomotic complications, which also incorporates stoma closure and non-closure rates, has been used by other groups when reporting outcomes of restorative proctectomy from large colorectal cancer registries, clinical trials, institutional series, and the international taTME registry [13, 14, 68–72]. While the reported 30-day anastomotic leak rates range between 2 and 10% in most large TME and taTME series [9, 21, 53, 55, 56], these rates are not inclusive of pelvic abscess or phlegmon adjacent to anastomoses, nor of leaks or sequelae of leaks (sinus, fistula, stricture) identified beyond 30 days of surgery. In contrast, the TME registries and trials that intentionally report early and late-occurring anastomotic complications up to 1 year post TME, report rates as high as 20% [13, 68–71]. Using this anastomotic complication reporting framework, at a median follow-up of 5 (IQR 3–7) months, anastomotic complications were identified in 10% by 30 days, 15% by 90 days, and 18% by 349 days in our cohort. The 10% early leak rate is consistent with the 6.3–11% 30-day leak rates reported in large taTME series [20, 22, 24, 28, 38, 55, 59] and the 18% overall anastomotic complication rate is similar to the 15.7%-17.7% rates reported at 5–12 months post-taTME in the international registry and other retrospective series [13, 14]. Although the incidence of anastomotic complications was higher in male patients (24.3 vs 3.3%, *p* = 0.0502), this did not reach statistical significance in this study, but it has been identified as a risk factor in other taTME studies [21–23, 26, 38, 52, 59]. The role of intraoperative perfusion assessment on anastomotic failure had not been previously reported in the setting of a prospective phase II TME trial. In the current study, intraoperative fluorescence angiography (FA) was used at 4 of the 11 study sites, with the decision to use FA made on a case-by-case basis. Perfusion assessment was reported to have altered the operative plan in 14.5% of cases, which is consistent with 2 prior retrospective taTME series demonstrating a change in planned proximal resection margin in 18 and 28.7% of patients when FA was used [45, 73]. Despite active intervention to correct inadequate perfusion identified on FA, the incidence of anastomotic complications was higher in the FA group, which likely reflects selection bias with preferential use of FA in cases where ischemia was suspected. Although documentation of adequate bowel perfusion by FA was not entirely protective against anastomotic complications, it was associated with a lower risk of anastomotic complication, although this did not reach statistical significance.

This trial highlighted emerging trends in the management of anastomotic complications among North American rectal cancer practices, including the use of transanal rescue approaches to manage early symptomatic and asymptomatic leaks, and anastomotic revision with coloanal anastomosis for refractory anastomotic complications that failed initial interventions. Among early anastomotic complications, given that all anastomoses had been diverted, some leaks were diagnosed in patients who were otherwise asymptomatic. One leak was suspected based on CRP elevation and another diagnosed on CT scan with rectal contrast performed to evaluate for possible early ileostomy closure. Whether symptomatic or not, early leaks were managed within 30 days with transanal repair attempted in 75% of reoperations. Early re-intervention led to resolution and ileostomy reversal in 80% patients with ≤ 1 additional re-intervention. All anastomotic complications diagnosed > 30 days postoperatively were managed with 0–1 major surgical intervention, and stoma reversal was achieved in all cases. Overall, 2 patients with anastomotic complications were converted to permanent colostomy due to colonic conduit ischemia and refractory anastomotic dehiscence in the setting of disease progression, respectively. There was no mortality related to anastomotic complications and 89% stoma-free rate was achieved at 2 years in patients with anastomotic complications. The trial’s overall 93% 1-year and 98% 2-year stoma reversal rates are higher than those in other trials that report long-term outcomes of sphincter-preserving TME with or without a primary fecal diversion [74]. Among 595 patients in the 2015–2017 Dutch ColoRectal Audit (DCRA) that underwent sphincter-preserving TME, the 1-year stoma closure was 81.3 with 19.7% of patients requiring a permanent stoma [72]. In a meta-analysis that included 10 studies, the pooled rate of non-closure of diverting stomas following LAR for rectal cancer was 19% (95% CI 12–24%; *p* < 0.001) at follow-up ranging from 13 months to 7.1 years. Risks factors for non-closure included surgical complications, anastomotic leakage, stage IV tumors, and local recurrence [60]. Long-term stoma closure rates have been reported in 7 retrospective taTME series and large registries, and range from 56.5% at 4.73 months to 89.5% at 27 months [9, 21, 22, 28, 59, 61, 62, 65]. However, long-term stoma closure rates have not been previously reported in the context of a prospective rectal cancer trial. The high stoma closure rate in the current trial is most likely attributed to early management of symptomatic leaks, which helped minimize leak-related morbidity and mortality, as well as the aggressive management for refractory anastomotic defects and sequelae. Redo-coloanal anastomosis was performed in 3 cases with refractory anastomotic dehiscence or stricture 7–18 months post-taTME with successful ileostomy reversal in all 3 cases. Delayed anastomotic reconstruction for failed anastomoses has been reported in very few small TME series [63, 64, 75]. In a recent systematic review of 9 studies and 291 patients, redo-anastomosis was performed at a median 14–41 months post the index procedure, with a 79% rate of stoma closure and 16% morbidity rate [64, 75].

A limitation of the trial concerns strict inclusion and exclusion criteria for enrollment. Similar to the two recent RCT’s of lap vs taTME, cT3b tumors with predicted positive CRM, sphincter invasion or cT4 tumors on preoperative staging were excluded, even when downstaging was achieved with neoadjuvant treatment and restaging. While this was necessary in order to establish the feasibility and safety of taTME on a homogenous cohort of resectable cancer cases, our cohort does not entirely reflect current practice where taTME has become the preferred approach for tumors with incomplete response or tumor regrowth following neoadjuvant treatment. While ongoing RCTs will validate the long-term oncologic safety of taTME and evaluate functional results relative to laparoscopic TME, it will become important to understand the role that taTME plays in contemporary rectal cancer practices in the era of robotic surgery, TNT and an increased demand for sphincter preservation among young patients.

## Conclusions

In this prospective phase II multicenter North American trial, the procedural and preliminary oncologic safety of taTME was demonstrated. In a predominantly male cohort with low rectal tumors and median BMI 27.8, a 2-team taTME approach achieved acceptable rates of complete and near-complete mesorectal grade with a low rate of open conversion. Continued monitoring and management of anastomotic complications resulted in high stoma closure rates.

### Supplementary Information

Below is the link to the electronic supplementary material.

Supplementary file1 (PDF 1292 KB)
