# Supplementary material for: Multicenter phase II trial of transanal total mesorectal excision for rectal cancer: preliminary results
Source: Surg Endosc. 2023 Sep 12;37(12):9483–508. doi: 10.1007/s00464-023-10266-9 (PMC10709232; doi:10.1007/s00464-023-10266-9)
Supplement: Supplementary file 1 — Supplementary file1 (PDF 1292 KB) [file 464_2023_10266_MOESM1_ESM.pdf]

# Administrative Cover Sheet

# Multicenter Phase II Study of Transanal Total Mesorectal Excision (taTME) with Laparoscopic Assistance for Rectal Cancer

**Study Type**

Investigator Initiated

Amendment 5

**Protocol Version 6**

**Coordinating Center**

Mount Sinai Hospital

1 Gustave L. Levy Place Box 1259

New York, NY 10029

**DSMC**

Tisch Cancer Center DSMC

**External Monitor**

Biomedical Research Alliance of New York

**SPONSORS**

Society of the American Gastrointestinal and Endoscopic Surgeons (Administrative Sponsor)
American Society of Colon and Rectal Surgeons Research Foundation Grant

**Overall PI**
Patricia Sylla, MD

# Clinical Study Protocol

# Version 6

## Multicenter Phase II Study of Transanal Total Mesorectal Excision (taTME) with Laparoscopic Assistance for Rectal Cancer

**Short Title**

Multicenter Phase II Study of taTME

Amendment 5

**Protocol Version 6**

**Coordinating Center**

Mount Sinai Hospital

1 Gustave L. Levy Place Box 1259

New York, NY 10029

**Overall PI**

Patricia Sylla, MD

**STUDY SYNOPSIS**

| |
|---|
| **STUDY TITLE**<br><br>Multicenter Phase II Study of Transanal Total Mesorectal Excision (taTME) With Laparoscopic Assistance for Rectal Cancer |
| **SPONSORS**<br><br>SAGES (Society of American Gastrointestinal and Endoscopic Surgeons)<br>ASCRS (The American Society of Colon and Rectal Surgeons Research Foundation) |
| **COORDINATING CENTER**<br><br>Mount Sinai Hospital, New York, NY<br>Biomedical Research Alliance of New York (BRANY), New York, NY (Study Monitor) |
| **OVERALL PI**<br><br>Patricia Sylla, MD |
| **NUMBER OF STUDY SITES**<br><br>11 |
| **STUDY SITE ELIGIBILITY**<br><br>• Performance of ≥ 20 rectal cancer resections with TME per year, with ≥ 20 performed laparoscopically (+/- hand-assistance) and/or robotically-assisted with sphincter preservation (LAR) within the preceding 12 months, and submission of operative and pathology reports for 10 of those cases for review. Proof of participation in the ACOSOG Z6051 trial will substitute for this requirement.<br>• Institutions anticipated to meet the minimum accrual target of 10 subjects enrolled over 2 years<br>• Institutions with multidisciplinary tumor board review of all rectal cancer cases with treatment plan generated by consensus<br>• Performance of ≥ 20 TEM, TEO or TAMIS procedures within the preceding 24 months by participating transanal surgeons<br>• Performance of ≥ 5 taTME cases for cancer in the preceding 12 months (LAR or APR) by the participating institution, with review of operative and pathology reports, photographs of gross TME specimens and unedited transanal video recordings of 2 taTME cases including at least one male patient |
| **STUDY OBJECTIVES**<br><br>**Primary:**<br>• Determine whether taTME is non-inferior to standard LAR by evaluation of the proportion of subjects who reach efficacy of the TME i.e. in whom good quality mesorectal excision is achieved (complete and near-complete mesorectum)<br>**Secondary:**<br>• Pathology assessment of specimen (synoptic report, Appendix B)<br>• Incidence of 30-day perioperative complications including intraoperative and postoperative complications<br>• Incidence of long-term (> 30 days) complications<br>• Assessment of changes in functional results (bowel function, urinary function, and sexual function)<br>• Assessment of 3-year oncologic outcomes (recurrence and survival) |
| **STUDY DESIGN**<br><br>• This is a 5-year phase II multicenter study to evaluate the non-inferiority of transanal total mesorectal excision (taTME) with laparoscopic or robotic assistance relative to standard LAR. A total of 100 participants with resectable rectal cancer located within 10 cm from the anal verge will |

be enrolled across 11 US study sites. All eligible participants will consist of patients who are candidates for laparoscopic or robotic LAR with TME with or without a temporary diverting ileostomy for preoperatively staged T1, T2 and T3 node positive and node negative rectal cancer. The primary endpoint is rate of complete and near-complete mesorectal excision achieved with taTME as defined by standard pathology assessment. Secondary endpoints include pathology assessment of the specimen, 30-day perioperative complications, long-term complications, changes in functional outcomes, and 3-year oncologic outcomes. Functional outcomes including bowel function, fecal continence, sexual and urinary function, will be assessed preoperatively and at 2 postoperative time points.

**Accrual and Study Duration**

• Pre-screening of more than 20 rectal cancer patients per year per study site is anticipated. Enrollment of 100 subjects will occur over a 2-year accrual period across 11 study sites in the US. At each site, accrual of at least 5 patients per year is anticipated. The study duration will be up to 5 years including 3 years of accrual and 2 to 5 years of long-term oncology follow-up.

• The study is expected to begin in the first half of 2017 and complete enrollment within 2 years. The study is expected to complete in 2022.

**Screening:**

• Upon referral for management of newly diagnosed resectable rectal cancer, potential study participants will be screened for eligibility. Subjects' medical records, imaging studies, endoscopy and pathology reports will be reviewed at the institution's multidisciplinary tumor board conference. Subjects with preoperatively staged T1, T2 and T3, node positive or negative rectal adenocarcinoma located within 10 cm from the anal verge, with no evidence of metastasis, and who are eligible to undergo elective sphincter-preserving laparoscopic or robotic LAR, will be identified as potential study participants.

**Enrollment:**

• Stage I (T1/T2N0) and subset of Stage II rectal cancer (T3N0) subjects who will proceed directly to TME without neoadjuvant treatment: Upon signing the study consent, eligible subjects are enrolled in the study, assigned a study enrollment number, and complete preoperative (baseline) functional questionnaires. Upon signing the surgical consent for the study procedure (taTME), participants are registered in the study with the coordinating center and assigned a study registration number.

• Stage II and III rectal cancer (T3, any N) subjects who will receive neoadjuvant treatment (short-course radiotherapy or full-course CRT with or without induction or consolidation chemotherapy): Eligible subjects are enrolled in the study, assigned a study enrollment number, and complete pre-treatment (baseline) and post-treatment functional questionnaires. Upon signing the surgical consent for the study procedure (taTME), subjects treated with short-course radiotherapy are registered to the study with the Coordinating Center and assigned a study registration number. Upon clinical evaluation of subjects treated with long-course CRT, study eligibility will be confirmed, the surgical consent for the study procedure obtained, registration completed and subjects will be assigned a study registration number.

• Stage II and III rectal cancer (T3, any N) subjects referred to the study site during or following neoadjuvant treatment at an outside institution will be screened for eligibility and enrolled in the study if they meet eligibility criteria.

**Study Procedure:**

• Study procedures will be scheduled to occur within 2 weeks of completion of short-course preoperative radiation therapy or 6-12 weeks following completion of neoadjuvant treatment. Preoperative preparation, mechanical bowel preparation and perioperative care will be performed based on standard practice.

• Study procedures will consist of transanal TME or TSME with laparoscopic or robotic assistance. Details of the operative steps, surgical technique and intraoperative complications will be

documented in CRFs. Briefly, laparoscopic or robotic abdominal access will be obtained followed by IMA and IMV pedicle transection, proximal colon mobilization and splenic flexure takedown if indicated. Transanal TME procedures may include intersphincteric resection (ISR) for very low tumors. Following pursestring closure of the rectum below the tumor, transanal endoscopic TME dissection will proceed circumferentially until the peritoneal cavity is entered anteriorly. Rectal mobilization and/or TME completion may be performed using sequential or combined transanal and abdominal assistance. Following specimen extraction (transanal or transabdominal), coloanal anastomosis will be completed with or without diverting loop ileostomy.

**TME Pathology Assessment and Quality Control**
Resected specimens will be processed and analyzed by the participating institution's pathology department according to standard TME protocol. De-identified photographs of all fresh TME specimens will be independently reviewed by the pathology review committee blinded to the source of the specimen.

**Postoperative Care and Follow-up:**
Participants will be managed according to standard postoperative protocols. Postoperative visits and oncology follow-up visits will occur as per standard practice and oncologic outcomes recorded on CRFs. All adverse events and postoperative complications occurring during the study period will be reported in CRFs and graded using the Clavien-Dindo system. Postoperative functional questionnaires will be obtained 6-8 months and 12-18 months postoperatively in non-diverted subjects. In diverted subjects, functional questionnaires will be collected 3-4 months and 9-10 months following ileostomy closure (or 12-18 months following the study procedure).

**SELECTION CRITERIA**

**Inclusion Criteria:**
- Males and females ≥ 18 years-old
- New diagnosis of histologically confirmed adenocarcinoma of the rectum
- Rectal cancer originally staged as T1, T2, or T3, node negative (N0) or node positive (N1, N2) by pelvic MRI
- ECOG performance status ≤ 2 (Karnofsky ≥ 60%)
- Circumferential radial margin uninvolved by tumor as assessed on pelvic MRI
- No evidence of metastasis on staging CT or PET CT scans of the chest, abdomen and pelvis
- Rectal cancer located within 10 cm from the anal verge based on proctoscopy and DRE
- Complete preoperative colonoscopy demonstrating no synchronous colon cancer
- Eligible to undergo laparoscopic or robotic low anterior resection with or without a temporary diverting stoma, based on multidisciplinary tumor board consensus
- Women of child-bearing potential and men must agree to use adequate contraception (hormonal or barrier method of birth control; abstinence) prior to study entry and for the duration of study participation
- Ability to understand and the willingness to sign a written informed consent document

**Exclusion Criteria**:
- Rectal cancer staged as T4 by pelvic MRI
- Participants with preoperatively staged T3, N1, or N2 tumors who return > 12 weeks following completion of neoadjuvant treatment
- Severely symptomatic rectal tumors (near-completely obstructing, symptomatic bleeding)
- Tumors invading into the internal sphincter muscle based on pelvic MRI
- Fecal incontinence at baseline
- Prior history of rectal resection
- Prior history of colorectal cancer
- History of inflammatory bowel disease
- Uncontrolled intercurrent illness including, but not limited to ongoing or active infection, symptomatic congestive heart failure, unstable angina pectoris, cardiac arrhythmia, or psychiatric illness/social situations that would limit compliance with study requirements

• Pregnant women
• Subjects who cannot read or understand English

**STUDY ENDPOINTS**

**Primary**
• Rate of complete and near-complete mesorectal excision achieved with taTME, based on standard guidelines on pathology assessment of TME specimens.

**Secondary**
• Pathology assessment of the specimen (synoptic report)
• 30-day perioperative and long-term complications
• Postoperative functional outcomes (defecatory, sexual and urinary dysfunction)
• Long-term oncologic outcomes following transanal TME

**Safety**
• Incidence of medical and surgical postoperative complications
• Clavien-Dindo grading of postoperative complications
• Incidence of adverse events

**DATA COLLECTION**

**Preoperative Assessment**
• Assessment of eligibility criteria
• Demographics, BMI, medical and surgical history
• Tumor location, rectal cancer staging, clinical evaluation
• Baseline colorectal functional outcome scores (COREFO), fecal incontinence scores (Wexner and FIQL), sexual function score (FSFI or IIEF) and urinary function score (IPSS) questionnaires prior to neoadjuvant treatment (if applicable), and preoperatively following completion of neoadjuvant treatment (if applicable)
• Neoadjuvant treatment received (agent, dose, and duration).
• Repeat clinical evaluation following completion of neoadjuvant treatment (if applicable) to confirm eligibility

**Intraoperative Assessment**
• Operating surgeons
• Procedures performed
• Transanal endoscopic platform and energy source used
• Operative time, blood loss
• Specimen extraction site
• Type of colorectal anastomosis
• Intraoperative complications
• Conversion, defined as completion of the proctectomy through an open abdominal incision (≥ 10 cm used for specimen extraction) due to difficulties or intraoperative complications

**Postoperative Assessment**
• Postoperative complications and adverse events
• TME synoptic pathology report and photographs of the gross TME specimen
• Duration of hospital stay(s)
• Immediate postoperative CRP value (POD 1-4 or less if shorter hospital stay)
• Follow-up clinical evaluation reports
• Adjuvant treatment received (if applicable)
• Follow-up surveillance imaging and blood test results
• Date of ileostomy closure (if applicable)
• Postoperative COREFO, Wexner, FIQL scores, FSFI or IIEF scores, IPSS scores obtained from questionnaires administered 6-8 months postoperatively if non-diverted subjects, 3-4 months post-ileostomy closure, and 12-18 months following the study procedure

**PLANNED INTERIM ANALYSIS**

**Interim Analysis and Study Stopping Rule**

• After 50% of the 100 study subjects have been enrolled and undergone the study procedure, interim analysis of futility of the mesorectal excision achieved by taTME will be performed by the coordinating center and reviewed by the DSMC. If the overall rate of incomplete TME is ≥ 26% (incomplete mesorectum in ≥ 26% specimens) and/or positive resection margins are reported in ≥ 26% specimens, the trial will be placed on hold and the interim results reviewed with the coordinating center.

• At each accruing study site, report of 2 cases with incomplete mesorectum and/or 2 cases with positive resection margins following taTME will prompt video auditing of the most recent taTME case resulting in incomplete TME, with assessment of the adequacy of taTME dissection by an independent reviewer blinded to the study site and surgeon. Any major deviation from recommended TME dissection principles will be addressed with the necessary corrective action plans. Report of a third case with incomplete mesorectum and/or positive resection margins, despite prior feedback and corrective action plan, will result in early stopping of the study at the study site.

**SAMPLE SIZE AND STATISTICAL ANALYSIS**

• The primary endpoint of the study is the rate of complete and near-complete mesorectal excision achieved by taTME, based on standard pathologic guidelines on pathology evaluation of TME specimens. The published average incidence of incomplete mesorectal excision with standard TME in large clinical series ranges from 10% to > 20%. The proportion of subjects in whom good quality mesorectal excision is achieved will be determined, which includes the proportion with complete and near-complete mesorectum. With a sample size of 100, the one-sided binomial test will reject the null hypothesis that the success rate is ≤ 80% if the experimental approach leads to efficacy of the total mesorectal excision for 87 or more subjects. This design achieves a power of 87% using one-sided binomial test for non-inferiority with 5% type 1 error assuming the true success rate is 90%.

• Secondary endpoints include (1) pathology assessment of the specimen, (2) 30-day perioperative complications, (3) long-term complications, (4) functional results (bowel function, urinary function, and sexual function), and (5) long-term oncologic outcomes (recurrence and survival). The overall morbidity and mortality rate of taTME will be calculated, as well as the incidence of specific types of complications. Three-year disease-free survival data will be obtained.

• Postoperative changes in bowel function and fecal continence scores (Wexner, FIQL, and COREFO), sexual function (FSFI or IIEF) and urinary function (IPSS), will be assessed by comparing functional scores obtained preoperatively and at 2 separate postoperative time points, using the respective validated instruments. A 50-75% compliance rate with filling the questionnaires is anticipated. Subjects will be excluded from a specific functional analysis if they complete less than 20% of questions from a given instrument.

• Kaplan-Meier method will be used to estimate overall survival (OS) and recurrence free survival (RFS) distribution functions with the log-rank test to compare distributions between subgroups of patients. Cumulative incidence functions (CIF) will be used to estimate time to locoregional recurrence and time to distant metastases in a competing risk setting. Univariable and multivariable hazard ratios for OS and RFS will be estimated using Cox proportional hazards models while hazard ratios for locoregional recurrence and distant metastases will be estimated using Fine and Gray's extension of Cox regression which models the hazards of the cumulative incidence function. All multivariable models will be used to adjust for confounding factors such as age, gender and race. Recurrence rates and postoperative complication rates will be estimated with a Poisson regression model with an offset for person-days followed. Mixed effects models with random effects or repeated measurements will be used to account for within-subject correlations arising from longitudinal data. Nonparametric methods (e.g., Wilcoxon rank sum test) will be applied for variables with non-normal distribution. Multiple imputation methods will be applied for missing data when appropriate.

## LIST OF ABBREVIATIONS

| | |
|---|---|
| AE | Adverse Event |
| AJCC | American Joint Committee on Cancer |
| APR | Abdominoperineal Resection |
| ASCRS | American Society of Colon and Rectal Surgeons |
| BMI | Body Mass Index |
| CBC | Complete Blood Count |
| CMP | Comprehensive Metabolic Panel |
| COREFO | Colorectal Functional Outcome |
| CR | Complete Response |
| CRM | Circumferential Resection Margin |
| CRT | Chemoradiation Therapy |
| CT | Computed Tomography |
| CTCAE | Common Terminology Criteria for Adverse Events |
| DRE | Digital Rectal Exam |
| DSMC | Data and Safety Monitoring Committee |
| ECOG | Eastern Cooperative Oncology Group |
| eCRF | Electronic Case Report Form |
| FIQL | Fecal Incontinence Quality of Life |
| FSFI | Female Sexual Function Index |
| H&P | History & Physical Exam |
| HRPP | Human Research Protections Program |
| IIEF | International Index of Erectile Function |
| IMA | Inferior Mesenteric Artery |

| | |
|---|---|
| IMV | Inferior Mesenteric Vein |
| IPSS | International Prostate Symptoms Score |
| ISR | Intersphincteric Resection |
| IV (or iv) | Intravenously |
| LAR | Low Anterior Resection |
| LR | Local Recurrence |
| MDT | Multidisciplinary Tumor Board Oncology Team |
| MRI | Magnetic Resonance Imaging |
| OS | Overall Survival |
| RFS | Recurrence Free Survival |
| SAE | Serious Adverse Event |
| SAGES | Society of Gastrointestinal and Endoscopic Surgeons |
| TAE | Transanal Local Excision |
| TAMIS | Transanal Minimally Invasive Surgery |
| TaTME | Transanal Total Mesorectal Excision |
| TEM | Transanal Endoscopic Microsurgery |
| TEO | Transanal Endoscopic Operation |
| TES | Transanal Endoscopic Surgery |
| TME | Total Mesorectal Excision |
| TSME | Tumor Specific Mesorectal Excision |
| WBC | White Blood Cells |

**TABLE OF CONTENTS**


STUDY SYNOPSIS .......... 2
LIST OF ABBREVIATIONS .......... 8
**1.** SUMMARY OF RESEARCH .......... 13
**2.** OBJECTIVES .......... 13
**2.1** Research Question .......... 13
**2.2** Study Design Overview .......... 13
**2.3** Hypothesis .......... 14
**2.4** Primary Objectives .......... 14
**2.5** Secondary Objectives .......... 14
**2.6** Study Endpoints .......... 14
**2.7** Power Analysis .......... 14
**3.** STUDY SCHEMA .......... 15
**4.** BACKGROUND .......... 15
**4.1** Principles of Rectal Cancer Treatment .......... 15
**4.1.1** Radical Surgical Resection for Rectal Cancer .......... 16
**4.1.2** Pathology Assessment of the TME Specimens .......... 17
**4.1.3** Local Excision for Rectal Cancer .......... 17
**4.2** Transanal Total Mesorectal Excision (taTME) .......... 18
**4.3** Study Rationale .......... 18
**4.4** Correlative Studies .......... 19
**5.** SUBJECT SELECTION .......... 21
**5.1** Sample Size .......... 21
**5.2** Inclusion Criteria .......... 21
**5.3** Exclusion Criteria .......... 22
**6.** STUDY CALENDAR .......... 23
**7.** STUDY DESIGN .......... 24
**7.1** Study Timeline .......... 24
**7.2** Endpoints .......... 24
**7.2.1** Primary Endpoints .......... 24
**7.2.2** Secondary Endpoints .......... 24
**7.3** Screening .......... 26
**7.4** Informed Consent .......... 27
**7.4.1** Stage I (T1/T2N0) and Subset of Stage II Rectal Cancer (T3N0) .......... 27
**7.4.2** Subset of Stage II and III Rectal Cancer (T3, any N) .......... 27
**7.5** Subject Enrollment .......... 29
**7.6** Subjects Not Receiving Neoadjuvant Treatment .......... 29
**7.7** Subjects Receiving Neoadjuvant Treatment .......... 29
**7.7.1** Preoperative Short-Course Radiation .......... 29
**7.7.2** Preoperative Chemotherapy or Chemoradiation .......... 30

**7.8** Subject Registration ... 30
**7.9** Preoperative Preparation ... 31
**7.10** Study Procedure ... 31
**7.10.1** Laparoscopic Access ... 31
**7.10.2** Transanal Access ... 31
**7.10.3** Specimen Removal and Anastomosis ... 32
**7.11** Pathology Assessment of the Surgical Specimen (APPENDIX B) ... 32
**7.12** Postoperative Care ... 33
**7.13** Postoperative Visits ... 33
**7.14** Postoperative Adjuvant Treatment ... 34
**7.15** Surveillance ... 34
**8.** DATA COLLECTION ... 34
**9.** DATA MANAGEMENT AND CONFIDENTIALITY ... 36
**9.1** Data Form Review ... 37
**9.1.1** Incomplete and Questionable Data ... 37
**9.1.2** Missing Forms ... 37
**9.2** Multicenter Protocol Confidentiality ... 37
**9.3** Record Retention ... 38
**9.4** Data Sharing ... 38
**10.** POTENTIAL BENEFITS AND RISKS TO HUMAN SUBJECTS ... 38
**10.1** Possible Benefits to Subjects ... 38
**10.2** Risks for taTME with Laparoscopic Assistance ... 38
**11.** ADMINISTRATIVE RESPONSIBILITIES ... 40
**11.1** Coordinating Center ... .40
**11.1.1** Public Disclosure of Clinical Trial ... 40
**11.1.2** Overall Principal Investigator's Responsibilities ... 41
**11.1.3** Coordinating Center's Responsibilities ... 41
**11.2** Participating Institutions ... 42
**11.2.1** Participating Institution Eligibility ... 42
**11.2.1.1** Rectal Cancer Volume ... 42
**11.2.1.2** Multidisciplinary Rectal Cancer Care ... 42
**11.2.1.3** Participating Surgeon's Experience ... 43
**11.2.1.4** Participating Surgeon's Prerequisite taTME Experience ... 43
**11.2.2** Study Staff ... 43
**11.2.3** Participating Institution's Responsibilities ... 43
**12.** DATA MONITORING ... 44
**12.1** Part I. Elements of Data and Safety Monitoring Plan ... 44
**12.1.1** External Study Monitor: BRANY (Biomedical Research Alliance of New York) ... 45
**12.1.2** Ongoing Monitoring of Protocol Compliance ... 45
**12.1.2.1** Site Initiation and Close-out Visit ... 45
**12.1.2.2** Registration Verification ... 46

**12.1.2.3** Ongoing Monitoring Activities ........ 46
**12.1.3** Ongoing Monitoring: Quality Control ........ 47
**12.1.3.1** Prerequisite Competency in Study Procedures ........ 47
**12.1.3.2** Monitoring of Study Procedures ........ 47
**12.1.3.3** Monitoring of Quality Assessment of TME specimens ........ 48
**12.1.3.4** Interim Analysis and Study Stopping Rule ........ 48
**12.1.3.5** Early Stopping Rule ........ 48
**12.1.4** Audit of Registration Process ........ 49
**12.1.5** Study Site Audits ........ 49
**12.1.5.1** Review of Audits ........ 49
**12.1.5.2** Corrective Actions ........ 50
**12.1.6** Procedures for AE and SAE Recording and Reporting ........ 50
**12.1.6.1** Adverse Event Characteristics ........ 50
**12.1.6.2** Serious Adverse Event Reporting ........ 51
**12.1.6.3** Protocol-specific Expedited Adverse Event Reporting Exclusions ........ 52
**12.1.6.4** Adverse Event Reporting ........ 52
**12.2** Part II. Data Safety Monitoring Committee/Data Safety Monitoring Board (DMC/DSMB) ........ 53
**13.** WITHDRAWAL OF SUBJECTS ........ 53
**14.** ECONOMIC IMPACT ON SUBJECTS ........ 54
**15.** PAYMENT TO SUBJECTS ........ 54
**16.** PROVISIONS FOR RESEARCH RELATED INJURY/HARM ........ 54
**17.** STATISTICAL ANALYSIS OF ENDPOINTS ........ 54
**17.1** Primary Endpoints ........ 54
**17.2** Secondary Endpoints ........ 54
**18.** SHARING RESULTS WITH SUBJECTS ........ 56
**19.** PROVISIONS TO PROTECT THE PRIVACY OF SUBJECTS ........ 56
**20.** VULNERABLE POPULATIONS ........ 57
**21.** NON-ENGLISH SPEAKING SUBJECTS ........ 57
**22.** REGULATORY REQUIREMENTS ........ 57
**22.1** Protocol Distribution ........ 57
**22.2** Protocol Revisions and Closures ........ 57
**22.3** Informed Consent Requirements ........ 58
**22.4** IRB Documentation ........ 58
**22.5** IRB Re-approval ........ 59
**22.6** Mount Sinai Hospital Multicenter Protocol Registration Policy ........ 59
**22.6.1** General Guidelines for Study Registration ........ 59
**22.6.2** Registration Process for the Coordinating Center ........ 59
**22.6.3** Eligibility Exceptions ........ 59
**22.6.4** Verification of Registration ........ 59
**22.6.5** Subject Registration Number ........ 60
**22.7** Protocol Deviations, Exceptions and Violations ........ 60

**22.7.1** Reporting Procedures ........ 60
**22.8** Safety Assessments and Toxicity Monitoring ........ 61
**22.9** Guidelines for Reporting Adverse Events ........ 61
**23.** MOUNT SINAI IRB REVIEW HISTORY ........ 62
REFERENCES ........ 64
Appendix A: Performance Status Criteria ........ 72
Appendix B: Pathology Template for Rectal TME Assessment ........ 73
PROTOCOL ADDENDUM (COVID 19 Contingency Plan) ........ 76

# 1. SUMMARY OF RESEARCH

Every year, 40,000 patients are diagnosed with rectal cancer in the US. While chemotherapy and radiation play an essential role in advanced disease, surgery is the cornerstone of curative treatment. Radical rectal cancer resection consists in total mesorectal excision (TME) which, despite the increasing use of laparoscopic and robotic approaches, remains associated with substantial morbidity related to postoperative infections, wound-related complications, defecatory, sexual and urinary dysfunction, as well as variable rates of TME completeness. Transanal TME (taTME) with laparoscopic assistance, was developed in an effort to facilitate completion of TME using a primarily transanal endoscopic approach. Performing TME transanally (using a "bottom-up approach") rather than trans-abdominally helps overcome major technical difficulties associated with low pelvic dissection. Published results from small taTME case series suggest the preliminary oncologic and procedural safety of this approach.

The current protocol is a 5-year phase II multicenter clinical trial to evaluate the efficacy and safety of a primarily transanal endoscopic approach with laparoscopic assistance to resect rectal cancer. The study is sponsored in part by Society of American Gastrointestinal and Endoscopic Surgeons (SAGES) and the American Society of Colon and Rectal Surgeons Research Foundation (ASCRS). A total of 100 participants with resectable rectal cancer located up to 10 cm from the anal verge will be enrolled across 11 US study sites and 1 Canadian site. All eligible participants will consist of patients who are candidates for laparoscopic or robotic TME with or without a temporary diverting ileostomy. The primary endpoint is efficacy of the TME, as defined by pathologic assessment of complete and near-complete mesorectal excision, based on standard guidelines on pathologic evaluation of TME specimens. Secondary endpoints include pathology assessment of the specimen, 30-day perioperative complications, long-term complications, changes in functional outcomes, and 3-year oncologic outcomes. Functional outcomes including bowel function, fecal continence, sexual and urinary function, will be assessed preoperatively and at various postoperative time points.

# 2. OBJECTIVES

## 2.1. Research Question

The objective of this phase II multicenter study is to evaluate the efficacy and safety of transanal total mesorectal excision with laparoscopic or robotic assistance in the management of resectable rectal cancer.

## 2.2. Study Design Overview

This is a phase II multicenter study to evaluate the non-inferiority and safety of transanal total mesorectal excision (taTME) with laparoscopic or robotic assistance to standard LAR. This study will be conducted in 100 subjects with preoperatively staged T1, T2 and T3 node positive and node negative rectal cancer located within 10 cm from the anal verge, who are eligible to undergo standard laparoscopic or robotic low anterior resection (LAR) with or without a temporary diverting loop ileostomy.

## 2.3. Hypothesis

The study's hypothesis is that the study procedure is non-inferior to standard LAR with respect to the quality of the mesorectal excision achieved.

The published incidence of incomplete mesorectal excision in standard LAR ranges from 10% or less to > 20% in more advanced stage rectal cancers, and tumors located within the distal third of the rectum. The hypothesis is supported by the overall PI's phase I study of taTME in 5 subjects (and first phase I study in the US) that demonstrated a 100% complete TME and the cumulative experience from published series in over 500 patients, reporting rates of incomplete mesorectal excision ranging from 0-12%.

## 2.4. Primary Objectives

To determine the efficacy of taTME as defined by pathology assessment of complete and near-complete mesorectal excision based on standard guidelines on pathologic evaluation of TME specimens (Appendix B).

## 2.5. Secondary Objectives

To perform (1) complete pathology assessment of the surgical specimen, (2) determine the incidence of 30-day perioperative complications including intraoperative and postoperative complications, (3) long-term (> 30 days) complications, (4) changes in functional results (bowel function, urinary function, and sexual function), and (5) assess 3-year oncologic outcomes (recurrence and survival).

## 2.6. Study Endpoints

The primary endpoint is the rate of complete and near-complete mesorectal excision achieved with transanal TME, based on standard guidelines on pathologic assessment of TME specimens.

The secondary endpoints include pathology assessment of the specimen, 30-day perioperative and long-term complications, postoperative functional outcomes, as well as long-term oncologic outcomes following transanal TME.

## 2.7. Power Analysis

We will determine whether the study procedure (taTME) is non-inferior to standard LAR using the proportion of subjects who reach efficacy of the TME, i.e. in whom good quality mesorectal excision is achieved (complete and near-complete mesorectum). With a sample size of 100, the one-sided binomial test will reject the null hypothesis that the success rate is ≤ 80% if the study procedure leads to efficacy of the total mesorectal excision for 87 or more subjects. This design achieves a power of 87%

using one-sided binomial test for non-inferiority with 5% type 1 error assuming the true success rate is 90%.

# 3. Study Schema

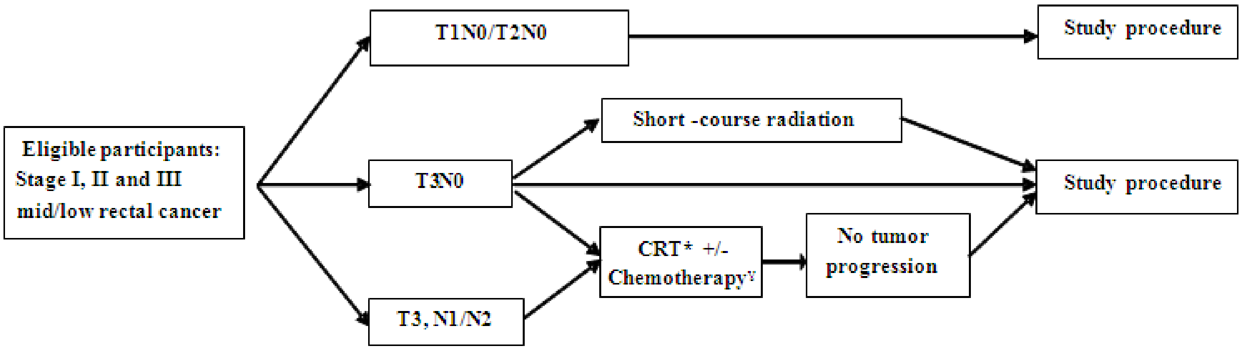


CRT*: Full-course preoperative chemoradiation
Chemotherapy¥: Induction or consolidation chemotherapy in addition to CRT

# 4. BACKGROUND

## 4.1. Principles of Rectal Cancer Treatment

The management of rectal cancer is multidisciplinary and outcomes are dependent on accurate preoperative staging, performance of a curative resection, and the selective use of neoadjuvant and adjuvant therapy to improve long-term oncologic outcomes. Preoperative staging of rectal cancer is achieved with CT or PET CT scans of the chest, abdomen and pelvis to assess metastatic spread and pelvic MRI to assess tumor size, mural spread (T stage), nodal involvement (N stage), and overall resectability with negative margins. For tumors of the mid and low rectum deemed resectable, the goals of a curative resection include transection of the IMA (inferior mesenteric artery) which is the main blood supply to the rectum and total mesorectal excision (TME) which consists in en-bloc removal of the rectum, mesorectum (the embryological lymphovascular package surrounding the rectum) and its covering (mesorectal fascia) down to pelvic floor with or without preservation of the anal sphincter.

For tumors of the upper rectum, tumor-specific mesorectal excision (TSME) followed by colorectal anastomosis is an acceptable alternative where the mesorectum is transected 5 cm distal to the lower margin of the tumor with equivalent oncologic outcomes as TME[1-3]. TSME allows for preservation of the lower rectum resulting in improved postoperative defecatory function and possible avoidance of a diverting stoma[1, 2]. Regardless of whether TSME or TME is performed, an adequate mesorectal specimen not only permits accurate pathologic staging, but its widespread adoption has resulted in a substantial decline in local recurrence rates over the last 2 decades.

### *4.1.1. Radical Surgical Resection for Rectal Cancer*

Radical rectal cancer resection with TME is achieved with abdominoperineal resection (APR) or low anterior resection (LAR). APR is en-bloc removal of the rectum and anus, committing the patient to life with a permanent colostomy and is usually reserved for very low rectal tumors (i.e. within 2-4 cm of the anal verge and/or invading into the anal sphincter complex precluding preservation of continence). LAR on the other hand preserves the anus and anal sphincters with reconnection of the proximal colon to the anus with or without creation of a coloanal or colonic J pouch using sutures (handsewn) or a stapler (stapled anastomosis).

The standard of care for locally advanced rectal cancer (T3/T4 and/or N1/N2) consists of preoperative chemoradiation (CRT) followed by radical resection and postoperative chemotherapy. Relative to postoperative CRT, preoperative CRT results in lower toxicity, lower local recurrence and higher rates of sphincter preservation[4-6]. In addition, preoperative CRT is associated with a 10-30% rate of complete tumor regression and downstages/downsizes up to 40-60% of rectal tumors which can often permit sphincter-preserving resection[6,7]. Recent trials have demonstrated that preoperative short-course radiation is an oncologically adequate alternative to full-course CRT for locally advanced disease, with equivalent local recurrence rates. Short-course radiotherapy is increasingly being used in an effort to minimize the toxicity associated with long-course CRT[8, 9].

The cumulative morbidity of CRT and radical cancer resection is substantial. In addition to significant gastrointestinal and neurologic side effects from CRT, LAR and APR are associated with 2-8% mortality and 30-40% incidence of perioperative complications[3, 10]. Anastomotic leakage following LAR can progress to pelvic sepsis requiring reoperation and creation of a temporary diverting stoma, if not already created. The incidence of anastomotic leaks following rectal cancer resection ranges from 10 to 25% with risk factors including tumor location in the lower third of the rectum and preoperative CRT[11, 12]. A diverting stoma is performed in the vast majority of patients undergoing TME for tumors in the mid and low rectum and/or who have received full-course preoperative CRT in order to minimize the chance of pelvic sepsis from anastomotic leak. Stoma closure is typically performed 2-3 months later to allow for healing of the anastomosis or following completion of postoperative chemotherapy. Despite the widespread teaching and adoption of TME for rectal cancer, the incidence of incomplete mesorectal excision (positive distal or circumferential radial margin or incomplete mesorectum) remains higher in higher T stage tumors and for very low rectal tumors. Large clinical series evaluating outcomes following TME have reported rates of incomplete TME > 20% in some series with T3/T4 rectal cancer and tumors located ≤ 5cm from the anal verge, as opposed to rates of 5-10% for more favorable tumors (T1/T2 tumors located higher in the rectum)[13-17].

Given the morbidity of rectal cancer treatment, particularly in elderly patients, there is significant interest in less invasive surgical alternatives. Through the use of smaller skin incisions, laparoscopy for colon cancer was shown to result in less pain, shorter length of hospital stay and faster recovery than conventional open surgery but equivalent perioperative complications and long-term oncologic outcomes[18-20]. Several randomized trials have compared laparoscopic to open radical resections with TME for rectal cancer and shown no differences in short-term oncologic outcomes. While laparoscopic LAR resulted in faster recovery and shorter hospital stay than open LAR, there were no statistically significant differences in perioperative outcomes[3, 15, 21-23].

### *4.1.2. Pathology Assessment of TME Specimens*

Rectal cancer staging is performed in accordance with the American Joint Committee on Cancer (AJCC) 7$^{th}$ Edition TNM classification. The pathology assessment of TME specimens is based on standard guidelines on pathologic evaluation of TME specimens, which include grading of the mesorectum with macroscopic assessment of the completeness of the mesorectum. Additional pathologic evaluation includes TNM staging, assessment of surgical margins, lymph nodes, and other histopathologic tumor features such as differentiation and tumor regression grade (Appendix B) [13, 24-26]. Good quality mesorectal excision is defined as the pathological assessment of a complete mesorectum (smooth surface of mesorectal fascia with the entire mesorectal envelope present and no defect deeper than 5mm), or near-complete mesorectum (intact mesorectal envelope except for small irregularities and/or defects greater than 5 mm but that do not extend to the muscularis propria. In contrast, an incomplete TME specimen demonstrates deeper defects in the mesorectum with exposed muscularis propria and/or very irregular circumference[13, 24-26]. The mesorectal grade of TME specimens has been shown to be an independent predictor of local recurrence and disease-free survival[13-16, 24-26] and is a more accurate marker of the quality of the mesorectal excision. Although the CRM is an important predictor of local recurrence and distant disease, CRM positivity doesn't necessarily reflect the quality of the surgery since TNM stage, tumor size, low tumor position, tumor differentiation and invasive growth pattern have all been shown to be associated with tumor positivity[26].

### *4.1.3. Local Excision for Rectal Cancer*

By avoiding abdominal incisions, transanal local excision (TAE) represents the least invasive approach to rectal cancer. TAE consists of resection if the lesion through the entire thickness of the rectal wall followed by closure of the defect with sutures. Transanal Endoscopic Surgery (TES) is a variation of TAE where rectal lesions are resected under endoscopic visualization with excellent results. TES is routinely performed using rigid or flexible transanal endoscopic transanal platforms equipped with $CO_2$ for rectal insufflation, a magnifying camera and adapted instruments are inserted transanally to perform full-thickness rectal wall excision.

By providing magnified optics, TES permits a more precise dissection than TAE. Overall, TAE and TES for rectal cancer are associated with negligible mortality and significantly lower morbidity than radical resections[27-31]. Complications include urinary retention, suture line breakdown, bleeding, and fecal incontinence[27-31]. In long-term functional studies, fecal incontinence occurred in < 5% of TES cases[27] but was transient in nearly all cases[27, 31]. TAE and TES are limited by the fact that the mesorectum and mesorectal lymph nodes are not removed with this approach, potentially under-staging and under-treating more advanced rectal tumors. Because there is a higher incidence of positive lymph nodes in lesions of advanced T stage, local excision is associated with higher recurrence rates than radical resection for locally advanced rectal tumors. Therefore, local excision with curative intent is reserved for premalignant polyps and carefully selected early rectal cancer (T1) with lowest risk of positive lymph nodes, while high-risk T1 rectal cancers and more advanced rectal tumors should be treated with radical surgery.

### 4.2. Transanal Total Mesorectal Excision (taTME)

Rectosigmoid resection with TME using a pure or primarily transanal endoscopic approach was demonstrated to be feasible and safe in experimental models[32-34]. This procedure rapidly and successfully transitioned to clinical application with a growing number of case series describing this approach in selected patients with rectal cancer[35-51]. Transanal or perineal rectosigmoid resection is not entirely novel in that it is used in the management of selected cases of rectal cancer. For very low rectal tumors located 0 to 4 cm from the anal verge that would traditionally require APR, sphincter-preserving transanal intersphincteric resection is a technique that has been used since 1994. Open or laparoscopic rectal dissection with TME is performed in the standard fashion followed by transanal dissection of the internal sphincter (either in part or completely) in continuity with the rectum, with preservation of the external sphincter. The specimen is then pulled out through the anus, transected, followed by standard coloanal handsewn or stapled anastomosis with creation of a diverting stoma. This approach increases the chance of achieving a negative distal resection margin with preservation of continence and good long-term oncologic[48-55] and acceptable functional outcomes[56, 57]. Transanal TME builds on the same principles as perineal intersphincteric resection, but extends rectal and mesorectal dissection through the use of transanal endoscopic platforms, dissection tools and $CO_2$ insufflation.

### 4.3. Study Rationale

Based on the preliminary results from several published case series on this technique[35-47], in carefully selected patients with resectable rectal cancer, taTME with laparoscopic assistance is safe with respect to achieving an adequate oncologic resection and avoids the need for large abdominal incisions and related wound complications. It is anticipated that this larger, phase II multicenter study will further demonstrate the safety of this approach with respect to perioperative outcomes, short and long-term oncologic outcomes and functional results.

### 4.4. Correlative Studies

More than seventeen series of transanal TME with laparoscopic assistance have been published since the first case of laparoscopic-assisted transanal TME was published in 2010[35] (total N=592, range 5-140 patients per study) [36-51]. Across these published series, 7% of taTME cases were performed as part of an APR, and 93% as part of sphincter-preserving restorative proctectomy. The majority of tumors were located in the mid- and low rectum (<10 cm from the anal verge), with only four studies including high rectal tumors (≥ 10 cm from the anal verge) [36,41,46,47].

With respect to tumor selection for taTME, the vast majority of studies performed taTME for non-obstructing tumors including preoperatively staged T1, T2, and T3, N0 or N1 tumors. When studies were performed under IRB, and early in their operative experience, most authors specifically excluded T4 and metastatic tumors, local recurrences, and tumors with threatened circumferential radial margins (CRM) based on staging MRI[36, 38]. Other exclusion criteria included recurrent tumors and patients with extensive abdominal or pelvic surgery. The majority of patients with locally advanced rectal tumors received preoperative CRT with creation of a protective loop ileostomy, except in patients with upper rectal tumors.

Cumulatively, the mesorectum was complete in 90% and near-complete in 8.5% of patients, with negative resection margins achieved in 90-100%, and an average of 10-33 lymph nodes harvested. Although 6 publications included 1-2 unsuspected T4 rectal tumors[38,39,42,44,45,51], only one study specifically selected unfavorable tumors in male patients for this approach, including large T3 and T4 tumors, located anteriorly, in the distal 5 cm of rectum, and with threatened positive CRMs[44], all radiated preoperatively. The rationale for this patient selection was to facilitate completion of sphincter-preserving good-quality TME in cases that were otherwise predicted to be technically challenging and associated with a high risk of incomplete mesorectal specimens. The authors were able to achieve a complete mesorectum in every case, but reported a 13% incidence of positive margins, and 80.5% overall survival at 24 months, reflecting the advanced stage of the tumors[44].

Based on the 17 published series of pure and hybrid taTME for rectal cancer, the cumulative intraoperative complication rate was 7% (44/592 cases) and mostly consisted of conversions to open proctectomy (N=18) due to technical difficulties during transanal dissection. Other intraoperative complications included 4 urethral injuries (0.6%), one case of air embolism, 5 rectal perforations (8%), and 6 cases of delayed anastomosis due to technical difficulties (1%). Ten percent of all reported intraoperative complications (5/44 cases), including 50% of urethral injuries, documented with taTME occurred in the Rouanet study, which was not entirely surprising given selection of high-risk patients, including males with very low, bulky, and mostly anterior tumors[44]. The authors pointed out that the 2 urethral injuries occurred early in their learning curve and during dissection of bulky anterior tumors[44].

The incidence of postoperative complications based on the 17 published case series, is within the range of that anticipated from laparoscopic TME, and ranges from 0-40%. Only one 30-day mortality was noted from medical complications[49]. Major complications included anastomotic leak, intra-abdominal abscess, sepsis, SBO, bleeding, ileus, and transient urinary retention. In the only comparative matched series of taTME to laparoscopic TME that evaluated early oncologic as well as perioperative outcomes, there were no statistically significant differences in complication rates between the groups (32% vs. 51%) [46].

In a large, multicenter taTME series published , 56 patients with locally advanced tumors ≤ 5 cm from the anal verge, most of which were treated with neoadjuvant treatment (84%), underwent taTME with laparoscopic assistance with complete or near-complete mesorectum achieved in all cases and a 95% R0 resection rate[43]. There were 3 conversions and 6 cases of delayed anastomosis, no mortality, and a 26% morbidity rate including anastomotic leakage, pelvic sepsis, urinary dysfunction, bleeding, and a cerebrovascular accident. Local recurrence rate at a median follow-up of 29 months was 1.7%, with 96.4% overall survival[43]. Data from the first international taTME registry has been recently published. The study analyzed data from 66 registered units in 23 countries. The primary endpoint was "good-quality TME surgery". Secondary endpoints were short-term adverse events. A total of 720 consecutively registered cases were analyzed, comprising of 634 patients with rectal cancer. Overall conversions were 8.3 %. Complete and near-complete TME specimens were achieved in 85% and 11%, respectively, with incomplete TME in 4%. R1 resection rate was 2.7%. Postoperative mortality and morbidity were 0.5% and 32.6%, respectively. The authors concluded that taTME appears to be an oncologically safe and effective technique for distal mesorectal dissection with acceptable short-term patient outcomes and good specimen quality.[58]

Although the international experience with taTME is still preliminary with no randomized trial yet comparing taTME with open or laparoscopic TME, two retrospective studies compared outcomes of matched cohorts of patients who underwent taTME vs. laparoscopic TME[46,47]. Fernandez-Hevia et al. retrospectively case-matched 37 cases of laparoscopic-assisted taTME with 37 cases of laparoscopic TME for rectal cancer, and demonstrated no significant differences with respect to quality of the mesorectal specimen, lymph node harvest, resection margins, or intraoperative complications[46]. They also demonstrated comparable 30-day postoperative complications, but a statistically significant lower readmission rate in the taTME group (2% vs. 6%) [46]. Velthuis et al. retrospectively matched 25 cases of laparoscopic-assisted taTME with 25 cases of laparoscopic TME and interestingly, they found that taTME was associated with a significantly higher rate of complete mesorectum than laparoscopic TME (92% vs. 72%) [47].

In conclusion, the published short-term outcomes of taTME with laparoscopic assistance suggest the preliminary oncologic and overall safety of this approach in the

management of resectable rectal cancer. While the published reports are promising, they also highlight the need for careful patient selection, and the need for larger prospective trials with functional outcomes and longer-term oncologic outcomes of this approach. While taTME is becoming widely used worldwide and in the US, currently published data highlights the paucity of well-designed large multicenter trials evaluating perioperative, oncologic and functional outcomes of this procedure.

# 5. SUBJECT SELECTION

## 5.1. Sample Size

We anticipate pre-screening of 20-100 rectal cancer patients per year per study site, based on the yearly volume of referrals at each institution. The study total sample size is 100 subjects to undergo laparoscopic or robotic LAR with taTME over a 3-year accrual period across 12 study sites. Accrual of at least 5 patients per site per year is anticipated. Accounting for a screen failure rate of 30-40% we anticipate that approximately 140 patients will be enrolled and 100 will be registered and undergo the laparoscopic or robotic LAR with taTME. The 12 study sites will be allowed competitive enrollment. The study duration will be 5 years; 3 years of accrual, and 2 to 5 years of long-term oncologic follow-up for the last participant enrolled.

## 5.2. Inclusion Criteria

Assessment to verify eligibility must be completed within 14 days prior to registration. Participants must meet the following criteria on screening examination to be eligible to participate in the study:

- New diagnosis of histologically confirmed adenocarcinoma of the rectum
- Males and females 18 years of age or older, because rectal cancer is rare in pediatric patients in the absence of hereditary mutations
- Rectal cancer originally staged as T1, T2, or T3, node negative (N0) or node positive (N1, N2) by pelvic MRI
- ECOG performance status ≤ 2 (Karnofsky ≥ 60%, see Appendix B)
- Circumferential radial margin not involved with tumor on pelvic MRI
- No evidence of metastasis on staging CT or PET CT scans of the chest, abdomen and pelvis

- Rectal cancer located within 10 cm from the anal verge based on proctoscopy and DRE

- Complete preoperative colonoscopy demonstrating no synchronous colon cancer

- Participants must be eligible to undergo laparoscopic or robotic low anterior resection with or without a temporary diverting stoma, based on multidisciplinary tumor board consensus
- Ability to understand and the willingness to sign a written informed consent document

**5.3. Exclusion Criteria**

Participants who exhibit any of the following conditions at screening will not be eligible for admission into the study:

- Rectal cancer staged as T4 by pelvic MRI

- Participants with preoperatively staged T3, N1, or N2 tumors who return > 12 weeks following completion of neoadjuvant treatment

- Severely symptomatic rectal tumors (near-completely obstructing, symptomatic bleeding)

- Tumors invading into the internal anal sphincter muscle based on DRE and pelvic MRI

- Fecal incontinence at baseline

- Prior history of rectal resection

- Prior history of colorectal cancer

- History of inflammatory bowel disease

- Uncontrolled concurrent illness including, but not limited to ongoing or active infection, symptomatic congestive heart failure, unstable angina pectoris, cardiac arrhythmia, or psychiatric illness/social situations that would limit compliance with study requirements

- Pregnant women are excluded from this study because chemotherapy and/or radiation which may be required for rectal cancer treatment have the potential for teratogenic or abortifacient effects. Because there is an unknown but potential risk of adverse events in nursing infants secondary to treatment of the mother with chemotherapy and/or radiation,

breastfeeding should be discontinued if the mother is treated with chemotherapy and/or radiation

- Subjects who cannot read or understand English

## 6. STUDY CALENDAR

| | Prior to registration | Prior to neoadjuvant Tx (if applicable) | | Preop | | Postop | | | | | | |
|---|---|---|---|---|---|---|---|---|---|---|---|---|
| | | | | | | Wk 1-2 | Wk 4-6 | Year 1 | Year 2 | Year 3 | Year 4 | Year 5 |
| ICF | X | | REGISTRATION | | SURGERY | | | | | | | |
| Surgical Consent for study procedure | | | | X | | | | | | | | |
| Clinical Evaluation (BMI, DRE, distance from anal verge) | X | | | X[a] | | | | | | | | |
| Proctoscopy | X | | | | | | | Q 3-6 months | | Q 6 months | | |
| H&P | X | | | X[b] | | X | X | Q 3-6 months | | Q 6 months | | |
| CEA | X | | | | | | | Q 3-6 months | | Q 6 months | | |
| Colonoscopy | X | | | | | | | X | | | X | |
| CT (chest, abdomen and pelvis) | X[c] | | | | | | | Q 6-12 months | | | | |
| Pelvic MRI | X[c] | | | | | | | | | X | | |
| Performance Status | X | | | | | | | | | | | |
| Labs[d] | X | | | X[b] | | X | | | | | | |
| Operative Report | | | | | | X | | | | | | |
| Histopathology | X | | | | | | X | | | | | |
| Postoperative Complications | | | | | | X | X | Q 4-6 months | | | | |
| Oncologic FU | | | | | | | | Q 3-6 months | | Q 6 months | | |
| Questionnaires[e] | | X[e1] | | X[e2] | | | | X[e3] | X[e4] | | | |
| AE and SAE evaluation | | | | | From index surgery throughout the rest of the study | | | | | | | |

a. The tumor may be reassessed post-neoadjuvant treatment to reconfirm eligibility.
b. Repeat Labs, H&P and vitals preoperatively if pre-registration labs/H&P/Vitals were performed more than 30 days prior to surgery.
c. Imaging, colonoscopy and rectal tumor biopsy performed up to 3 months prior to screening (or prior to start of neoadjuvant treatment, in subjects enrolled during or at completion of neoadjuvant treatment) can be used to determine eligibility.
d. CBC, PT/PTT/INR, sodium, potassium, chloride, CO2, BUN, creatinine, glucose, albumin, and pregnancy test (in patients with childbearing potential)
e. Questionnaires (Wexner, FIQL, COREFO, IPSS, IIEF, or FSFI) are administered prior to start of neoadjuvant treatment, if applicable (e1); before study procedure or following completion of neoadjuvant treatment (e2); 6-8 months postoperatively in non diverted subjects or 3-4 months following ileostomy closure (e3); 12-18 months following study procedure (e4)

# 7. STUDY DESIGN

## 7.1. Study Timeline

The research study will be conducted for 5 years with an accrual period of 3 years. Participants will be followed for up to 5 years following the study procedure to assess long-term oncologic outcomes. Out-of-state participants will be contacted periodically by the research team to collect data from surveillance studies. The estimated study completion date is 5 years following the start of enrollment. Preliminary analyses will be performed following accrual period, and final analyses will be performed approximately 1 year following completion of accrual.

## 7.2 Endpoints

### *7.2.1. Primary Endpoints*

The primary endpoint is the rate of complete and near-complete mesorectal excision achieved with taTME, based on standard guidelines on pathology assessment of TME specimens[24-26, 59]. The quality of the mesorectal excision will be graded as:

- Complete
- Near-complete
- Incomplete

The secondary endpoints include pathology assessment of the specimen, 30-day perioperative and long-term complications, postoperative functional outcomes, as well as long-term oncologic outcomes following transanal TME.

### *7.2.2. Secondary Endpoints*

- Pathology assessment of the surgical specimen (synoptic report, Appendix B)
    - Distal resection margins
    - Circumferential radial margins
    - Number of lymph nodes analyzed
    - TNM stage
    - Other histopathologic tumor features
- 30-day perioperative complications

- Intraoperative complications
    - Conversion to open surgery
    - Bleeding (blood loss > 750ml)

    - Bowel perforation
    - Organ injury

- Surgical postoperative complications
    - Ileus
    - Bowel obstruction
    - SSI (surgical site infection)
    - Abdominal or pelvic abscess (diagnosis based on radiologic findings)
    - Bleeding requiring ≥ 4 units of blood transfusion and/or reoperation
    - Incisional hernia
    - Re-operation
    - Anastomotic leak (diagnosis based on radiologic findings and/or intraoperative findings)
    - Sepsis

- Medical postoperative complications
    - Readmission
    - Urinary retention
    - Urinary tract infection
    - Dehydration
    - Pneumonia
    - Deep vein thrombosis
    - Renal failure
    - Cardiopulmonary failure
    - Pulmonary embolism

- Long-term postoperative complications
    - Incisional hernia
    - Anastomotic stricture
    - Colovaginal fistula

    - Sexual dysfunction (that persists beyond 6 months postoperatively or beyond 3 months following ileostomy closure)
    - Fecal incontinence (that persists beyond 6 months postoperatively or beyond 3 months following ileostomy closure)
    - Poor bowel function (that persists beyond 6 months postoperatively or beyond 3 months following ileostomy closure)
    - Urinary dysfunction (that persists beyond 6 months postoperatively or beyond 3 months following ileostomy closure)

- Oncologic outcomes
    - Locoregional recurrence (defined as any recurrence diagnosed in the pelvis i.e. tumor bed, pelvic nodes, anastomosis, drain site, or perineum, occurring alone or with other sites of recurrence)[55]
    - Distant metastases (cancer recurrence occurring outside the pelvis)

### 7.3. Screening

Upon referral to the study site for management of newly diagnosed rectal cancer, potential study participants will be screened for eligibility. Subjects' medical records, imaging studies, endoscopy and pathology reports will be reviewed at the institution's multidisciplinary tumor board conference that includes specialists in Gastrointestinal Surgery, Medical Oncology, Radiation Oncology, Pathology, and Radiology. Colonoscopy, rectal tumor biopsy, CT and/or PET CT and MRI imaging studies performed within 3 months of study screening (or within 3 months of start of neoadjuvant treatment, in cases enrolled during or after completion of neoadjuvant treatment) can be used to determine patient eligibility. . Subjects with preoperatively staged T1, T2 and T3, node positive or negative rectal adenocarcinoma located within 10 cm from the anal verge, with no evidence of metastasis, and who are eligible to undergo elective sphincter-preserving laparoscopic or robotic LAR, will be identified as potential study participants.

At the initial screening visit, standard rectal cancer evaluation will be performed which consists of history and physical examination, DRE, and proctoscopy. Subjects with preoperatively staged T3, N1 and N2 tumors (stage II and III) based on initial pelvic MRI will also be evaluated by medical and radiation oncology for consideration for neoadjuvant therapy.

## 7.4. Informed Consent

Subjects who are candidates for elective laparoscopic or robotic LAR with TME without or without creation of a temporary diverting stoma, who meet study inclusion criteria and have no exclusionary criteria, will be made aware of the study by their primary surgeon or other caretakers. If interested to hear more about the study, a thorough description of the study procedures, and alternatives (laparoscopic, robotic, and open LAR) with TME including risks and benefits of each approach, will be provided by one of the study surgeons who is not the participant's primary surgeon. It will be made clear to the subject that regardless of the choice of procedure, a temporary diverting stoma (loop ileostomy) may be performed based on the surgeon's preference given specific tumor characteristics. A copy of the informed consent will be provided for the subject to take home and review with their family and/or physician. All subjects will be informed that their choice of procedure will in no way affect their care at this institution and that participation is entirely voluntary.

Informed consent for research should be obtained in compliance with standard GCP guidelines.

### *7.4.1. Stage I (T1/T2N0) and Subset of Stage II Rectal Cancer (T3N0)*

Subjects with stage I rectal cancer and a subset of subjects with T3N0 rectal cancer determined not to require neoadjuvant treatment based on the recommendations by the study site's MDT (multidisciplinary tumor board oncology team), will proceed directly to surgical resection with TME. If the subject is interested in enrolling in the study, the study surgeon will review the case with the study site PI to determine final eligibility. The subject will be contacted by a member of the research team 48-72 hours following initial visit to confirm final eligibility and determine participation. If additional preoperative studies and/or additional review of the case with members of the MDT are required prior to determining final eligibility, the subject will be contacted by a member of the research team once these studies have been reviewed and a consensus has been reached regarding eligibility. If the subject is eligible and wishes to participate in the study, he/she will return to the office for a final review of the protocol and study procedure with a study staff member who is not the participant's primary surgeon, and who will obtain consent for enrollment in the study. If the patient is eligible to proceed directly to taTME, surgical consent for the study procedure will also be obtained.

### *7.4.2. Subset of Stage II and III Rectal Cancer (T3, any N)*

Based on the recommendations of the MDT at the study site, most subjects with T3 and/or suspicious nodes based on pelvic MRI will be offered to undergo either short-course preoperative radiation therapy followed by surgery or long-course preoperative CRT, with or without induction or consolidation chemotherapy[62], followed by surgery 6-12 weeks later as per standard of care.

Subjects for whom short-course preoperative radiation therapy is planned, and who meet eligibility criteria for the study and are interested in participating in the study, will receive a copy of the informed consent to take home and review with family

and/or physician. The subject will be contacted by a member of the research team 48-72 hours following initial visit to confirm final eligibility and determine participation. The subject will return to the office for a final review of the protocol and study procedure with a study staff member who is not the participant's primary surgeon. Consent for enrollment in the study as well as surgical consent will be obtained.

Eligible subjects for whom preoperative CRT is planned (with or without induction or consolidation chemotherapy), and who are interested in participating in the study, will receive a copy of the informed consent to take home and review with their family and/or physician. The subject will be contacted by a member of the research team 48-72 hours following initial visit to confirm final eligibility and determine participation. Subjects will return to the office to review the protocol and study procedure with a study staff member who is not the participant's primary surgeon, and consent for study participation will be obtained. Standard chemoradiation will be administered as per routine local protocols. Induction or consolidation chemotherapy may be added to standard CRT[62], as per MDT recommendations. Treatment doses may be reduced based on toxicity and treatment may be interrupted if poorly tolerated. Subjects will return to the office following completion of neoadjuvant therapy for standard clinical re-evaluation. If there is no evidence of local tumor progression on DRE and/or proctoscopy, if there has been no change in eligibility criteria, and if the subject still wishes to participate in the study, the study procedure will be reviewed again in detail by a member of the research team, and the surgical consent will be obtained by one of the study members who is not the subject's primary surgeon. If there is evidence of local tumor progression on DRE and/or proctoscopy, and/or if the patient no longer meets eligibility criteria, he/she will be removed from the study and offered standard treatment.

In addition, subjects undergoing or already having completed neoadjuvant treatment for preoperatively staged II and III rectal cancer will also be identified and screened as potential study participants. Eligibility will be determined by reviewing medical records, preneoadjuvant staging imaging, endoscopy and pathology reports, initial clinical evaluation performed and confirmed by the study staff including tumor assessment by digital rectal examination and proctoscopy. When subjects return for routine clinical re-evaluation, if there is no evidence of tumor progression on DRE and/or proctoscopy, and if the subject meets study eligibility, one of the study surgeons will provide a thorough description of the study procedure and alternative, as described above. A copy of the informed consent will be provided to take home and review with family and/or physician. The study surgeon will review the case with the site PI and other study staff as needed to determine final eligibility. Subjects will be contacted by the research coordinator 48-72 hours later to determine participation. If the subject wishes to participate in the study, he/she will be asked to return for a final review of the protocol and study procedure with a member of the research team. Consent for

study enrollment and the surgical consent will be obtained by a member of the study team.

### 7.5. Subject Enrollment

All subjects enrolled in the study will be assigned an enrollment number on the Enrollment log starting with "EN-" followed by a 4-digit number, where the first 2 numbers designate the study site, and the last 2 numbers designate the subject number (example, EN-01-05).

### 7.6. Subjects Not Receiving Neoadjuvant Treatment

For subjects with stage I rectal cancer and a subset of subjects with T3N0 rectal cancer determined not to require neoadjuvant treatment, standard preoperative evaluation and testing will be performed as indicated based on comorbidities. Following enrollment in the study, participants will be asked to complete questionnaires to assess baseline preoperative bowel function including the Wexner score[63], the Colorectal Functional Outcome questionnaire[64] (COREFO), the Fecal Incontinence Quality of Life questionnaires[65] (FIQL). They will also be asked to complete the Female Sexual Function Index[66] (FSFI) or International Index of Erectile Function questionnaires[67] (IIEF) to assess baseline sexual function, and the International Prostate Symptom Score questionnaire[68] (IPSS) to assess baseline urinary function. Subjects will be given the choice to either answer these questionnaires electronically or on paper form at the time of an office visit.

### 7.7. Subjects Receiving Neoadjuvant Treatment

Subjects with preoperatively staged N1 or N2 rectal cancer and subjects with T3N0 rectal cancer determined to require neoadjuvant treatment, will be offered either short-course radiation therapy or long-course preoperative CRT (with or without induction or consolidation chemotherapy), based on MDT recommendation. If participants choose to receive neoadjuvant treatment at another institution closer to their home, care will be coordinated by the oncology team at the participating institution. Following enrollment in the study, participants will be asked to complete questionnaires prior to the start of neoadjuvant treatment in order to assess baseline preoperative bowel function Wexner score[63], COREFO[64], FIQL[65], and FSFI[66] or IIEF[67] and IPSS[68].

#### *7.7.1. Preoperative Short-Course Radiation*

Study subjects determined to be eligible for short-course preoperative radiation therapy will undergo standard preoperative evaluation and testing as indicated based on comorbidities and the study procedure will be scheduled within 2 weeks of completion of radiation. Study participants will be asked to complete questionnaires prior to the start of radiation therapy to assess baseline preoperative bowel function (Wexner score, COREFO, FIQL), sexual function (FSFI or IIEF) and urinary function (IPSS). Participants will be asked to complete the same

questionnaires again following completion of short-course radiation therapy and prior to surgery in order to assess post-radiation bowel function.

#### *7.7.2. Preoperative Chemotherapy or Chemoradiation*

Study subjects receiving preoperative CRT (with or without induction or consolidation chemotherapy) will return following completion of neoadjuvant treatment for repeat clinical examination which will include physical examination, DRE and proctoscopy. This is routinely performed prior to rectal cancer surgery to assess clinical tumor response following neoadjuvant treatment. If there is suspicion of local tumor progression on DRE and proctoscopy, (increase in size or fixity), patients will be re-staged by pelvic MRI as is standard of care.  If the tumor is upstaged to T4 or metastatic disease, the patient will be removed from the study and be offered standard of care treatment. If the participant still meets inclusion criteria, he/she will be maintained in the study and surgical consent for the study procedure will be obtained. Standard preoperative evaluation and testing will be performed as indicated based on comorbidities. In addition, study participants will be asked to complete questionnaires following completion of CRT and prior to surgery to assess post-CRT bowel function (Wexner score, COREFO, FIQL), sexual function (FSFI or IIEF) and urinary function (IPSS).

In addition, subjects already undergoing or having already completed neoadjuvant treatment for preoperative staged II or III rectal cancer will be screened as potential study participants. When they return after completion of neoadjuvant treatment for routine clinical re-evaluation including DRE and proctoscopy, if there is no evidence of tumor progression on DRE and proctoscopy, and if the participants meet study eligibility and wish to enroll in the study, a copy of the informed consent will be provided to take home and review with family and/or physician. If the participant wishes to participate in the study, he/she will be asked to return for a final review of the procedure with a member of the research team at which time the consent will be obtained by one of the study team members. Standard preoperative evaluation and testing will be performed as indicated based on comorbidities. In addition, participants will be asked to complete post-CRT bowel function (Wexner score, COREFO, and FIQL), sexual function (FSFI or IIEF) and urinary function (IPSS) questionnaires.

### 7.8. Subject Registration

After confirming the study eligibility of subjects previously enrolled in the protocol and the surgical consent for the study procedure signed, the participant will be registered for the study. Participants must be registered with the coordinating center's study coordinator before undergoing the study procedure.  The study procedure may not be performed until the participating institution receives a faxed or e-mailed copy of the participant's registration confirmation memo from the coordinating center.

Upon confirmation of registration by the coordinating center, the participant will be assigned a 4-digit Subject Registration Number starting with "R-". The first 2 digits of

the Subject Registration Number refer to the study site and the last 2 digits refer to the subject number (example, R-02-04).

## 7.9. Preoperative Preparation

The study procedure will be scheduled to take place within 2 weeks of completion of short-course preoperative radiation therapy or 6-12 weeks following completion of neoadjuvant treatment as is standard of care. Standard medical clearance will be obtained preoperatively. Standard preoperative testing will be performed including blood work (CBC, electrolytes, carcinoembryonic antigen (CEA) level, coagulation function, and albumin). Participants will undergo standard mechanical bowel preparation and receive standard perioperative antibiotic prophylaxis as per routine. All patients will undergo standard preoperative teaching and stoma site marking in preparation for possible fecal diversion.

## 7.10. Study Procedure

### *7.10.1. Laparoscopic Access*

Laparoscopic access will be obtained by the abdominal surgeon and the abdomen will be insufflated with $CO_2$ in the standard fashion. The abdominal surgeon will survey the abdomen and pelvis to assess for the presence of any findings precluding safe transanal access. Dissection of the inferior mesentery artery (IMA) and inferior mesentery vein (IMV) pedicle will be performed laparoscopically or robotically either prior to, during, or following transanal dissection. Unless the sigmoid and left colon are noted to be already very redundant, the splenic flexure will be taken down laparoscopically or robotically, as is routine in rectal cancer resections.

### *7.10.2. Transanal Access*

After localizing the rectal tumor transanally through a proctoscope, the transanal surgeon will place a pursestring suture ≥ 1cm below the lower edge of the tumor to close the rectum. The transanal endoscopic platform will be inserted transanally and sealed and $CO_2$ will be insufflated. Under endoscopic visualization trough the transanal endoscopic platform, the rectal mucosa will be incised circumferentially with cautery starting just distal to the pursestring suture, and the incision will be carried through the full thickness of the rectal wall circumferentially. Total mesorectal excision (or tumor-specific mesorectal excision) will be performed and the dissection will be extended towards the peritoneal cavity which will be entered under laparoscopic visualization. In cases of low rectal tumors close to the anorectal ring, partial or complete intersphincteric resection (ISR) may be performed prior to placement of a pursestring suture and initiation of transanal TME. The extent of rectal and mesorectal dissection performed transanally will be ascertained in CRFs, including the occurrence of any intraoperative complications. Conversion is defined as completion of the proctectomy through an open abdominal incision (≥

10 cm used for specimen extraction) due to technical difficulties or intraoperative complications.

### *7.10.3. Specimen Removal and Anastomosis*

Further rectosigmoid dissection will be performed using combined laparoscopic and transanal visualization, retraction and dissection. Additional left colon will be dissected in order to free sufficient length of colon to reach down towards the anus for planned coloanal anastomosis. The rectosigmoid will be pulled out transanally and transected. In cases where the specimen is too bulky for transanal extraction, it will be exteriorized through an abdominal incision. Stapled or handsewn coloanal anastomosis will then be completed, in a standard fashion. Since the incidence of anastomotic complications following coloanal anastomosis does not correlate with the type of anastomosis (handsewn vs. stapled), the choice of anastomosis will be determined based on the surgeons' preference[69]. In subjects with upper rectal tumors and/or in patients who have not received neoadjuvant treatment, a diverting loop ileostomy may be omitted, based on the surgeon's preference. If the surgeon elects to perform a diverting stoma, a loop of terminal ileum will be used to create a loop ileostomy in the standard fashion. A pelvic drain may or may not be positioned in the deep pelvis and passed through one of the trocar sites, based on the surgeon's preference[70]. The wounds will be closed in the standard fashion.

If at any point during transanal rectosigmoid mobilization, difficulties are encountered with visualization, safe dissection, bleeding > 500 ml and/or bleeding that prevents adequate visualization, or in the event of an intraoperative complication precluding safe completion of transanal TME, the procedure will be completed either laparoscopic, using a hand-assisted laparoscopic approach, or converted to open.

## 7.11. Pathology Assessment of the Surgical Specimen (Appendix B)

Resected specimens will be processed and analyzed by the participating institution's pathology department according to standard TME protocol[24-26, 59-60].

- The fresh specimen is examined to assess the quality of the mesorectum, photographs that include the anterior, posterior and lateral aspects of the mesorectum should be obtained.

- Prior to opening the specimen, the mesorectal fat surrounding the tumor is inked.

- The rectum is opened up to 2 cm above to 2 cm below the tumor, where the specimen is left intact. Intraluminal tumor observations and size measurements are made.

- The specimen is fixed for at least 48 hours before serial cross-sectional slicing of the specimen.

- The unopened portion of the fixed specimen is then sliced into thin transverse sections (3–5 mm in thickness).

- All of the cross-sectional rings are laid out to further assess the quality of the mesorectum and the relationship of the tumor to the lateral margin.

- Photodocumentation is performed to further document the quality of the mesorectum.

- Every effort should be made to identify at least 12 lymph nodes in the surgical specimen[60-61].

In addition, for quality control of TME grading across participating institutions, photographs of all fresh TME specimens will be independently reviewed by the pathology monitoring committee (PMC) blinded to the source of the photodocumentation.

### 7.12. Postoperative Care

Participants will be managed according to standard post-colorectal resection protocols. Diet will be advanced as tolerated and if a pelvic drain was placed, it will be removed when output is minimal as per standard practice. Subjects with stomas will be educated by the enterostomal therapy service prior to discharge. Subjects will be discharged when stable, ambulatory, with adequate pain control on oral pain medications, tolerating a low-residue diet with resumption of bowel function or a functioning ileostomy. Blood draw for CRP will be obtained from post-op day 1-4 or less if discharged prior to postop day 4.

### 7.13. Postoperative Visits

All participants will return within 2 weeks of hospital discharge for a routine postoperative visit. Subjects will subsequently return 4-6 weeks after hospital discharge for routine follow-up evaluation and thereafter following NCCN guidelines for oncologic follow-up. In subjects not requiring adjuvant treatment, evaluation of the coloanal anastomosis by gastrografin enema will also be performed at that time, as is standard practice in patients with a diverting stoma. On this visit, 30-day clinical outcomes will be collected, and ileostomy reversal will be scheduled if the anastomosis is healed. Ileostomy closure will be deferred until after completion of adjuvant therapy in subjects undergoing postoperative treatment. Any additional visits will be provided as necessary. Questionnaires to assess bowel function (COREFO, Wexner, FIQL), sexual function (FSFI or IIEF) and urinary function (IPSS) will be administered 6-8 months postoperatively in

non-diverted subjects, and 3-4 months following ileostomy closure in diverted subjects. The same questionnaires will be administrated again 12-18 months following the study procedure, or later based on the timing of ileostomy closure.

### 7.14. Postoperative Adjuvant Treatment

Adjuvant chemotherapy will be administered based on preoperative and/or postoperative recommendations from the MDT at the participating institution. Chemotherapy doses may be reduced based on toxicity and treatment interrupted if poorly tolerated by subjects. Study participants who have been upstaged to stage II or III may also receive postoperative radiation based on standard protocols. If participants choose to receive their adjuvant CRT at another institution closer to their home, care will be coordinated by the medical and radiation oncology team at the participating institution.

### 7.15. Surveillance

Patients will be followed according to the NCCN guidelines for postoperative surveillance:

- Year 1-2: History and Physical examination, CEA level every 3-6 months; CT scans of the chest, abdomen and pelvis every 6-12 months
- Year 3-5: History and Physical examination and CEA level every 6 months, CT scans of the chest, abdomen and pelvis every 6-12 months

  Year 3: A surveillance pelvic MRI will also be performed for additional evaluation of locoregional recurrence
- Year > 5: Annual history and physical examination

Patients will also be undergoing endoscopic surveillance according to standard postoperative and NCCN guidelines:

- Year 1-2: Office proctoscopy every 3-6 months. Colonoscopy will be performed 1 year postoperatively, and if normal, 3 years later, and every 5 years thereafter
- Year 3-5: Office proctoscopy every 6 months
- Year > 5: Annual proctoscopy

## 8. DATA COLLECTION

**Preoperative data collected**

- Subject registration number
- Demographics

- Past medical history
- Performance status
- Clinical examination
- Tumor location
- Preoperative staging
- Preoperative blood tests
- Colonoscopy results
- Rectal tumor biopsy results
- Neoadjuvant treatment received
- Repeat clinical examination following completion of CRT (if applicable)
- Preoperative colorectal functional outcome (COREFO questionnaire), fecal incontinence scores (Wexner and FIQL questionnaires), sexual function score (FSFI or IIEF questionnaires) and urinary function score (IPSS questionnaire) prior to neoadjuvant treatment (if applicable), preoperatively following completion of neoadjuvant treatment (if applicable).

**Intraoperative data collected**

- Date of surgery
- Operating surgeons
- Study site
- Tumor location
- Operative procedures performed
- Transanal endoscopic platform used
- Surgical techniques and surgical energy used
- Operative time
- Specimen extraction site
- Type of colorectal anastomosis
- Type of stoma created (if applicable)
- Blood loss (ml)
- Intraoperative complications
- Results of intraoperative bowel perfusion assessment (if applicable)

- Conversion (if applicable)

**Postoperative data collected**

- TME synoptic pathology report (See Appendix B)
- Photographs of the gross TME specimen
- Date of admission(s) and discharge(s)
- Duration of hospital stay(s)
- Immediate postoperative CRP value (POD 1-4 or less if shorter hospital stay)
- Postoperative complications
- Follow-up clinical evaluation reports
- Adjuvant treatment received (if applicable)
- Follow-up surveillance imaging and blood test results
- Date of ileostomy closure (if applicable)
- Adjuvant treatment received (if applicable)
- Postoperative colorectal functional outcome questionnaire (COREFO), fecal incontinence scores (Wexner and FIQL questionnaires), sexual function scores (FSFI or IIEF questionnaires) and urinary function score (IPSS questionnaire) obtained 6-8 months postoperatively if non-diverted, 3-4 months post-ileostomy closure, and 12-18 months following the study procedure (or later as detailed above).
-

## 9. DATA MANAGEMENT AND CONFIDENTIALITY

The coordinating center (Mount Sinai Hospital) has developed a set of electronic case report forms (eCRFs) using REDCap (Research Electronic Data Capture), for use with the protocol. REDCap is a secure web application designed to support data capture for research studies, providing user-friendly web-based case report forms, real-time data entry validation, audit trails and a de-identified data export mechanism to common statistical packages (SPSS, SAS, etc.). The system was developed by a multi-institutional consortium which includes the Icahn School of Medicine at Mount Sinai (ISMMS). The database is hosted at the ISMMS Datacenter. The system is protected behind a login and Secure Sockets Layer (SSL) encryption. Data collection is customized for each clinical trial based on study-specific data dictionary defined by the research team. The overall PI, coordinating center's study coordinator and their designees will have access to the entire REDCap database.

## 9.1. Data Form Review

When data forms and REDCap entries are received by the coordinating center's study coordinator, they will be reviewed for completeness, protocol treatment compliance, adverse events and response. Data submissions are monitored for timeliness and completeness of submission. Participating Institutions are notified of their data submission delinquencies in accordance with the following:

### *9.1.1. Incomplete or Questionable Data*

If study forms or REDCap eCRFs are received with missing or questionable data, the submitting institution will receive an electronic query from the study monitor. Responses to all queries should be completed and submitted within 14 calendar days. Responses may be returned electronically or within REDCap. Source documents may be required by the coordinating center to aid in source documentation verification.

### *9.1.2. Missing Forms*

If study forms or REDCap eCRFs are not submitted on schedule, the participating Institution will receive a Missing Form Report from the coordinating center noting the missing forms.  These reports are compiled by the coordinating center and distributed a minimum of four times a year.

## 9.2. Multicenter Protocol Confidentiality

All documents, investigative reports, or information relating to the participant are strictly confidential. All eCRFs including functional questionnaires completed by patients are submitted to the coordinating center through REDCap which is a secure web application designed to support data capture for research studies, and the system is protected behind a login and Secure Sockets Layer (SSL) encryption. REDCap also allows provides a de-identified data export mechanism to common statistical packages.

The regulatory binder for the study will be stored at each study site in the site's locked PI's office. An electronic copy of the regulatory binder will also be maintained on the site PI's work computer located the site PI's locked office. For the purpose of remote monitoring by the coordinating center, study sites will also upload regulatory binder documents onto the encrypted password-protected Mount Sinai Box, a secure content management and collaboration platform.Whenever reasonably feasible, any participant specific reports that are not submitted to the coordinating center through the Mount Sinai Box (i.e. source documents including pathology reports, MRI reports, operative reports, photo documentation of TME specimens, etc.) must have the participant's full name and social security number "blacked out" and the assigned subject identification number and protocol number written in (with the exception of the signed informed consent document).  Participant initials may only be included or retained for cross verification of identification. In order to protect privacy, at each investigative site, protected health information (PHI) and the main data collection databases will be maintained on a password-protected and virus scan enabled computer. The research staff at every site will be trained on the importance of confidentiality of data.

### 9.3. Record Retention

All study-related documents must be retained for the maximum period required by applicable federal regulations and guidelines or institutional policies.

### 9.4. Data Sharing

Data related to study accrual across study sites, SAEs reports, protocol deviations, IRB, DSMC, and audit reports, and other study-related data may be shared by the overall PI with the study sponsor upon request. Submitted data will be systematically de-identified prior to being shared with the sponsor.

## 10. BENEFITS AND RISKS TO HUMAN SUBJECTS

### 10.1. Potential Benefits to Subjects

Study participants may benefit from potentially reduced incisional pain and long-term risk of abdominal wall hernia formation. By enabling transanal extraction of the surgical specimen, the study procedure may reduce the number and size of abdominal wall incisions otherwise required during standard open, laparoscopic or robotic rectal cancer surgery.

### 10.2. Risks for taTME with Laparoscopic Assistance

Based on the cumulative results from over 500 cases from cases series on taTME, the risks associated with taTME with laparoscopic assistance for rectal cancer are expected to be the similar to those associated with standard open, laparoscopic, and robotic rectal cancer resection.

- Inadequate Mesorectal Excision (based on pathologic assessment of TME specimen)

  Occasional (5-20% probability)

  - Incomplete mesorectum
  - Positive resection margins
  - Less than 12 lymph nodes in non-radiated specimen[60]

The risk of incomplete mesorectal excision increases with advanced T stage and with rectal tumors located ≤ 5 cm from the anal verge (from 5-10% to > 20%[13-17]). Incomplete mesorectal resection may require additional surgery and/or adjuvant therapy to lower the risk of local, regional and distant recurrence.

- Intraoperative surgical risks

  Rare (<5% probability)

  - Conversion to open surgery

- Bleeding requiring > 4 units of blood transfusion and/or reoperation
- Bowel perforation
  - Organ injury (urethra, ureter, bladder, vagina, prostate, ovaries, uterus, spleen, kidneys)

- Early Postoperative Risks (0-30 days after surgery, typically reversible with medical and/or surgical intervention)

Common (10-40% probability)

- Dehydration
- Ileus
- Bowel obstruction
- Urinary retention
- SSI (surgical site infection)
- Abdominal or pelvic abscess
- Readmission

Occasional (5-10% probability)

- Urinary tract infection
- Renal failure
- Pneumonia
- Cardiopulmonary failure
- Deep vein thrombosis
- Anastomotic leak
- Sepsis

Rare (<5% probability)

- Pulmonary embolism
- Bleeding requiring > 4 units of blood transfusion and/or reoperation
- Reoperation
- Colovaginal fistula
- Death

- Long-term Risks (≥ 30 days after surgery, typically reversible with medical and/or intervention)

Common (10-40% probability)

– Sexual dysfunction (that persists beyond 6 months postoperatively or beyond 3 months following ileostomy closure)

– Fecal incontinence (that persists beyond 6 months postoperatively or beyond 3 months following ileostomy closure)

– Poor bowel function (that persists beyond 6 months postoperatively or beyond 3 months following ileostomy closure)

Occasional (5-10% probability)

– Incisional hernia

– Anastomotic stricture (that requires intervention)

– Local or distant cancer recurrence (defined as any recurrence diagnosed or suspected in the pelvis i.e. tumor bed, pelvic nodes, anastomosis, drain site, or perineum, occurring alone or with other sites of recurrence)

– Distant metastases (cancer recurrence occurring outside the pelvis)

***Other possible risks to consider are:***

– Risk of loss of private information

– The risks of a blood draw include pain, bruising, and the slight possibility of infection

– Risks associated with radiological scans and X- Rays

– Risks associated with MRI scans

– Reproductive risks

# 11. ADMINISTRATIVE RESPONSIBILITIES

## 11.1. Coordinating Center

### *11.1.1. Public Disclosure of Clinical Trial*

Once approved and open to enrollment, the study will be posted on the clinicaltrial.gov website, and a link to the clinicaltrials.gov posting will be posted on the study sponsors' official website (www.sages.org, www.fascrs.org ) with a description of the trial, and a list of all actively enrolling participating study sites.

#### *11.1.2. Overall Principal Investigator's Responsibilities*

The overall PI will accept responsibility for all aspects of conducting a multicenter protocol which includes but is not limited to:

- Oversee the coordination, development, submission, and approval of the protocol as well as subsequent amendments.
- Ensure that the investigators, study team members, and participating institutions are qualified and appropriately resourced to conduct the protocol.
- Submit the Multicenter Data and Safety Monitoring Plan.
- Assure all participating institutions are using the correct version of the protocol.
- Ensure that each participating investigator and study team receives adequate protocol training and/or a Site Initiation Visit prior to enrolling participants and throughout trial's conduct as needed.
- Monitor progress and overall conduct of the study at all participating institutions.
- Ensure all GCP reporting requirements are met.
- Review data and maintain timely submission of data for study analysis.
- Ensure compliance with all requirements as set forth in the Code of Federal Regulations, GCP guidelines, HIPAA requirements, and the approved protocol.
- Commit to the provision that the protocol will not be rewritten or modified by anyone other than the overall PI.
- Identify participating institutions and obtain accrual commitments prior to extending the protocol to that site.

#### *11.1.3. Coordinating Center's Responsibilities*

The coordinating center will assume the following general responsibilities:

- Assist in protocol development.
- Develop protocol-specific case report forms (CRFs) on REDCap.
- Maintain copies of Federal Wide Assurance and Institutional Review Board (IRB) approvals from all participating institutions.
- Maintain updated roster of all study participants.
- Verify eligibility.
- Manage site qualification and site initiation visits.
- Provide central participant registration, which includes review of consent and eligibility.

- Oversee the data collection process from participating institutions.
- Maintain documentation of Serious Adverse Event (SAE) reports submitted through REDCap by participating institutions and submit to the overall PI for timely review.
- Distribute adverse events reported to the overall PI that fall under the Mount Sinai Hospital IRB Adverse Event Reporting Policy to all participating investigators.
- Provide participating institutions with information regarding Mount Sinai Hospital requirements that they will be expected to comply with.
- Monitor participating institutions either by on-site or virtual monitoring.
- Maintain Regulatory documents of all participating institutions.
- Conduct regular communications with all participating institutions (conference calls, emails, etc.).
- Maintain documentation of all communications.
- Ensure that each participating institution has the appropriate assurance on file with the Office of Human Research Protection (OHRP).

## 11.2. Participating Institutions

### *11.2.1 **Participating Institution Eligibility***

#### 11.2.1.1. Rectal Cancer Volume

The coordinating center and selected participating institutions consist of US institutions where a relatively high volume of rectal cancer resections are performed by surgeons with expertise in minimally invasive pelvic surgery. All participating institutions perform 20 or more mesorectal cancer resections with TME per year, of which a minimum of 20 per year are performed using minimally invasive techniques (laparoscopy, robotic and taTME), and a minimum of 20 per year are performed with sphincter preservation (LAR). Based on participating institutions' yearly rectal cancer volume, it is anticipated that all sites will meet the minimal accrual target of 10 subjects over 2 years.

#### 11.2.1.2. Multidisciplinary Rectal Cancer Care

As part of the study site selection criteria, participating institutions are expected to have adopted systematic multidisciplinary tumor board review of every rectal cancer patients whereby patients' medical records, imaging studies, endoscopy and pathology reports are reviewed by a team of specialists in Gastrointestinal surgeons, medical oncologists, radiation oncologists, pathologists, and radiologists, and consensus regarding the best treatment option is generated. Study sites with CoC accreditation, although not mandatory, is preferred.

All sites will also be expewerected to have adopted standard TME protocols for pathologic assessment of the rectal cancer specimens

**11.2.1.3. Participating Surgeon's Experience**

As part of the study site eligibility criteria, all participating teams of surgeons must be proficient in laparoscopic and/or robotic TME and demonstrate that they have performed cumulatively an aggregate of at least 20 laparoscopically (with or without hand-assistance), and/or robotically-assisted sphincter-preserving TME for rectal cancer within the preceding 12 months. De-identified operative and pathology reports for 10 of those cases will be submitted by each participating institution and reviewed by the overall PI to determine participating institution eligibility. Proof of participation in the ACOSOG Z6051 trial will substitute for this requirement.

In addition, all participating transanal surgeons must be proficient in transanal endoscopic surgery (TES) and have performed at least 20 TES procedures within the preceding 24 months.

**11.2.1.4. Participating Surgeon's Prerequisite taTME Experience**

Another site eligibility criterion will be for participating institutions to have had prior clinical experience with taTME (having performed at least 5 cases for cancer over the preceding 12 months, either LAR or APR). The participating institution are requested to submit unedited recordings of 2 cases of LAR with taTME over the preceding 2 years, for review by the overall PI (both abdominal and transanal recordings) including at least one male patient. The operative report, pathology report, and photographs of gross TME specimens for both taTME cases will also be reviewed to confirm proficiency in study procedures, and adequacy of TME specimens.

***11.2.2. Study Staff***

The study team consists of the Site PI(s), additional surgeons participating in the study procedure, GI pathologist, their research assistant, and other collaborators at the discretion of the site's PI.

All study staff members should be HIPAA certified and have undergone protocol training. The site PI is a Board-certified colorectal surgeon with expertise in transanal endoscopic surgery and laparoscopic and/or robotic rectal cancer surgery. The research assistant should be actively involved with outpatient care and is trained in electronic clinical data collection and entry using REDCap.

***11.2.3. Participating Institution's Responsibilities***

Each participating institution is expected to comply with all applicable Federal Regulations and Mount Sinai Hospital requirements, the protocol and HIPAA requirements. All participating institutions will provide a list of personnel assigned to the role for oversight of data management at their site to the coordinating center.

The general responsibilities for each participating institution are as follows:

- Commit to the accrual of participants to the protocol.
- Submit protocol and/or amendments to their local IRB.
- Maintain an updated regulatory binder in accordance with standard GCP requirements.
- Provide the coordinating center with regulatory documents as requested.
- Participate in protocol training prior to enrolling participants and throughout the trial as needed (i.e. teleconferences).
- Update coordinating center with research staff changes on a timely basis.
- Register participants through the coordinating center.
- Submit source documents, research records, and CRFs per protocol specific submission guidelines to the coordinating center.
- Submit all Serious Adverse Events (SAEs) reports to the coordinating center and submit all SAEs to local IRB per local requirements
- Submit protocol deviations and violations to the overall PI and to local IRB per local requirements
- Have office space, office equipment, and internet access that meet HIPAA standards.

## 12. DATA MONITORING

### 12.1 Part I: Elements of Data and Safety Monitoring Plan

***Principal Monitor:*** Patricia Sylla, MD
*Last Name:* Sylla
*First Name:* Patricia
*Academic Title:* Associate Professor of Surgery, Icahn School of Medicine
*Department:* Surgery
*Mailing Address:* 5 East 98th Street Box 1259, New York, NY 10029
***Additional Monitor:*** Peter Kozuch, MD
*Last Name:* Kozuch
*First Name:* Peter
*Academic Title:* Associate Professor of Medicine Icahn School of Medicine
*Department:* Medicine
*Mailing Address:* 10 Union Sq E, suite 4C, New York, NY 10003

The study overall PI will serve as principal monitor, and will provide ongoing monitoring of protocol compliance and patients' safety, at Mount Sinai and across all investigative sites. The principal monitor for this study has extensive experience with the study procedure, anticipated AEs related to the procedure and conduct of clinical

trials. The overall PI will also be responsible for monitoring ICF completion, study endpoints, AEs and SAEs, CRFs completion and source documents data. In addition, the overall PI will review the accumulated safety and data information on a monthly basis.

#### *12.1.1. External Study Monitor: BRANY (Biomedical Research Alliance of New York)*

BRANY provides independent monitoring services for Multicenter Tisch Cancer Center investigator initiated clinical trials.

- BRANY helped develop a multicenter monitoring plan in collaboration with the overall PI.
- BRANY will provide ongoing multicenter monitoring, including remote site initiation training, remote closeout visits, and remote monitoring/auditing.
- On-site monitoring will also be performed at every study site by BRANY monitors after the first 1-5 subjects have been enrolled at each site.
- BRANY monitors will perform remote monitoring of study sites that have enrolled at least one patient into the study protocol.
- Remote site monitoring will include review of essential regulatory documents, verification that the participating institution is following the most current IRB approved protocol, confirmation that all study subjects have documented informed consents on file and in the medical record, that subjects enrolled met eligibility criteria, that source documents are accurate and complete, and that unanticipated problems involving risks to subjects are appropriately reported.
- BRANY monitors will ensure that the study sites are conducting and documenting the study protocol in accordance with regulatory requirements. BRANY will provide detailed feedback to the overall PI, study site investigators, and DSMC on any deviations, data discrepancy and/or necessary corrective action plans.

#### *12.1.2. Ongoing Monitoring of Protocol Compliance*

##### 12.1.2.1. Site Initiation Visit and Close-out Visit

The coordinating center and the BRANY monitor will conduct protocol training and remote site initiation visit (SIV). These visits will be conducted via web conference by the overall PI, coordinating center’s study staff and the BRANY monitor and the participating institutions’ PI and study staff prior following site-specific IRB approval, and prior enrollment of the first patient. Review of the protocol, screening and enrollment procedures, inclusion and exclusion criteria, study visits, calendar and procedures, AEs and SAEs reporting policies, electronic case report forms to be submitted, REDCap submission training, and monitoring plan and regulatory responsibilities of each site will be conducted. Following site initiation, the coordinating center along with the BRANY monitor will be responsible for ongoing monitoring, additional training and close-out visits at participating institutions.

**12.1.2.2. Registration Verification**

All eligibility and consent requirements are verified by the coordinating center for each participant after site registration. Participating Institutions are required to forward de-identified copies of participant's medical records and source documents to the coordinating center to aid in source documentation verification (SDV). Source documents that will be required include the signed informed consent form and the signed surgical consent for the study procedure. Additional source documents that may be requested include but are not limited to pelvic MRI report, CT or PET CT scan reports, colonoscopy and pathology reports, clinical evaluation performed by the study surgeon including tumor assessment, reports of chemoradiation regimen received (if applicable), and preoperative lab results.

**12.1.2.3. Ongoing Monitoring Activities**

Involvement in this study as a participating investigator implies acceptance of ongoing monitoring activities, potential audits or inspections, including source data verification, by representatives designated by Mount Sinai Hospital's overall PI. The purpose of ongoing monitoring activities is to examine study-related activities and documents to determine whether these activities were conducted and data were recorded, analyzed, and accurately reported in accordance with the protocol, institutional policy, Good Clinical Practice (GCP), and any applicable regulatory requirements.

All data will be monitored for timeliness of submission, completeness, and adherence to protocol requirements. Monitoring will begin at the time of participant registration and will continue during protocol performance and through study completion. Additional monitoring practices include but are not limited to source verification and review and analysis of adverse events and all associated documentation, regulatory files, and protocol deviations.

With each patient registered to the protocol, the coordinating center's study coordinator will monitor compliance with the protocol, verify eligibility and informed consent forms as well as verify timely data entry through REDCap, verify compliance with SAE reporting procedures, review and retrieve regulatory documentation, and perform quality control by comparing data from the eCRFs to the source documents of the center as per monitoring guidelines requirements.

Ongoing site monitoring will be conducted remotely for all participating institutions. Monthly meetings will be held by the overall PI and coordinating center's study coordinator to review overall accrual, timeliness of submission and completeness of data entry on REDCap, adherence to protocol requirements, and overall regulatory compliance across participating institutions.

Participating institutions will be required to participate in coordinating center-initiated teleconferences every month. Teleconferences will be used to

communicate any new information and to review study accrual, subject recruitment and enrollment, adverse events, quality assessment of TME specimens based on pathology assessment and a second independent review of photodocumentation of TME specimens by an expert pathologist, deviations and violations and any other regulatory or administrative issues.

As part of remote monitoring of participating institutions, the overall PI will review all monitoring reports to ensure protocol compliance and ability to fulfill responsibilities of participating in the study. The coordinating center may increase the monitoring activities at participating institutions that are unable to comply with the protocol, coordinating center requirements or federal and local regulations.

In addition, the coordinating center's study coordinator will conduct frequent email communications and/or telephone calls (at least monthly for each actively recruiting participating institution) with participating institutions to review issues related to data collection and management, event reporting and follow-up, staff updates and training needs and any other operational and protocol compliance-related issues.

A monthly email status update highlighting overall protocol progress and important announcements will be distributed to all the participating institutions.

### *12.1.3. Ongoing Monitoring: Quality Control*

#### 12.1.3.1 Prerequisite Competency in Study Procedures

As described in the section on participating institution eligibility criteria, eligible site surgeons must have performed a minimum of 5 taTME cases over the preceding 12 months and submitted pathology and operative reports, in addition to video recordings and photographs of TME specimens from 2 taTME cases performed over the preceding 2 years for review by the overall PI to confirm proficiency in taTME technique and good quality of TME specimen

#### 12.1.3.2. Monitoring of Study Procedures

Study surgeons will be required to record videos of all transanal endoscopic procedures. The site surgeon may be requested to submit unedited transanal procedural videos for ongoing monitoring of the quality of the taTME procedures by the overall PI. Videos selected for auditing will be reviewed by an independent reviewer who is an expert in taTME procedures blinded to the study site and surgeon. Independent video assessment of the adequacy of taTME dissection will be forwarded to the coordinating center and reviewed by the overall PI. Any major deviation from recommended TME dissection principles will be addressed with the necessary corrective action plans.

**12.1.3.3. Monitoring of Quality Assessment of TME Specimens**

All gross TME specimens are photographed by the study sites pathologist as per standard protocol, to document the integrity of the mesorectum. TME specimen photographs will also be forwarded to the coordinating center for review. A member of the PMC will participate in the remote site initiation training to review standards for TME assessment to all the study sites. Independent review of photo documentation of all TME specimens will be performed by the pathology monitoring committee (PMC) blinded to the study site and surgeon. Any major deviation from standard TME protocols for pathologic assessment of rectal specimens as reported by the PMC will be addressed by the coordinating center with the necessary corrective action plans.

**12.1.3.4. Interim Analysis and Study Stopping Rule**

After 50% of the 100 study subjects have been enrolled and undergone the study procedure, interim analysis of futility of the mesorectal excision achieved by the study procedure will be performed by the coordinating center and reviewed by the DSMC. In the event that the overall rate of incomplete TME exceeds 26% of subjects (incomplete mesorectum in ≥ 26% specimens) and/or positive resection margins are reported in ≥ 26% specimens, the trial will be placed on hold and the interim results reviewed with the coordinating center as the probability of rejecting the null hypothesis will be 0.005 or less based on a binomial test. The IRB and study sponsor will be notified immediately of the suspension of the study. The study will remain on hold until it is determined by the Mount Sinai DSMC and IRB that it can safely resume. We do not expect higher rates of unusual complications than standard LAR.

The stopping rule for this trial is based on the reported incidence of incomplete TME (incomplete mesorectum or R0 resection) following standard TME ranging from 10 to >20% in large studies[3,13-17]. Incidence of incomplete TME up to 26% halfway through the trial, when approximately 50 subjects have undergone the study procedure, would be acceptable based on the cumulative experience from published series in over 500 patients[35-51].

**12.1.3.5. Early Stopping Rule**

At each accruing study site, report of 2 cases with incomplete mesorectum and/or 2 cases with positive resection margins following taTME will prompt video auditing of the most recent taTME case resulting in incomplete TME, with assessment of the adequacy of taTME dissection by an independent reviewer who is an expert in taTME, blinded to the study site and surgeon. Any major deviation from recommended TME dissection principles will be addressed with the necessary corrective action plans. Report of a third case with incomplete mesorectum and/or positive resection margins, despite prior feedback and corrective action plan, will result in early stopping of the study at the study site. The study will be placed on hold at the study site and the results reviewed with the coordinating center. The local IRB, coordinating center IRB, study DSMC and study sponsor will be notified immediately of the suspension of the study at the

study site. The study will remain on hold until it is determined by the local IRB, the Mount Sinai DSMC and IRB, that it can safely resume.

***12.1.4. Audit of Registration Process***

The BRANY study monitor will remotely review all registration activity that occurred over the course of the previous 3 months. All (100%) of registrations audited to confirm that all registration documentation was completed accurately by study staff and that all eligibility criterial were met. Any errors that are found will be reported to the relevant study team by the coordinating center's study coordinator and the overall PI. Summary data listing the total number of central registration reviewed and the percentage of those that were found to have one or more errors will be logged.

***12.1.5. Study Site Audits***

One full on-site audit at each participating institution will be conducted remotely by BRANY after the first 1-5 participants have been treated on protocol at the site. In addition, 100% of participants will be subsequently remotely monitored at each site for the first year. Thereafter, 25% of participants will be randomly audited remotely at each site for the remainder of the study. Each study site will maintain an electronic copy of their regulatory binder onto the encrypted password-protected Mount Sinai Hospital onto the encrypted password-protected Mount Sinai Box (https://mountsinai.account.box.com/login). Regulatory documents will be remotely reviewed by the coordinating center and study monitor, which may include the IRB approved protocol, IRB approvals, amendments and continuing reviews, de-identified copies of informed consents, protocol deviation, exception and violation log, SAE notifications and submissions to the IRB and sponsor, significant communications with the overall PI and Study Monitor, initial protocol specific training records, delegation of authority log, screening and enrollment logs, and other regulatory documents that do not contain any PHI. Eligibility checklist, record of registration, case history, eCRFs and completed questionnaires will also be reviewing, along with all relevant source documentation.

If major violations which impact participant safety or the integrity of the study are found, on-site audits may be conducted for one or more study participants if deemed appropriate by the DSMC and/or overall PI.

**12.1.5.1. Review of Audits**

The coordinating center will review all final remote audit reports and corrective action plans if applicable. The coordinating center must forward these reports to the DSMC. Based upon the audit assessments, the DSMC could accept or conditionally accept the audit rating and final report. Conditional approval could require the coordinating center to implement recommendations or require further follow-up. For unacceptable audits, the DSMC would forward the final audit report and corrective action plan to the Mount Sinai Hospital IRB as applicable.

#### 12.1.5.2. Corrective Actions

Participating institutions that fail to meet the performance goals of accrual, submission of timely accurate data, adherence to protocol requirements, and compliance with state and federal regulations, will be recommended for a three-month probation period. Such institutions must respond with a corrective action plan and must demonstrate during the probation period that deficiencies have been corrected, as evidenced by the improved performance measures. participating institutions that fail to demonstrate significant improvement will be considered by the coordinating center and study sponsor for revocation of participation.

The DSMC will review the protocol quarterly from the time of first subject enrollment and/or more often if required to review adverse events and accrual data. Information to be provided to the committee may include: up-to-date participant accrual; all grade 2 or higher unexpected adverse events that have been reported; summary of all deaths occurring within 30 days of intervention; up-to-date summary of TME completion rate, audit results, and a summary provided by the study team. Other information (pathology reports, operative reports, etc.) will be provided upon request.

Should a temporary or permanent suspension of the study occur, the overall PI will notify the participating institutions, IRB, and study sponsor.

### *12.1.6. Procedures for AE and SAE Recording and Reporting*

All adverse events, both serious and non-serious, and deaths that are encountered during the study procedure, within 30 days of the study procedure and thereafter throughout the rest of the study should be followed to their resolution, or until the study investigator assesses them as stable, or the study investigator determines the event to be irreversible, or the participant is lost to follow-up. The presence and resolution of AEs and SAEs (with dates) should be documented on the appropriate case report form and recorded in the participant’s medical record to facilitate source data verification.

Participating investigators will assess the occurrence of AEs and SAEs at all participant evaluation time points from the day of surgery throughout the rest of the study. . All AEs and SAEs whether reported by the participant, discovered during questioning, directly observed, or detected by physical examination, imaging, pathology or laboratory testing or other means, will be recorded in the participant’s medical record and on the appropriate study-specific eCRFs.

#### 12.1.6.1. Adverse Event Characteristics

All adverse events and postoperative complications during the study period will be reported and graded according to the Clavien-Dindo system[72]. Grades will be recorded on the eCRFs.

| Grade | Definition of Surgical Complication Grade[72] | Clinical Example of Complication Grade |
|---|---|---|
| Grade I | Any deviation from the normal post-operative course without the need for pharmacological treatment or surgical, endoscopic, and radiological interventions | Non-infectious diarrhea |
| Grade II | Requiring pharmacological treatment with drugs other than such allowed for grade I complications. Blood transfusions and total parenteral nutrition are also included. | Infectious diarrhea requiring antibiotics |
| Grade IIIa | Requiring surgical, endoscopic or radiological intervention not under general anesthesia | Radiologic or endoscopic intervention of an anastomotic leak that is taken care of outside of the operating room |
| Grade IIIb | Requiring surgical, endoscopic or radiological intervention under general anesthesia | Surgical intervention of an anastomotic leak that is taken care of in the operating room |
| Grade IVa | Life-threatening complication (including CNS complications)[a] requiring IC/ICU management with single organ dysfunction (including dialysis) | Necrotizing pancreatitis |
| Grade IVb | Life-threatening complication (including CNS complications)[a] requiring IC/ICU management with multi organ dysfunction | Necrotizing pancreatitis with hemodynamic instability |
| Grade V | Death of a patient | Organ failure after surgical intervention |
| Suffix “d” | If the patient suffers from a complication at the time of discharge, the suffix “d” (for disability) is added to the respective grade of complication. This label indicates the need for a follow-up to fully evaluate the complication. | |

[a] Brain hemorrhage, ischemic stroke, subarachnoidal bleeding, but excluding transient ischemic attacks

**12.1.6.2. Serious Adverse Event Reporting**

All SAEs that occur during the study procedure, within 30 days of the study procedure and thereafter throughout the rest of the study must be reported directly by the site PI or designee to the IRB of record according to the local IRB policies and procedures in reporting SAEs. All SAEs must also be reported to the overall PI within 24 hours of learning of the occurrence. The overall PI or designee will submit SAEs from participating institutions to the Mount Sinai Hospital IRB if they meet the Mount Sinai Hospital SAE reporting requirements.

SAEs include the following:

- Grade 2 (moderate) and Grade 3 (severe) events that are unexpected and at least possibly related/associated with the study procedure

- All Grade 4 (life-threatening or disabling) events that are unexpected or not specifically listed in the protocol as not requiring reporting.

- All Grade 5 (fatal) events while the participant is enrolled and actively participating in the trial OR when the event occurs within 30 days of the study procedure.

When reporting SAEs to the overall PI, only a brief description is required within 24 hours followed by a detailed report to follow promptly with the subject's assigned code number and any relevant support documents. Within the following 48 hours, the participating investigator must provide follow-up information on the SAE. Follow-up information should describe whether the event has resolved or continues, if and how the event was treated. All SAEs must be submitted to the coordinating center by uploading the SAE form to the Mount Sinai Box

#### 12.1.6.3 Protocol-specific Expedited Serious Adverse Event Reporting Exclusions

For this protocol, the SAEs/grade listed below do not require expedited reporting to the overall PI or to the Mount Sinai Hospital IRB. However, they still must be reported through the routine reporting mechanism (eCRF).

| **Serious Adverse Event** | **Definition** | **Grade** | **Hospitalization/ Prolongation of Hospitalization** | **Attribution** | **Comments** |
|---|---|---|---|---|---|
| Postoperative ileus | Insertion or re-insertion of NGT after surgery, or return to NPO status for nausea and/or vomiting | 2-4 | Prolonged hospitalization or Hospitalization | Possible, probable, definite | Ileus is a very common AE following rectal cancer resection and usually resolves with conservative measures |
| Dehydration | Excessive loss of body fluids necessitating IV repletion | 2-4 | Prolonged hospitalization or Hospitalization | Possible, probable, definite | High ileostomy output is a very common AE following ileostomy creation |
| Urinary tract infection | Culture growth of $\geq 10^3$ colony forming units (cfu)/mL of uropathogenic bacteria in the presence of symptoms or signs of UTI | 2-4 | Prolonged hospitalization | Possible, probable, definite | UTI is a common AE following abdominal surgery and is related to the use of urinary catheters |
| Urinary retention | Impaired bladder emptying requiring re-insertion of a urinary catheter | 2-4 | Prolonged hospitalization or Hospitalization | Possible, probably, definite | Urinary retention is a common AE following rectal cancer surgery |

#### 12.1.6.4 Adverse Event Reporting

All AEs must be reported in routine study data submissions to the overall PI on the eCRF. AEs reported through the expedited process to the IRB must also be reported in routine study data submissions.

### 12.2. Part II. Data Monitoring Committee/Data Safety Monitoring Board (DMC/DSMB)

The Tisch Cancer Center Data and Safety Monitoring Committee (DSMC) will review and monitor adverse events and accrual data from this study. The committee is composed of clinical specialists with expertise in gastrointestinal oncology and who have no direct relationship with the study. Information that raises any questions about participant safety will be addressed with the Overall PI and study team. The coordinating center will distribute DSMC summary reports to collaborating clinical sites for submission to the site's local IRB.

The Tisch Cancer Center DSMC will review the protocol quarterly from the time of first subject enrollment to review adverse events and accrual data. Information to be provided to the committee may include: up-to-date participant accrual; all grade 2 or higher unexpected adverse events that have been reported; summary of all deaths occurring within 30 days of intervention; any response information, on-site audit reports, and a summary provided by the study team. Other information (pathology reports, operative reports, etc.) will be provided upon request.

## 13. WITHDRAWAL OF SUBJECTS

Patients may withdraw from this study at any time without penalty. Following enrollment and completion of the study procedure, participants will be withdrawn from the study when any of the following criteria apply:

- Patient's desire for any reason to withdraw consent
- Failure of the patient to adhere to protocol requirements
- Death
- Administrative reasons
- Lost to follow-up

The reason for withdrawing a participant from a study, and the date the participant was removed, must be documented in the off-study eCRF. Study-specific data collection (functional questionnaires) will cease immediately following withdrawal from the study but postoperative and oncologic data collection will continue unless the participant withdraws consent for data collection/submission.

In the event a study participant wishes to withdraw consent, the subject will be asked whether he will allow site staff to continue to review his/her medical chart and records and/or contact him/her for associated health status information for up to 5 years after the surgical procedure. Subject will be informed that data may be collected from medical records from routine care and handled the same way as research data.

In the event participants are withdrawn from the study following the study procedure, they may be replaced by additional participants as their participation contributed to the overall accrual at the given study site.

## 14. ECONOMIC IMPACT ON SUBJECTS

Participation in the study may lead to added costs to the subject or their insurance. The cost of the study procedure (transanal total mesorectal excision with laparoscopic assistance) will be charged to the insurance company. Subjects may be charged for portions of the care received during the research study that are considered standard of care. Subjects may be responsible for co-payments and deductibles that are typical for their insurance coverage.

## 15. PAYMENT TO SUBJECTS

Subjects will not receive remuneration for their participation in the study.

## 16. PROVISIONS FOR RESEARCH RELATED HARM/ INJURY

If a complication or injury occurs as a result of the study procedure, the Hospital will continue to provide medical care and all the necessary medical, surgical, and psychological resources necessary to facilitate the subject's recovery. The Hospital may bill the subject's insurance or other third parties if appropriate, for the costs of the care received for treatment of the complication, but the subject may be responsible for some of the costs. The Hospital will not pay subjects or provide any other compensation for the complication or injury.

## 17. STATISTICAL ANALYSIS OF ENDPOINTS

### 17.1. Primary Endpoints

The primary endpoint of the study is the rate of complete and near-complete mesorectal excision achieved by taTME, based on standard pathologic guidelines on pathology evaluation of TME specimens. The number of subjects planned to be enrolled in the study is 100 over a 2-year accrual period.

The proportion of subjects in whom good quality mesorectal excision is achieved will be determined, based on pathological assessment of complete and near-complete mesorectum. With a sample size of 100, the one-sided binomial test will reject the null hypothesis that the success rate is $\leq$ 80% if the experimental approach leads to efficacy of the total mesorectal excision for 87 or more subjects. This design achieves a power of 87% using one-sided binomial test for non-inferiority with 5% type 1 error assuming the true success rate is 90%.

### 17.2. Secondary Endpoints

Secondary endpoints include (1) pathology assessment of the specimen, (2) 30-day perioperative complications, (3) long-term complications, (4) functional results (bowel function, urinary function, and sexual function), and (5) long-term oncologic outcomes (recurrence and survival).

The Pathology assessment of the surgical specimen will be based on standard guidelines on assessment of TME specimens, including assessment of surgical margins,

lymph node, TNM stage and other histopathologic tumor features (synoptic report, Appendix B).

The overall morbidity and mortality rate of the study procedure will be calculated, as well as the incidence of specific types of complications. Overall and disease-free survival data will be obtained.

Postoperative changes in bowel function and fecal continence scores (Wexner, FIQL, COREFO), sexual function and urinary function, will be assessed by comparing functional scores obtained preoperatively and at 2 separate postoperative time points, using respective validated instruments. The COREFO questionnaire is a validated tool to assess bowel function after colorectal surgery[64]. The Wexner Fecal Incontinence Score[63] and Fecal Incontinence Quality of Life Scale[65] (FIQL) have been validated to evaluate and assess the severity of fecal incontinence, respectively, based on patient symptoms. The International Prostate Symptom Score is the most commonly used instrument used to assess the incidence and severity of urinary dysfunction following rectal cancer surgery in men and women[68,71]. Both the International Index of Erectile Function Questionnaire[67] (IIEF) and the Female Sexual Function Index[66] (FSFI) are well validated instruments to assess sexual function in men and women respectively.

Subjects will be asked to complete the questionnaires at the following time points:

1. Prior to start of neoadjuvant therapy, if applicable
2. Preoperatively (between completion of neoadjuvant therapy and the study procedure, if applicable)
3. 6 months postoperatively in non-diverted subjects or 3-4 months following ileostomy closure
4. 12-18 months following the study procedure

Postoperative functional scores will be compared with preoperative scores. Based on our experience using functional questionnaires in our colorectal surgery and pelvic floor disorder practice, we anticipate a 50-75% compliance rate with filling the questionnaires.

Subjects will be excluded from a specific functional analysis if they complete less than 20% of questions from a given instrument.

In the analyses of secondary endpoints, the Kaplan-Meier method will be used to estimate overall survival (OS) and recurrence free survival (RFS) distribution functions with the log-rank test used to compare distributions between subgroups of patients. Cumulative incidence functions (CIF) will be used to estimate time to locoregional recurrence and time to distant metastases in a competing risk setting. Locoregional recurrence is defined as the time from the date of surgery to the date of first locoregional recurrence. Both, distant metastasis and death due to any cause will be treated as competing risks. The locoregional recurrence time for patients without a locoregional recurrence or a competing risk prior to the data cutoff will be censored at the date of last adequate tumor assessment. Distant metastasis is defined as the time from the date of surgery to the date of first distant metastasis. Both, locoregional recurrence and death

due to any cause will be treated as competing risks. The distant metastasis time for patients without a distant metastasis or a competing risk prior to the data cutoff will be censored at the date of last adequate tumor assessment. Univariable and multivariable hazard ratios for OS and RFS will be estimated using Cox proportional hazards models while hazard ratios for locoregional recurrence and distant metastases will be estimated using Fine and Gray's extension of Cox regression which models the hazards of the cumulative incidence function. All multivariable models will be used to adjust for confounding factors such as age, gender and race. Recurrence rates and postoperative complication rates will be estimated with a Poisson regression model with an offset for person-days followed. Mixed effects models with random effects or repeated measurements will be used to account for within-subject correlations arising from longitudinal data. Nonparametric methods (e.g., Wilcoxon rank sum test) will be applied for variables with a non-normal distribution. Multiple imputation methods will be applied for missing data when appropriate.

Data from all participants who undergo transanal TME with laparoscopic assistance will be analyzed. All participants will be evaluable for safety from the time of their procedure.

## 18. SHARING RESULTS WITH SUBJECTS

Individual subject outcomes of study procedure for rectal cancer (final pathologic tumor staging) will be communicated to subjects and their caregivers by the study surgeon, as per standard practice. Results of the overall study will be shared with subjects upon request, after completion of the study, by direct communication by the subject's study surgeon or designee. Incidental findings during preoperative staging, at the time of completion of the study procedure, or upon subsequent surveillance imaging, will be communicated to the subjects and their caregivers as per standard practice.

## 19. PROVISIONS TO PROTECT THE PRIVACY OF SUBJECTS

Eligible subjects will be approached about study participation at the time of initial rectal cancer evaluation. The study will be first introduced by their primary surgeon or other caretakers. Only if the subjects are interested in hearing more about the study, will they have the study and study procedure described in details by a study staff member who is not the primary surgeon. From the first description of the study procedure, through enrollment, and the conduct of the study, steps will be taken to protect study participants' privacy interests. If interested in participating in the study, the participant will be provided with a copy of the informed consent to take home and review with their family and/or physician. The consent form will list the contact information for the site principal investigator, and research nurse, and the subjects will be encouraged to reach out to the study team if they have any study-related questions, issues or concerns. Study data will be collected during standard office visits related to rectal cancer care, during hospital admissions and during standard postoperative visits. In order to protect subject's privacy interests, subjects will be encouraged to complete functional questionnaires electronically through REDCap rather than filling out paper form questionnaires. Regarding long-term follow-up, subjects may elect to be contacted by email or mail rather than by phone.

## 20. VULNERABLE POPULATIONS

Vulnerable populations will not be included in this study.

| *Include* | *Exclude* | *Vulnerable Population Type* |
|---|---|---|
| | *X* | *Adults unable to consent* |
| | *X* | *Individuals who are not yet adults (e.g. infants, children, teenagers)* |
| | *X* | *Wards of the State (e.g. foster children)* |
| | *X* | *Pregnant women* |
| | *X* | *Prisoners* |

## 21. NON-ENGLISH SPEAKING SUBJECTS

This study requires completion of functional questionnaires prior to the study procedure and at several postoperative time points. While the Wexner score, FIQL, FSFI, IIEF and IPSS questionnaires have been validated in Spanish, they have not been systematically validated in many other languages. In addition, the COREFO questionnaire has not yet be validated in Spanish or any other languages. Because it is imperative that participants fully understand the questions related to fecal continence, sexual and urinary function in order to complete the questionnaires, only English speakers will be allowed to participate in the study.

## 22. REGULATORY REQUIREMENTS

The following section will clarify Mount Sinai Hospital Requirements and further detail the expectations for participating in a Mount Sinai Hospital Multicenter protocol.

### 22.1. Protocol Distribution

The coordinating center will distribute the final Mount Sinai Hospital IRB approved protocol, informed consent and any other necessary documents, as well as any subsequent amended protocols to all participating institutions for submission, review and approval by each local IRB

### 22.2. Protocol Revisions and Closures

The participating institutions will receive notification of protocol revisions and closures from the coordinating center. It is the individual participating institution's responsibility to notify its IRB of these revisions.

- **Non-life-threatening revisions:** participating institutions will receive written notification of protocol revisions regarding non-life-threatening events from

the coordinating center. Non-life-threatening protocol revisions must be IRB approved and implemented within 90 days from receipt of the notification.

- **Revisions for life-threatening causes:** participating institutions will receive immediate notification from the coordinating center concerning protocol revisions required to protect lives with follow-up by fax, mail, e-mail, etc. Life-threatening protocol revisions will be implemented immediately followed by IRB request for approval.

- **Protocol closures and temporary holds:** participating institutions will receive notification of protocol closures and temporary holds from the coordinating center. Closures and holds will be effective immediately. In addition, the coordinating center, will update the participating institutions on an ongoing basis about protocol accrual data so that they will be aware of imminent protocol closures.

### 22.3. Informed Consent Requirements

The Mount Sinai Hospital approved informed consent document will serve as a template for the informed consent for participating institutions. The participating institution consent form must follow the consent template as closely as possible and should adhere to specifications outlined in the Mount Sinai Hospital Guidance Document on Model Consent Language for Investigator-Initiated Multicenter Protocols. This document will be provided separately to each participating institution.

Participating institutions are to send their version of the informed consent document and HIPAA authorization, if a separate document, to the coordinating center for review and approval prior to submission to their local IRB. The approved consent form must also be submitted to the coordinating center after approval by the local IRB.

The Principal Investigator (PI) at each participating institution will identify the members of the study team who will be obtaining consent and signing the consent form for therapeutic protocols.

### 22.4. IRB Documentation

The following must be on file with the coordinating center:

- Approval letter of the participating institution's IRB
- Copy of the Informed Consent Form approved by the participating institution's IRB
- Participating IRB's approval for all amendments

It is the participating institution's responsibility to notify its IRB of protocol amendments. Participating institutions will have 90 days from receipt to provide the coordinating center their IRB approval for amendments to a protocol.

## 22.5. IRB Re-approval

Verification of IRB re-approval from the participating institutions is required in order to continue research activities. There is no grace period for continuing approvals.

The coordinating center will not allow sites to continue any research activities if a re-approval letter is not received from the participating institution on or before the anniversary of the previous approval date.

## 22.6. Mount Sinai Hospital Multicenter Protocol Registration Policy

### *22.6.1. General Guidelines for Study Registration*

The coordinating center will register eligible participants through its study coordinator. Registration must occur prior to the study procedure. Any participant not registered to the protocol before the scheduled date of the study procedure will be considered ineligible and registration will be denied.

An investigator will confirm eligibility criteria and a member of the study team will complete the protocol-specific eligibility checklist.

Following registration, participants may undergo the study procedure. Issues that would case delay in the study procedure should be discussed with the overall PI. If a participant does not undergo the study procedure within 14 days of study registration, the participant’s registration on the study must be cancelled, and the coordinating center’s study coordinator should be notified as soon as possible.

### *22.6.2. Registration Process for the Coordinating Center*

Participants must have enrolled in the study before being asked to complete pre-treatment functional questionnaires. Participants must be registered to the study with the coordinating center’s study coordinator before undergoing the study procedures. The study procedure may not be performed until the participating institution receives a faxed or e-mailed copy of the participant’s registration confirmation memo from the coordinating center. Study procedures must be initiated per protocol guidelines. The sponsor and Mount Sinai Hospital IRB must be notified of any exceptions to this policy.

### *22.6.3 Eligibility Exceptions*

The coordinating center will make no exceptions to the eligibility requirements for a protocol without Mount Sinai Hospital IRB approval. The coordinating center requires each institution to fully comply with this requirement.

### *22.6.4. Verification of Registration*

A Registration confirmation letter for participants registered to Mount Sinai Hospital Multicenter Protocol will be emailed to the registering institution within

one business day of the registration. The study procedure may not be initiated until the site receives an e-mailed copy of the Registration confirmation letter.

#### *22.6.5. Subject Registration Number*

Following successful registration of a subject by the coordinating center, the participant will be assigned a 4-digit Registration Identification Number starting with “R-“. The first 2 digits of the Subject ID Number refer to the study site, and the last 2 digits refer to the subject number (example R-02-04). The Subject Registration Number must be used on all REDCap eCRF forms, de-identified documents, and correspondence with the coordinating center and study monitor.

### 22.7. Protocol Deviations, Exceptions and Violations

Federal Regulations require an IRB to review proposed changes in a research activity to ensure that researchers do not initiate changes in approved research without IRB review and approval, except when necessary to eliminate apparent immediate hazards to the participant. Mount Sinai Hospital requires all departures from the defined procedures set forth in the IRB approved protocol to be reported to the overall PI, who in turn is responsible for reporting to the Mount Sinai Hospital IRB.

Protocol Deviation: Any departure from the defined procedures set forth in the IRB-approved protocol which is prospectively approved prior to its implementation.

Protocol Exception: Any protocol deviation that relates to the eligibility criteria, e.g. enrollment of a participant who does not meet all inclusion/exclusion criteria.

Protocol Violation: Any protocol deviation that was not prospectively approved by the IRB prior to its initiation or implementation.

#### *22.7.1 Reporting Procedures*

Mount Sinai Hospital: is responsible for ensuring that clear documentation is available in the medical record and/or regulatory documents to describe all protocol exceptions, deviations and violations. The overall PI is responsible for ensuring that all protocol violations/deviations and exceptions are promptly reported per Mount Sinai Hospital IRB guidelines.

Participating Institutions: Protocol deviations require prospective approval from the Mount Sinai Hospital IRB. The participating institution must submit the violations/deviations/exceptions reports and deviation requests to the IRB or record and to the overall PI. The overall PI will then submit the reports and deviation requests to the Mount Sinai Hospital IRB. Upon Mount Sinai Hospital IRB approval the deviation is submitted to the participating institution IRB, per institutional policy. A copy of the participating institution’s IRB report and

determination will be forwarded to the coordinating center within 10 business days after the original submission.

All protocol violations must be sent to the Overall PI at the coordinating center in a timely manner.

Coordinating Center: Upon receipt of the violation/deviation report from the participating institution, the coordinating center will submit the report to the Overall PI for review. Subsequently, the participating institution's IRB violation/deviation report will be submitted to the overall PI for review per Mount Sinai Hospital IRB reporting guidelines.

### 22.8. Safety Assessments

The study teams at all participating institutions are responsible for protecting the safety, rights and well-being of study participants. Recording and reporting of adverse events that occur during the course of a study help ensure the continuing safety of study participants.

All participants undergoing the study procedure will be evaluated for safety. The safety parameters include all laboratory and radiology tests and pathology findings, physical examination findings, and spontaneous reports of adverse events reported by participants. Life-threatening adverse events must be reported immediately to the coordinating center.

Additional safety assessments and adverse event monitoring is outlined in the protocol.

### 22.9 Guidelines for Reporting Adverse Events

Guidelines for reporting AEs and SAEs were detailed previously in this protocol (See Section 12.1.6. Procedures for AE and SAE Recording and Reporting).

The coordinating center will maintain documentation of all participating institution Serious Adverse Events reports and be responsible for communicating to all participating investigators, any observations reportable under the Mount Sinai Hospital IRB reporting requirements. Participating investigators will review any distributed SAE reports, send a copy to their IRB according to their local IRB's policies and procedures, and file a copy with their regulatory documents.

## 23. MOUNT SINAI IRB REVIEW HISTORY

| **Version & Date** | **Summary of Changes** |
|---|---|
| V1.0, approved 21st Apr 2017 | n/a- original protocol approved by coordinating center IRB |
| V2.0, approved 18th May 2017 | • Clarification of eligibility criteria<br>• Clarification of post-operative complications<br>• Albumin added to required preoperative lab values<br>• Clarification of required postoperative follow-up with adjustment based on most recent NCCN guidelines<br>• Change in study biostatistician<br>• Update in participating sites |
| V3.0, approved 19th Sept 2017 | • The protocol was revised to allow research personnel (delegated to do so on the log of staff) to obtain consent from study patients.<br>• Clarification was added that study patients can undergo the study procedure at both Mount Sinai Main campus and Mount Sinai Beth Israel (Downtown) (coordinating center specific. N/A for external sites)<br>• Pathology report template was modified.<br>• Some orthographic errors were corrected<br>• One external site has been added to the project (Cedar Sinai Los Angeles) (coordinating center specific. N/A for external sites)<br>• Study advertisement on Clinical trial page of MountSinai.org website, social media networks (twitter and facebook) and use of ResearchMatch.org as a recruitment tool for this research study/protocol (“recruitment access”) to send the attached study recruitment message to potential study volunteers through social media and ResearchMatch.org (coordinating center specific. N/A for external sites)<br>• Sinai Personnel changes: Two Investigators have been added to the study: Dr. Popowich and Dr. Fernandez-Ranvier. One investigator has been removed: Dr. Sanghyun Kim (coordinating center specific. N/A for external sites) |
| V4.0, approved 28th August 2018 | • Added PET CT of chest abdomen and pelvis as an alternative staging imaging to standard CT scans, as per NAPRC standards (National Accreditation Program for Rectal Cancer 2017 edition).<br>• Added clarification that subjects being enrolled in the study post neoadjuvant treatment completion must have had staging imaging no more than 3 months prior to the start of neoadjuvant<br>• Added blood draws for CRP in immediate postoperative period (POD1-4).<br>• Change in study biostatistician<br>• Deleted the name of a colorectal surgeon<br>• Changed PI for Florida Hospital<br>• Clarified frequency of monitoring calls between coordinating center and external sites<br>• Removed Intratumoral Lymphocytic Response and Peritumoral Lymphocytic Response (Crohn's like) questions from the TME synoptic report<br>• Sinai Personnel changes |
| V5.0, approved 30th Aug 2019 | • Update in participating sites<br>• Change in study biostatistician<br>• Change in coordinating center personnel |

| | |
|---|---|
| Addendum 1, approved 11th May 2020 | • Protocol addendum to allow for remote follow-up and surveys/assessments during COVID-19 epidemic<br>• COVID-19-related protocol deviations will be reported monthly to the IRB |
| V6, approved 19th Sept 2020 | • Added MRI pelvis at Y3 follow up<br>• Clarified timing of postoperative functional questionnaire<br>• Created protocol addendum to allow for telehealth visits and extended windows for oncologic follow ups during COVID 19 pandemic disruption using a single PD tracker (site specific excel sheet) submission at the end of the COVID 19 pandemic disruption.<br>• Clarified number of patients to be consented/enrolled vs registered/undergo study procedure<br>• Lengthened enrollment period to 3 years<br>• Added possibility of competitive enrollment at sites<br>• Sinai personnel and location changes (Coordinating Center specific. N/A for external sites) |

***REFERENCES***


1. Kim SH, Bae KB, Kim JM, Shin JH, et al. Oncologic Outcomes and Risk Factors for Recurrence after Tumor-specific Mesorectal Excision of Rectal Cancer: 782 Cases. J Korean Soc Coloproctol. 2012; 28:100-7

2. Law WL, Chu KW. Anterior resection for rectal cancer with mesorectal excision: a prospective evaluation of 622 patients. Ann Surg. 2004; 240:260-8.

3. Van der Pas MH, Haglind E, Cuesta MA, Fürst A, et al; COlorectal cancer Laparoscopic or Open Resection II (COLOR II) Study Group. Laparoscopic versus open surgery for rectal cancer (COLOR II): short-term outcomes of a randomised, phase 3 trial. Lancet Oncol. 2013; 14:210-8.

4. Kapiteijn E, Marjnen C, Nagtegaal I, Putter H et al. Preoperative radiotherapy combined with total mesorectal excision for resectable rectal cancer. New Engl J Med 2001; 345:638-46.

5. Swedish Rectal Cancer Trial. Improved survival with preoperative radiotherapy in resectable rectal cancer. New Engl J Med 1997; 336:980-7.

6. Sauer R, Becker H, Hohenberger W, Rodel C, et al; German Rectal cancer Study Group. Preoperative versus postoperative chemoradiotherapy for rectal cancer. N Engl J Med 2004; 351:1731-40.

7. García-Aguilar J, Hernandez de Anda E, Sirivongs P, Lee SH, et al. A pathologic complete response to preoperative chemoradiation is associated with lower local recurrence and improved survival in rectal cancer patients treated by mesorectal excision. Dis Colon Rectum 2003; 46:298-304.

8. Sebag-Montefiore D, Stephens RJ, Steele R, Monson J, Grieve R, et al. Preoperative radiotherapy versus selective postoperative chemoradiotherapy in patients with rectal cancer (MRC CR07 and NCIC-CTG C016): a multicentre, randomised trial. Lancet 2009; 373:811-20.

9. Siegel R, Burock S, Wernecke KD, Kretzschmar A, et al. Preoperative short-course radiotherapy versus combined radiochemotherapy in locally advanced rectal cancer: a multi-centre prospectively randomised study of the Berlin Cancer Society. BMC Cancer 2009; 9:50.1471-2407-9-50.

10. Påhlman L, Bohe M, Cedermark B, Dahlberg M, et al. The Swedish rectal cancer registry. Br J Surg 2007; 94:1285-92.

11. Matthiessen P, Hallböök O, Rutegård J, Simert G, et al. Defunctioning stoma reduces symptomatic anastomotic leakage after low anterior resection of the rectum for cancer: a randomized multicenter trial. Ann Surg 2007; 246:207-14.

12. den Dulk M, Smit M, Peeters KC, Kranenbarg EM, et al; Dutch Colorectal Cancer Group. A multivariate analysis of limiting factors for stoma reversal in patients with rectal cancer entered into the total mesorectal excision (TME) trial: a retrospective study. Lancet Oncol 2007; 8:297-303.

13. Quirke P, Steele R, Monson J, Grieve R, et al. Effect of the plane of surgery achieved on local recurrence in patients with operable rectal cancer: a prospective study using data from the MRC CR07 and NCIC-CTG CO16 randomised clinical trial. Lancet 2009; 373:821-8.

14. Garlipp B, Ptok H, Schmidt U, Stübs P, et al. Factors influencing the quality of total mesorectal excision. Br J Surg 2012; 99:714-20.

15. Leite JS, Martins SC, Oliveira J, Cunha MF, Castro-Sousa F. Clinical significance of macroscopic completeness of mesorectal resection in rectal cancer. Colorectal Dis 2011; 13(4):381-6.

16. Maslekar S, Sharma A, Macdonald A, Gunn J, Monson JR, Hartley JE. Mesorectal grade predict recurrences after curative resection for rectal cancer. Dis Colon Rectum 2007; 50:168-75.

17. Rickles AS, Dietz DW, Chang GJ, Wexner SD, Berho ME et al. High rate of positive circumferential resection margins following rectal cancer surgery: A call to action. Ann Surg. 2015 Oct 14 [Epub ahead of print].

18. Bonjer HJ, Deijen CL, Abis GA, Cuesta MA, et al; COLOR II Study Group. A randomized trial of laparoscopic versus open surgery for rectal cancer. N Engl J Med 2015; 2;372(14):1324-32.

19. Lacy AM, García-Valdecasas JC, Delgado S, Castells A et al. Laparoscopy-assisted colectomy versus open colectomy for treatment of non-metastatic colon cancer: a randomised trial. Lancet 2002; 359:2224-9.

20. Veldkamp R, Kuhry E, Hop WC, Jeekel J et al. Laparoscopic surgery versus open surgery for colon cancer: short-term outcomes of a randomised trial. Colon cancer Laparoscopic or Open Resection Study Group (COLOR). Lancet Oncol 2005; 6:477-84.

21. Leung KL, Kwok SP, Lam SC, Lee JF, et al. Laparoscopic resection of rectosigmoid carcinoma: prospective randomised trial. Lancet 2004; 363(9416):1187-92.

22. Jayne DG, Guillou PJ, Thorpe H, Quirke P, et al; UK MRC CLASICC Trial Group. Randomized trial of laparoscopic-assisted resection of colorectal carcinoma: 3-year results of the UK MRC CLASICC Trial Group. J Clin Oncol 2007; 25(21):3061-8.

23. Ng SS, Leung KL, Lee JF, Yiu RY, et al. Long-term morbidity and oncologic outcomes of laparoscopic-assisted anterior resection for upper rectal cancer: ten-year results of a prospective, randomized trial. Dis Colon Rectum 2009; 52(4):558-66.

24. Quirke P, Durdey P, Dixon MF, Williams NS. Local recurrence of rectal adenocarcinoma due to inadequate surgical resection. Lancet 1986; 2:996-9.

25. Nagtegaal ID, van de Velde CJ, van derWorp E, Kapiteijn E, et al. Macroscopic evaluation of rectal cancer resection specimen: Clinical significance of the pathologist in quality control. J Clin Oncol 2002; 20:1729-34.

26. Campa-Thompson M, Weir R, Calcetera N, Quirke P, et al. Pathologic processing of the total mesorectal excision. Clin Colon Rectal Surg. 2015 Mar; 28(1):43-52.

27. Tsai BM, Finne CO, Nordenstam JF, Christoforidis D, et al. Transanal endoscopic microsurgery resection of rectal tumors: outcomes and recommendations. Dis Colon Rectum 2010; 53:16-23.

28. Langer C, Liersch T, Süss M, Siemer A, et al. Surgical cure for early rectal carcinoma and large adenoma: transanal endoscopic microsurgery (using ultrasound or electrosurgery) compared to conventional local and radical resection. Int J Colorectal Dis 2003; 18:222-9.

29. Lee W, Lee D, Choi S, Chun H. Transanal endoscopic microsurgery and radical surgery for T1 and T2 rectal cancer. Surg Endosc 2003; 17:1283-7.

30. Lim SB, Seo SI, Lee JL, Kwak JY, et al. Feasibility of transanal minimally invasive surgery for mid-rectal lesions. Surg Endosc 2012; 26:3127-32.

31. Cataldo PA, O'Brien S, Osler T. Transanal endoscopic microsurgery: a prospective evaluation of functional results. Dis Colon Rectum 2005; 48:1366-71.

32. Sylla P, Willingham FF, Sohn DK, Gee D, et al. NOTES rectosigmoid resection using transanal endoscopic microsurgery (TEM) with transgastric endoscopic assistance: a pilot study in swine. J Gastrointest Surg. 2008; 12:1717-23.

33. Sylla P, Sohn DK, Cizginer S, Konuk Y, et al. Survival study of natural orifice translumenal endoscopic surgery for rectosigmoid resection using transanal endoscopic microsurgery with or without transgastric endoscopic assistance in a swine model. Surg Endosc. 2010; 24:2022-30.

34. Telem DA, Han KS, Kim MC, Ajari I et al. Transanal rectosigmoid resection via natural orifice translumenal endoscopic surgery (NOTES) with total mesorectal excision in a large human cadaver series. Surg Endosc Surg Endosc 2013; 27(1):74-80.

35. Sylla P, Rattner DW, Delgado S, Lacy AM. NOTES transanal rectal cancer resection using transanal endoscopic microsurgery and laparoscopic assistance. Surg Endosc. 2010; 24:1205-10.

36. Sylla P, Bordeianou LG, Berger D, Han KS, et al. A pilot study of natural orifice transanal endoscopic total mesorectal excision with laparoscopic assistance for rectal cancer. Surg Endosc 2013; 27:3396-405.

37. Veltcamp Helbach M, Deijen CL, Velthuis S. Transanal total mesorectal excision for rectal carcinoma: short-term outcomes and experience after 80 cases. Surg Endosc 2016; 30:464-70.

38. Chouillard E, Chahine E, Khoury G, Vinson-Bonnet B, et al. NOTES total mesorectal excision (TME) for patients with rectal neoplasia: a preliminary experience. Surg Endosc. 2014; 28:3150-7.

39. Chen CC, Lai YL, Jiang JK, Chu CH, et al. The evolving practice of hybrid natural orifice transluminal endoscopic surgery (NOTES) for rectal cancer. Surg Endosc 2015; 29(1):119-26.

40. Serra-Aracil X, Mora-López L, Casalots A, Pericay C et al. Hybrid NOTES: TEO for transanal total mesorectal excision: intracorporeal resection and anastomosis. Surg Endosc. 2016; 30:346-54.

41. Lacy AM, Tasende MM, Delgado S, Fenandez-Hevia et al. Transanal total mesorectal excision for rectal cancer: Outcomes after 140 patients. J Am Coll Surg 2015; 221:415-23.

42. Burke JP, Martin-Perez B, Khan A, Nassif G et al. Transanal Total Mesorectal Excision for Rectal Cancer: early outcomes in 50 consecutive patients. Colorectal Dis. 2016 Jan 8. doi: 10.1111.

43. Tuech JJ, Karoui M, Lelong B, De Chaisemartin C, et al. A Step Toward NOTES Total Mesorectal Excision for Rectal Cancer: Endoscopic Transanal Proctectomy. Ann Surg. 2015; 261:228-33.

44. Rouanet P, Mourregot A, Azar CC, Carrere S, et al. Transanal endoscopic proctectomy: an innovative procedure for difficult resection of rectal tumors in men with narrow pelvis. Dis Colon Rectum 2013; 56:408-15.

45. de'Angelis N, Portigliotti L, Azoulay D, Brunetti F. Transanal total mesorectal excision for rectal cancer: a single center experience and systematic review of the literature. Langenbecks Arch Surg. 2015; 400:945-59.

46. Fernandez-Hevia M, Delgado S, Castells A, Tasende M, et al. Transanal Total Mesorectal Excision in Rectal Cancer: Short-term Outcomes in Comparison With Laparoscopic Surgery. Annals of surgery 2015; 261(2):221-7.

47. Velthuis S, Nieuwenhuis DH, Ruijter TE, Cuesta MA, et al. Transanal versus traditional laparoscopic total mesorectal excision for rectal carcinoma. Surg Endosc. 2014; 28(12):3494-9.

48. Kang L, Chen WH, Luo SL, Luo YX et al. Transanal total mesorectal excision for rectal cancer: a preliminary report. Surg Endosc 2015 Aug 27.

49. Muratore A, Mellano A, Marsanic P, De Simone M. Transanal total mesorectal excision (taTME) for cancer located in the lower rectum: short- and mid-term results. Eur J Surg Oncol 2015; 41:478-83.

50. Buchs NC, Nicholson GA, Yeung T, Mortensen NJ et al. Transanal rectal resection: an initial experience of 20 cases. Colorectal Dis 2016; 18:45-50.

51. Perdawood SK, Al Khefagie GA. Transanal vs laparoscopic total mesorectal excision for rectal cancer: initial experience from Denmark. Colorectal Dis 2016; 18:51-8.

52. Rullier E, Laurent C, Bretagnol F, Rullier, et al. Sphincter-saving resection for all rectal carcinomas. The end of the 2-cm distal rule. Ann Surg 2005; 241:465-9.

53. Schiessel R, Karner-Hanusch J, Herbst F, Teleky B, et al. Intersphincteric resection for low rectal tumours. Br J Surg 1994; 81:1376-8.

54. Chamlou R, Parc Y, Simon T, Bennis M, et al. Long-term results of intersphincteric resection for low rectal cancer. Ann Surg 2007; 246:916-21.

55. Marks J, Mizrahi B, Dalane S, Nweze I, et al. Laparoscopic transanal abdominal transanal resection with sphincter preservation for rectal cancer in the distal 3 cm of the rectum after neoadjuvant therapy. Surg Endosc 2010; 24:2700-7.

56. Gamagami R, Istvan G, Cabarrot P, Liagre A, et al. Fecal continence following partial resection of the anal canal in distal rectal cancer: long-term results after coloanal anastomoses. Surgery 2000; 127:291-5.

57. Bretagnol F, Rullier E, Laurent C, Zerbib F, et al. Comparison of functional results and quality of life between intersphincteric resection and conventional coloanal anastomosis for low rectal cancer. Dis Colon Rectum 2004; 47:832-8.

58. Penna M, Hompes R, Arnold S, Wynn G, Austin R, Warusavitarne J, Moran B, Hanna GB, Mortensen NJ, Tekkis PP; TaTME Registry Collaborative. Transanal Total Mesorectal Excision International Registry Results of the First 720 Cases. Ann Surg. 2016 Oct 4. [Epub ahead of print]

59. Breukink S, Grong a, Pierie J, Hoff C, et al. Laparoscopic versus open total mesorectal excision for rectal cancer: An evaluation of the mesorectum's macroscopic quality. Surgical Endoscopy 2005; 19:307-10.

60. Marks JH, Valsdottir EB, Rather AA, Nweze IC, et al. Fewer than 12 lymph nodes can be expected in a surgical specimen after high-dose chemoradiation therapy for rectal cancer. Dis Colon Rectum. 2010; 53:1023-9.

61. Marsh PJ, James RD, Schofield PF. Definition of local recurrence after surgery for rectal carcinoma. Br J Surg 1995; 82:465-8.

62. Smith JJ, Chow OS, Gollub MJ, Nash GM et al. Organ Preservation in Rectal Adenocarcinoma: a phase II randomized controlled trial evaluating 3-year disease-free survival in patients with locally advanced rectal cancer treated with chemoradiation plus induction or consolidation chemotherapy, and total mesorectal excision or nonoperative management. BMC Cancer 2015; 23:15:767.

63. Jorge JM, Wexner SD. Etiology and management of fecal incontinence. Dis Colon Rectum. 1993; 36:77-97.

64. Bakx R, Sprangers MA, Oort FJ, van Tets WF, et al. Development and validation of a colorectal functional outcome questionnaire. Int J Colorectal Dis. 2005; 20:126-36.

65. Rockwood TH. Incontinence Severity and QOL Scales for Fecal Incontinence. Gastroenteroloty 2004; 126:S106-S113.

66. Rosen R, Brown C, Heiman J, Leiblum S, et al. The Female Sexual Function Index (FSFI): a multidimensional self-report instrument for the assessment of female sexual function. J Sex Marital Ther 2000; 26:191-208.

67. Rosen RC, Riley A, Wagner G, Osterloh IH, et al. The international index of erectile function (IIEF): a multidimensional scale for assessment of erectile dysfunction. Urology 1997; 49:822-30.

68. Barry MJ, Fowler FJ Jr, O'Leary MP, Bruskewitz RC, et al. The American Urological Association symptom index for benign prostatic hyperplasia. The Measurement Committee of the American Urological Association.J Urol 1992; 148:1549-57.

69. Lustosa SA, Matos D, Atallah AN, Castro AA. Stapled versus handsewn methods for colorectal anastomosis surgery. Cochrane Database Syst Rev 2001; CD003144.

70. Merad F, Hay JM, Fingerhut A, Yahchouchi E, et al. Is prophylactic pelvic drainage useful after elective rectal or anal anastomosis? A multicenter controlled randomized trial. French Association for Surgical Research. Surgery. 1999; 125:529-35.

71. Jayne DG, Brown JM, Thorpe H, Walker J, et al. Bladder and sexual function following resection for rectal cancer in a randomized clinical trial of laparoscopic versus open technique. Br J Surg 2005; 92:1124-32.

72. Dindo D, Demartines N, Clavien, P. Classification of surgical complications. A new proposal with evaluation in a cohort of 6336 patients and results of a survey. Ann Surg 2004; 240(2):205-213.

73. Rullier A, Laurent C, Vendrely V, et al. Impact of colloid response on survival after preoperative radiotherapy in locally advanced rectal carcinoma. Am J Surg Pathol 2005; 29:602–606.

74. Reynolds IS, Boland MR, Reilly F, Deasy A, Majeed MH, Deasy J, Burke JP, McNamara DA. C-reactive protein as a predictor of anastomotic leak in the first week after anterior resection for rectal cancer. Colorectal Dis. 2017 Sep; 19 (9): 812-818

## *APPENDIX A: PERFORMANCE STATUS CRITERIA*

<table>
<tr><th colspan="2">ECOG Performance Status Scale</th><th colspan="2">Karnofsky Performance Scale</th></tr>
<tr><td>Grade</td><td>Descriptions</td><td>Percent</td><td>Description</td></tr>
<tr><td rowspan="2">0</td><td rowspan="2">Normal activity. Fully active, able to carry on all pre-disease performance without restriction.</td><td>100</td><td>Normal, no complaints, no evidence of disease.</td></tr>
<tr><td>90</td><td>Able to carry on normal activity; minor signs or symptoms of disease.</td></tr>
<tr><td rowspan="2">1</td><td rowspan="2">Symptoms, but ambulatory. Restricted in physically strenuous activity, but ambulatory and able to carry out work of a light or sedentary nature (e.g., light housework, office work).</td><td>80</td><td>Normal activity with effort; some signs or symptoms of disease.</td></tr>
<tr><td>70</td><td>Cares for self, unable to carry on normal activity or to do active work.</td></tr>
<tr><td rowspan="2">2</td><td rowspan="2">In bed <50% of the time. Ambulatory and capable of all self-care, but unable to carry out any work activities. Up and about more than 50% of waking hours.</td><td>60</td><td>Requires occasional assistance, but is able to care for most of his/her needs.</td></tr>
<tr><td>50</td><td>Requires considerable assistance and frequent medical care.</td></tr>
<tr><td rowspan="2">3</td><td rowspan="2">In bed >50% of the time. Capable of only limited self-care, confined to bed or chair more than 50% of waking hours.</td><td>40</td><td>Disabled, requires special care and assistance.</td></tr>
<tr><td>30</td><td>Severely disabled, hospitalization indicated. Death not imminent.</td></tr>
<tr><td rowspan="2">4</td><td rowspan="2">100% bedridden. Completely disabled. Cannot carry on any self-care. Totally confined to bed or chair.</td><td>20</td><td>Very sick, hospitalization indicated. Death not imminent.</td></tr>
<tr><td>10</td><td>Moribund, fatal processes progressing rapidly.</td></tr>
<tr><td>5</td><td>Dead</td><td>0</td><td>Dead</td></tr>
</table>

## APPENDIX B: PATHOLOGY TEMPLATE FOR RECTAL TME ASSESSMENT

| | |
|---|---|
| Subject Registration Number: ________________ | Institution Name: ____________ |
| Subject Initials: _____________ | Date of Surgery: ____________ |

**TEMPLATE FOR SYNOPTIC REPORT OF RECTAL TME**

**Adenocarcinoma of the rectum final diagnosis synoptic report**

1. Rectum, total/partial mesorectal excision: Adenocarcinoma of the rectum (_______ cm).
   *See note for synoptic report (specific site)*

**Note: ____________________________________________________________**

**_________________________________________________________________**

2. Gross evaluation of the specimen determined that the mesorectal excision was:
   □ Complete □ Near-complete □ Incomplete

**[TNM] SYNOPTIC REPORT**

1. Tumor Stage Summary :*(circle y or r, if appropriate)*
   a. Select ‘r’ or ‘y’ if appropriate □ r □ y □ N/A
   b. pT ______ N ______ M ______

*(Include the suffix “m” after the T stage if multiple primaries; e.g., pT3 (m)*

2. Specific Site: _________________________________
3. Tumor Size (greatest dimension):___________cm
4. Tumor Grade:

   □ Low Grade (Well differentiated or Moderately differentiated)

   □ High Grade (Poorly differentiated or Undifferentiated)

5. Extent of Invasion

   □ pT0 (No Tumor present)

   □ pT1 (Tumor invades submucosa)

   - SMI
   - SM2

- SM3

□ pT2 (Tumor invades muscularis propria)

□ pT3 (Tumor invades through the muscularis propria into perirectal tissues)

□ pT4b (Tumor directly invades other organs or structures): ______________________

6. Tumor Regression Grade [CAP]

□ Grade 0 (complete response, no viable cancer cells)

□ Grade 1 (moderate response, single cells or small groups of cancer cells)

□ Grade 2 (minimal response, residual cancer outgrown by fibrosis)

□ Grade 3 (poor response, minimal or no tumor kill; extensive residual cancer)

7. Lymphovascular Invasion

□ Absent

□ Present

□ Suspicious

8. Perineural Invasion

□ Absent

□ Present

9. Large Extramural Venous Invasion

□ Absent

□ Present

□ Suspicious

10. □ Proximal Margin

□ Uninvolved

(Surgical clearance: __________ cm, tumor to proximal margin)

□ Involved

11. Distal Margin

□ Uninvolved

(Surgical clearance: __________ cm, tumor to distal margin)

□ Involved

12. Radial or Mesenteric Margin

□ Uninvolved

(Surgical clearance: __________ cm, tumor to radial margin)

□ Involved

- as a result of finding of tumor nodule
- as the result of direct tumor extension into the margin

13. Regional Lymph Nodes

□ pNx (Cannot be assessed)

□ pN0 (No regional lymph node metastasis):

_____ *nodes examined.*

□ pN1a (Metastasis in 1 regional lymph node):

*1 node POSITIVE out of _____ nodes examined.*

□ pN1b (Metastasis in 2 to 3 regional lymph nodes):

_____ *nodes POSITIVE out of* _____ *nodes examined.*

□ pN1c (Tumor deposit[s] in the perirectal tissues without regional nodal metastasis):

_____ *deposits;* _____ *nodes examined.*

□ pN2a (Metastasis in 4 to 6 regional lymph nodes):

_____ *nodes POSITIVE out of* _____ *nodes examined.*

□ pN2b (Metastasis in 7 or more regional lymph nodes):

_____ *nodes POSITIVE out of* _____ *nodes examined.*

14. Distant Metastases

□ pMx (metastasis cannot be assessed)
□ pM0 (No distant Metastasis)
□ pM1a(Metastasis confined to one organ or site): ______________
□ pM1b(Metastases in more than one organ/site or the peritoneum): __________

* Following long course neoadjuvant CRT, when mucin pools without tumor cells were found near or at the margin, CRM should be considered as negative[73]

Pathologist Initials : ____________________ Date : ______________

# Administrative Cover Sheet

# Multicenter Phase II Study of Transanal Total Mesorectal Excision (taTME) with Laparoscopic Assistance for Rectal Cancer

**Study Type**

Investigator Initiated

Addendum 1

**Protocol Version 6**

**Coordinating Center**

Mount Sinai Hospital

1 Gustave L. Levy Place Box 1259

New York, NY 10029

**DSMC**

Tisch Cancer Center DSMC

**External Monitor**

Biomedical Research Alliance of New York

**SPONSORS**

Society of the American Gastrointestinal and Endoscopic Surgeons (Administrative Sponsor)
American Society of Colon and Rectal Surgeons Research Foundation Grant

**Overall PI**

Patricia Sylla, MD

# Clinical Study Protocol Addendum 1

## Multicenter Phase II Study of Transanal Total Mesorectal Excision (taTME) with Laparoscopic Assistance for Rectal Cancer

**Short Title**

Multicenter Phase II Study of taTME

Addendum 1

**Protocol Version 6**

**Coordinating Center**

Mount Sinai Hospital

1 Gustave L. Levy Place Box 1259

New York, NY 10029

**Overall PI**

Patricia Sylla, MD

## **Protocol Addendum**

The purpose of this addendum is to provide documentation in support of continuation of research activities of the Multicenter Phase II Study of Transanal Total Mesorectal Excision (taTME) With Laparoscopic Assistance for Rectal Cancer, Protocol # GCO 16-2009, during the COVID-19 Pandemic. The COVID-19 Pandemic has created a public health crisis throughout the United States and other parts of the world. To limit exposure, only essential and emergent in-person clinical encounters are allowed at most health care facilities. Ensuring the safety of current study participants, as well as the safety of clinical site staff members and physicians, is of the utmost importance. Therefore, modifications to study conduct must be made in order to ensure these precautions are in place.

The information in this addendum relates to the modifications to be implemented for the following protocol-specified procedures:

1. Screening and Enrollment
2. Scheduled follow-up visits
    a) Postoperative visits
    b) Oncologic Follow up visits
3. Completion of Quality of Life questionnaires

This addendum shall remain in effect until the end of COVID-19 pandemic-related disruptions in clinical operations. Research activities, shall restart only at the time that is deemed safe and appropriate for participating clinical sites to return to the schedule outlined in the original protocol, per their individual institutions' and IRBs' policies and regulations. This addendum is a continuation of the one that was effective as of March 15, 2020.

**1) SCREENING AND ENROLLMENT**

Enrollment in the clinical study is still ongoing. Due to the limited resources at many hospitals and clinics at this time, and to ensure patient safety, it is expected that many participating institutions will opt to perform only urgent and emergent surgeries and hence postpone all elective procedures during this period. Therefore, it is anticipated that screening and enrollment of new subjects will either slow down or not occur during this time period, and originally projected study duration timelines will be extended accordingly.

If study site surgeons are evaluating subjects for potential eligibility for enrollment in the trial, they may do so either during in-person or virtual (telehealth) evaluation of the subject.

**Informed Consent**

When possible, screening of new subjects will continue, but informed consent forms must still be signed and dated by the subject, as well as administered, signed, and dated by the person delegated to perform this duty.

The consenting process may be conducted virtually (phone call/telehealth visit), if the approved informed consent forms have been mailed/emailed to potential subjects ahead of the discussion. Subjects wishing to participate in the study may sign and date consents and email them back to the site, or a verbal consent may be

documented at the time the consenting process takes place virtually, and the paper informed consent form can be signed and dated when the subject presents in person.

2) **FOLLOW-UP VISITS**

Study subjects may not be able to come to the investigational sites for the protocol-specified in-person postoperative follow-up visits and alternative methods for collecting follow-up data may be necessary. Alternative methods include phone or video/virtual visits.

a) **Postoperative Visits**

Postoperative visits #1, #2, #3 and #4 can be conducted as a telehealth visits. During the visit, surgeons will assess whether a subject has developed any AE or SAE and document them accordingly in the case report form. Definitive in-person evaluation and/or testing to confirm or rule out potential non-life-threatening complication may need to be deferred at institutions severely impacted by the pandemic. In such cases, delays in in-person postoperative visits will not be considered as protocol deviations, but will rather be incorporated as part of this addendum, unless otherwise specified by the participating institution's IRB.

b) **Oncologic follow-up Visits**

Oncologic follow-up visits can be conducted as a telehealth visit. When feasible, subjects will continue to obtain surveillance CEA and CT scans according to the study protocol schedule. Endoscopic surveillance however, will be deferred until in-person evaluation and elective endoscopy can be safely resumed at study sites severely impacted by the COVID-19 crisis. Delays in oncologic follow-up visits under those circumstances will not be considered as protocol deviations, but will rather be incorporated as part of this addendum, unless otherwise specified by the participating institution's IRB

**Study calendar**

| | Screening | Prior to neoadjuvant Tx (if applicable) | REGISTRATION | Preop | SURGERY | Postop | | | | | | |
|---|---|---|---|---|---|---|---|---|---|---|---|---|
| | | | | | | Wk 1-2 | Wk 4-6 | Year 1 | Year 2 | Year 3 | Year 4 | Year 5 |
| ICF | X | | | | | | | | | | | |
| Surgical Consent for study procedure | | | | X | | | | | | | | |
| Clinical Evaluation (BMI, DRE, distance from anal verge) | X | | | X[A] | | | | | | | | |
| Proctoscopy | X | | | | | | | Q 3-6 months[f] | | Q 6 months[f] | | |
| H&P | X | | | X[B] | | X | X | Q 3-6 months[f] | | Q 6 months[f] | | |
| CEA | X | | | | | | | Q 3-6 months[f] | | Q 6 months[f] | | |
| Colonoscopy | X | | | | | | | X[f] | | | X[f] | |
| CT (chest, abdomen and pelvis) | X[c] | | | | | | | Q 6-12 months[f] | | | | |
| Pelvic MRI | X[c] | | | | | | | | | X | | |
| Performance Status | X | | | | | | | | | | | |

| | | | | | | | | | | | | |
|---|---|---|---|---|---|---|---|---|---|---|---|---|
| Labs[d] | | | | X[b] | | X | | | | | | |
| Operative Report | | | | | | X | | | | | | |
| Histopathology | X | | | | | | X | | | | | |
| Postoperative Complications | | | | | | X | X | Q 4-6 months | | | | |
| Oncologic FU[f] | | | | | | | | Q 3-6 months | | Q 6 months | | |
| Questionnaires[e] | | X[e1] | | X[e2] | | | | X[e3] | X[e4] | | | |
| AE and SAE evaluation | | | | | | From index surgery throughout the rest of the study | | | | | | |

e. The tumor may be reassessed post-neoadjuvant treatment to reconfirm eligibility.
f. Repeat Labs, H&P and vitals preoperatively if pre-registration labs/H&P/Vitals were performed more than 30 days prior to surgery.
g. Imaging, colonoscopy and rectal tumor biopsy performed up to 3 months prior to screening (or prior to start of neoadjuvant treatment, in subjects enrolled during or at completion of neoadjuvant treatment) can be used to determine eligibility.
h. CBC, PT/PTT/INR, sodium, potassium, chloride, CO2, BUN, creatinine, glucose, albumin, and pregnancy test (in childbearing potential age patients)
i. Questionnaires (Wexner, FIQL, COREFO, IPSS, IIEF, or FSFI) are administered prior to start of neoadjuvant treatment, if applicable (e1); before study procedure or following completion of neoadjuvant treatment (e2); 6-8 months postoperatively in non diverted subjects or 3-4 months following ileostomy closure (e3); 12-18 months following study procedure or later in delayed ileostomy closures (e4)
j. During the COVID-9 pandemic disruption, postop visits and oncologic follow-up visits can be conducted as telehealth visits (please refer to details detailed above in postoperative plan section

### 3) QOL QUESTIONNAIRE COMPLETION

Quality of Life (QOL) questionnaires can be emailed trough RedCap to the subjects that have opted for the email version. In this case there should be no changes to the regular standard SOP. For subjects that have opted for paper version at the time of visits, questionnaires forms should be emailed (if the subject opts to share email address with study team and not RedCap) or mailed to the subject before the follow-up visit is conducted, and the subject should print and complete the form on the day of the actual visit. Completed QOL questionnaires may be scanned and emailed or sent via smart phone to coordinators. If subjects are unable or do not have access to printers, scanners, and/or smart phones, coordinators may record subject responses over the phone, but only when other methods are not feasible. In these instances, coordinators should review all recorded responses with the subject, ask for verbal confirmation, and document that confirmation on the source worksheet.

## REPORTING

It is important for participating sites to keep a record of which alternatives to protocol-specified procedures are being used for which subjects during this time. Specifics including subject ID, assessment, and/or follow-up visit time point within the parameters of this addendum will need to be reported for potential impact on statistical analyses and at the close of the study.

Throughout the COVID-19 pandemic, please record all modifications to the protocol-specified procedures on a tracker, which will be collected by the coordinating center at the end of the COVID-19 pandemic disruptions and will serve as a collective PD report for the site. Please report the PD to local IRB as per local regulations.

## OTHER CONSIDERATIONS

Please continue to adhere to IRB regulations and good clinical practices. The Principal Investigators at the participating sites remain ultimately responsible for the conduct of the study and can determine the most appropriate methods for their individual institutions. Additional modifications may become necessary if the pandemic extends beyond .
